# Structure-Based Optimization and Biological Evaluation of Pancreatic Lipase Inhibitors as Novel Potential Antiobesity Agents

**DOI:** 10.1007/s13659-015-0062-6

**Published:** 2015-06-18

**Authors:** Kun Wei, Gang-Qiang Wang, Xue Bai, Yan-Fen Niu, He-Ping Chen, Chun-Nan Wen, Zheng-Hui Li, Ze-Jun Dong, Zhi-Li Zuo, Wen-Yong Xiong, Ji-Kai Liu

**Affiliations:** State Key Laboratory of Phytochemistry and Plant Resources in West China, Kunming Institute of Botany, Chinese Academy of Sciences, Kunming, 650201 China; School of Nuclear Technology and Chemistry & Biology, Hubei University of Science and Technology, Xianning, 437100 China

**Keywords:** Natural products, Structure optimization, Antiobesity agents, Pancreatic lipase inhibitors, Vibralactone derivatives

## Abstract

The unusual fused *β*-lactone vibralactone was isolated from cultures of the basidiomycete *Boreostereum vibrans* and has been shown to significantly inhibit pancreatic lipase. In this study, a structure-based lead optimization of vibralactone resulted in three series of 104 analogs, among which compound **C1** exhibited the most potent inhibition of pancreatic lipase, with an IC_50_ value of 14 nM. This activity is more than 3000-fold higher than that of vibralactone. The effect of compound **C1** on obesity was investigated using high-fat diet (HFD)-induced C57BL/6 J obese mice. Treatment with compound **C1** at a dose of 100 mg/kg significantly decreased HFD-induced obesity, primarily through the improvement of metabolic parameters, such as triglyceride levels.

**Electronic supplementary material:**

The online version of this article (doi:10.1007/s13659-015-0062-6) contains supplementary material, which is available to authorized users.

## Introduction

Obesity represents a physiological disorder in which there is a chronic imbalance between increased food intake and energy expenditure, resulting in increased deposition of fat within body tissues. Obesity is emerging as one of the greatest threats to global health in this century, with more than 1.5 billion overweight adults worldwide. The World Health Organization (WHO) predicted that this number will increase to approximately 3.3 billion by 2030 [1, 2]. Obesity is associated with an increased risk of diabetes, hypertension, cardiovascular disorders, and renal diseases, afflicting 40 % of overweight adults [3, 4]. Obesity can no longer be considered a problem of only developed, wealthy countries. Developing countries worldwide are experiencing similar increases in the prevalence of overweight individuals and obesity within their populations, particularly in urban regions [1]. Obesity is recognized as a global epidemic disease and a significant health care burden. The market for antiobesity drugs is potentially large, accounting for 2–6 % of the total health care costs in several developed countries, and the obesity market has been predicted to grow continuously [5].

Energy intake begins from fat absorption through the digestion of fat into monoglycerides and fatty acids. Lipase is a key enzyme for lipid absorption. Among lipases, pancreatic lipase is responsible for the hydrolysis of 50–70 % of total dietary fats [5]. The reduction of fat absorption through pancreatic lipase inhibition is known to benefit the regulation of obesity [6–9].

Two drugs, orlistat (a lipase inhibitor) and sibutramine (an appetite suppressant), were used for the antiobesity [10]. These drugs are limited in their use due to their severe side effects, sibutramine has been withdrawn in 2010 duo to increased cardiovascular events from the market in countries including Australia, Canada, China, the United Kingdom, and the United States [11–13]. Therefore, reliable and effective antiobesity drugs are urgently required.

**Fig. 1:**
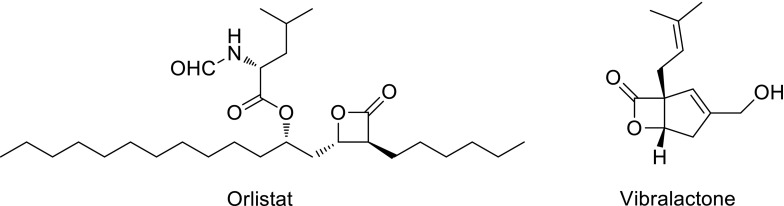
The structures of orlistat and vibralactone

Orlistat forms a covalent but reversible bond with the active site serine residue of pancreatic lipase, rendering it unable to hydrolyze dietary fat into free fatty acids, therefore reducing the absorption of dietary fat [14]. We have previously reported the isolation of the unusual fused *β*-lactone vibralactone from cultures of the basidiomycete *Boreostereum vibrans*, which exhibited significant potency as a pancreatic lipase inhibitor [15]. Our ongoing investigations on the chemical constituents of the cultures of *B. vibrans* have led to a series of reports on bioactive vibralactone derivatives [16–22]. Zhou and Snide developed an elegant 10-step chemical route for the total synthesis of (±)-vibralactone and (−)-vibralactone C [23, 24]. The Sieber group established that the unusual fused *β*-lactone bicyclic system of vibralactone may account for the binding of both types of caseinolytic peptidases that are vital for bacterial virulence [25, 26]. Our recent investigation elucidated the biosynthetic pathway, which includes several interesting reactions that may involve unusual enzymes [27]. As shown in Fig. 1, the structure of vibralactone is interesting because it bears similarities to orlistat, which is a natural *β*-lactone-type lipase inhibitor. The pancreatic lipase inhibitory activity of vibralactone is most likely due to the *β*-lactone pharmacophore.

To further explore the potential of this unique molecule, a large-scale fermentation of the fungus *B. vibrans* was performed, and a large amount of vibralactone was isolated. Using the isolated vibralactone as the starting material, molecular modeling, chemical synthesis and biological evaluation were used to optimize the structure of vibralactone against pancreatic lipase. A study was performed to investigate the interactions between vibralactone and human pancreatic lipase. Three key subsites from the crystal structure of human pancreatic lipase were identified in the catalytic site, with which vibralactone interacts. In this study, three series of 104 analogs of vibralactone derivatives were designed and synthesized. All of the synthesized compounds were evaluated for their inhibitory activities against pancreatic lipase in vitro. Compound **C1** exhibited the most potent inhibitory activity against pancreatic lipase, with an IC_50_ value of 14 nM. This activity is more than 3000-fold higher than that of vibralactone. Compound **C1** was selected for further in vivo evaluation. The effect of compound **C1** on obesity was investigated in high-fat diet (HFD)-induced C57BL/6 J obese mice. Compound **C1** was administered at a dose of 100 mg/kg for 33 days. The antiobesity activity was evaluated by measuring the body weight, epididymal white adipose tissue and metabolic plasma parameters. On day 33, the body weight of the compound **C1**-treated group was significantly lower compared with that of the HFD-treated group (model group). The metabolic parameters that increased in the HFD group were reduced following administration of compound **C1**. Particularly, the increased triglyceride levels were significantly reduced in the compound **C1**-treated group. These results indicate that treatment with compound **C1** significantly decreased HFD-induced obesity, primarily through the improvement of metabolic parameters, such as triglycerides. Therefore, compound **C1**, as a potent pancreatic lipase inhibitor, demonstrates potential benefits in the regulation of obesity.

## Chemistry

**Scheme 1:**
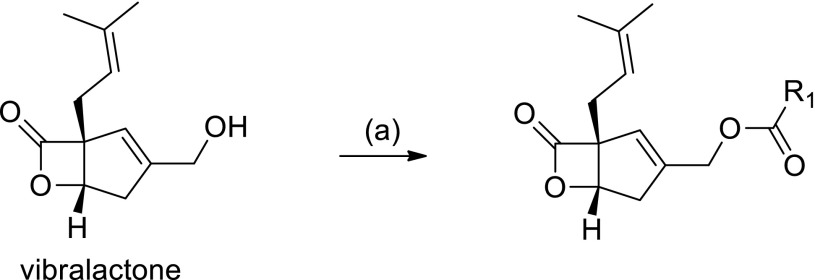
Synthesis of Compounds **A1**–**A54**. Reaction conditions: **a** R_1_COCl, NEt_3_, CH_2_Cl_2_, 0 °C

Compounds **A1**–**A54** were prepared as described in Scheme 1. A single long carbon chain was incorporated into the structure of vibralactone. A series of derivatives were synthesized using commercially available carboxyl acids with thionyl chloride in anhydrous dichloromethane. The corresponding acyl chlorides were generated and subsequently treated with vibralactone in the presence of triethylamine to yield compounds **A1**–**A54** (Scheme 1).

Compounds **A1**–**A54** were then evaluated for their bioactivity to inhibit pancreatic lipase. Each test was performed in triplicate, and the IC_50_ values were calculated based on the amount of inhibitor required to produce 50 % inhibition compared with the DMSO vehicle control [28]. The results are summarized in Table 1.

**Table 1:** In vitro pancreatic lipase inhibitory activities of compounds **A1**–**A54**

**Scheme 2:**
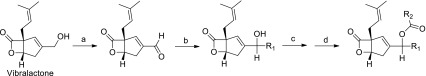
Synthesis of Compounds **B1**–**B37**. Reaction conditions: **a** PCC, CH_2_Cl_2_, 0 °C, 85 %; **b** R_1_MgBr, Et_2_O, −78 °C to 0 °C, 75 % (diastereoselectivity 1:1); **c** Mosher method; **d** R_2_COCl, NEt_3_, CH_2_Cl_2_, 0 °C

Compounds **B1**–**B37** were prepared as described in Scheme 2. Vibralactone underwent oxidation to the corresponding aldehyde with pyridinium chlorochromate (PCC), followed by treatment with Grignard reagent to yield the secondary alcohol as a mixture of two diastereoisomers. Preparative HPLC separation was performed, and the two pure diastereoisomers were isolated. The absolute stereochemistry of the products was assigned using the Mosher method [29, 30]. A series of vibralactone derivatives containing two long carbon chains was synthesized using commercially available carboxyl acids under standard conditions to generate the corresponding acyl chlorides, which were subsequently treated with the corresponding secondary alcohol in the presence of triethylamine to yield compounds **B1**–**B37**.

**Scheme 3:**
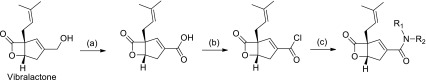
Synthesis of Compounds **C1**–**C13**. Reaction conditions: **a** Jones reagent, 0 °C, 90 %; **b** (COCl_2_), CH_2_Cl_2_, 0 °C; **c** R_1_NHR_2_, NEt_3_, CH_2_Cl_2_, 0 °C

We further designed the third series of vibralactone derivatives (**C1**–**C13**), which contained one amide bond. The structures of these derivatives and their synthetic route are shown in Scheme 3. Vibralactone underwent oxidation to the corresponding carboxyl acid with the Jones reagent, followed by treatment with (COCl_2_) to generate the corresponding acyl chloride, which was subsequently treated with commercially available secondary amines in the presence of triethylamine to yield the *N,N*-dialkyl amide derivatives [31].

## Results and Discussion

### Biological Evaluation

The first series of compounds (**A1**–**A54**) was less potent than the other two series but exhibited significant pancreatic lipase inhibitory activities (Table 1). Compound **A1** exhibited the highest inhibitory activity of this series, with an IC_50_ value of 0.083 μM, whereas the IC_50_ value of vibralactone is 47.26 μM. Vibralactone derivatives are active site-directed inhibitors that form stoichiometric long-lived acyl-enzyme complexes with pancreatic lipase following nucleophilic attack by the catalytic serine residue on the *β*-lactone group. An appropriately long carbon chain that enhances a compound’s solubility in oil causes it to partition between the oil core and the lipid-water interface. Increasing the interfacial area through an emulsification process promotes a stronger diffusion of the compound from the oil core toward the interface.

As shown in Table 2, the second series of vibralactone derivatives was substantially more potent than the first series. Compound **B1** exhibited the highest inhibitory activity of the second series, with an IC_50_ value of 0.030 μM against pancreatic lipase. Several other compounds (compounds **B1–B9**) in this series exhibited higher pancreatic lipase inhibitory activities than that of compound **A1**, which was the best inhibitor of the first series. The rationale for the design of this second series of derivatives was to include two appropriately positioned long carbon chains to effectively occupy the hydrophobic pocket. This molecular design strategy may lead to molecules with enhanced pharmacological properties.

**Table 2:** In vitro pancreatic lipase inhibitory activities of compounds **B1**–**B37**

The pancreatic lipase inhibitory activities of the third series of vibralactone derivatives are summarized in Table 3. Compound **C1** exhibited the highest inhibitory activity among the three series, with an IC_50_ value of 0.014 μM. Notably, two other compounds in this series, compounds **C2** and **C3**, exhibited even higher inhibitory activities than compound **A1**.

**Table 3:** In vitro pancreatic lipase inhibitory activities of compounds **C1**–**C13**

### Effects of Compound **C1** on High-Fat Diet-Induced Obese Mice

**Fig. 2:**
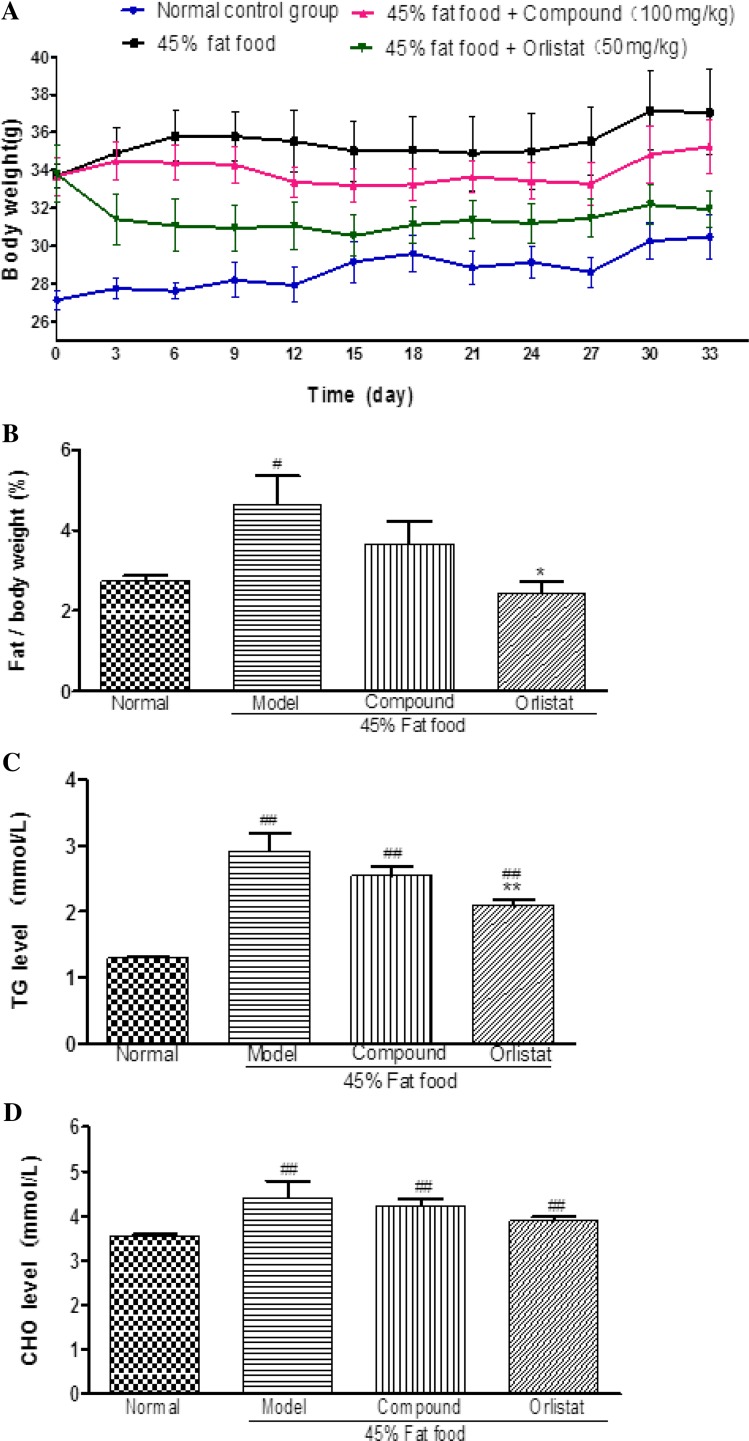
Compound **C1** reduces the body weight and fat tissue weight in obese mice. **a** During the 33-day experimental period, a dose of 100 mg/kg compound **C1** resulted in weight loss, as shown in *red*. **b** The normalized percentile weight of isolated fat to the total body weight. The fat tissue significantly decreased following treatment with either compound **C1** or orlistat (n = 4–5). The results are expressed as the mean ± SEM. **c** The TG level decreased following treatment with compound **C1** (n = 13–15). **d** The CHO level decreased following administration of compound **C1** (n = 12–15). #p < 0.05, ##p < 0.01 compared with the control group that was fed a normal diet; *p < 0.05, **p < 0.01 compared with control mice fed a 45 % fat diet

The most active compound, **C1**, was selected for further study in vivo to evaluate its ability to cause body weight loss and to analyze possible side effects in mice [32]. After one month of continuous administration of either compound **C1** (100 mg/kg) or orlistat (50 mg/kg), the total body weight (Fig. 2a) and the fat weight (epididymal white adipose tissue, Fig. 2b) of treated mice gradually decreased during this period, indicating that compound **C1** can reduce the body weight of HFD-induced obese mice.

The levels of triacylglyceride (TG) and cholesterol (CHO) are typically higher in obese animals. Lower levels of these two markers are considered important indicators for weight loss. Therefore, we further determined these levels in mice treated with compound **C1** or orlistat and observed that these levels significantly decreased (Fig. 2c, d, respectively), further supporting that compound **C1** reduced the body weight along with the TG and CHO levels.

Consistent with these in vitro results, compound **C1**, despite having a lower activity than that of orlistat in vivo, demonstrated efficacy in this model, with positive effects on weight loss throughout the duration of the studies without toxicity or adverse behavioral effects (up to 400 mg/kg, data not shown).

### Molecular Modeling Studies

To gain further insight into the binding mode of the reported compounds, a series of docking experiments was performed on pancreatic lipase. In studies by Wang et al. [33, 34] the “docking power” reported the comparison between 20 scoring functions in terms of the ability to reproduce the cocrystallized binding conformation of the ligand for a set of 195 different crystal structures from the Protein Data Bank (PDB). The ChemPLP scoring function in the commercial docking software GOLD [35] is evaluated in the top-ranked list to reproduce the binding conformation of the ligand. The docking experiments were performed on pancreatic lipase (PDB code: 1LPB) using the ChemPLP scoring function. The 2.46 Å resolution crystal structure included the pancreatic lipase-colipase in complex with a **C11** alkyl phosphonate inhibitor [36] in which pancreatic lipase adopts an active conformation with the β5 loop positioned away from the catalytic site. Hydrogen atoms were added, and water molecules that cocrystallized with the protein were removed from the original structure. This modified crystal structure of pancreatic lipase was used as the target for docking simulations using GOLD 5.2.2 software (CCDC, Cambridge, U.K.). The active site radius is 15 Å from OG atom 2376 of Ser153, which is one of the key residues in this serine protease. Nucleophilic attack on the *β*-lactone ring, which is the pharmacophore for this type of inhibitor, by the lipase active site serine residue is thought to form the long-lived acyl-enzyme complex. Therefore, a restraint was used to limit the distance between the carbonyl carbon and the oxygen in Ser153. The “Library screening” parameters and 30 GA runs were used for each ligand.

**Fig. 3:**
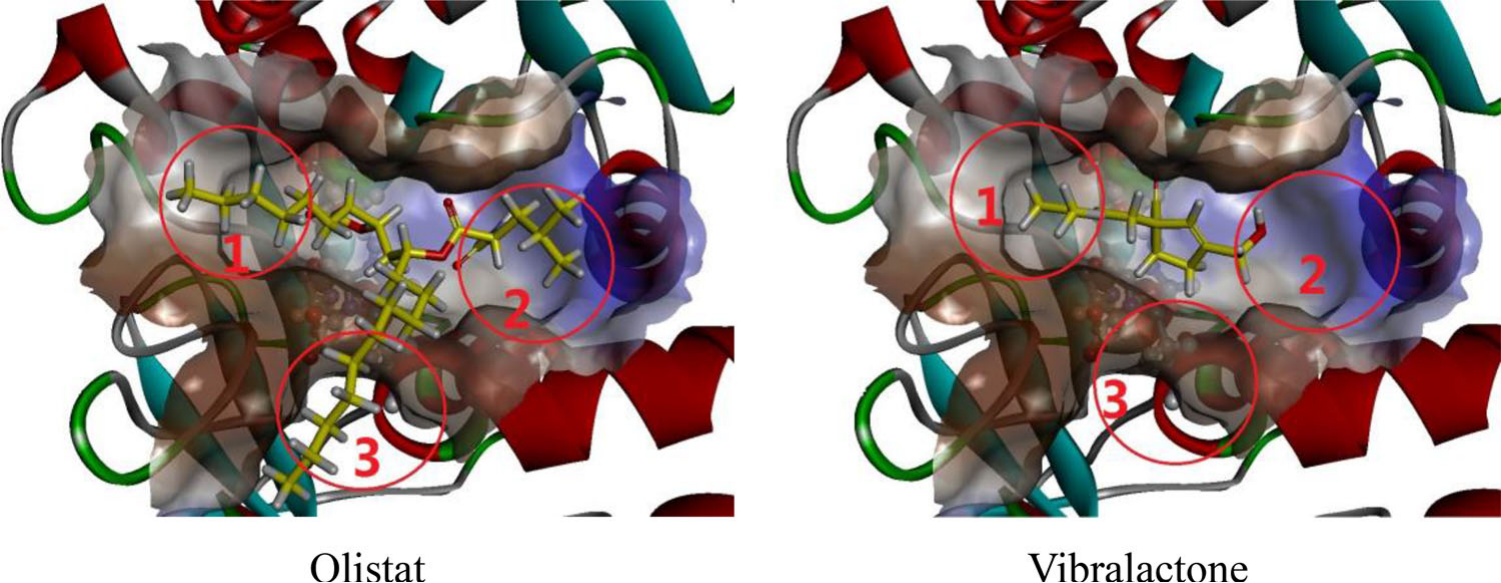
The potential binding conformations of orlistat and vibralactone in the binding site of pancreatic lipase

To understand the interactions between the inhibitors and pancreatic lipase, the first round of docking simulations was performed using orlistat and vibralactone versus the crystal structure of pancreatic lipase. Figure 3 shows the binding conformation of orlistat (left) and vibralactone (right) in the active site. Three subsites in the catalytic site (indicated by three red circles) were found to be important for the interaction with the inhibitors. Subsites 1 and 3 comprise hydrophobic residues, whereas subsite 2 consists of hydrophobic residues and the charged residues Asp79, Glu83 and Arg256 (blue surface). As observed in the binding mode of orlistat, a good inhibitor should extensively interact with all three subsites. The binding mode obtained from the docking simulation was further used for structure optimization of vibralactone. Based on the docking results, the structure of vibralactone has space to expand in the three directions to optimize inhibitor interactions with pancreatic lipase. Therefore, the structure of vibralactone was accordingly optimized.

**Fig. 4:**
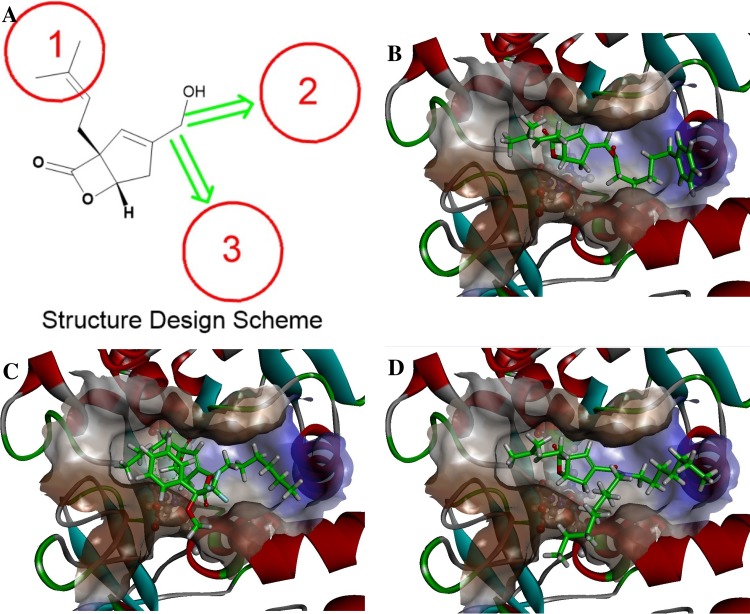
The structure optimization scheme and the docking mode in the active site for the newly designed and synthesized compounds. **a** The scheme to design new derivatives. **b** The binding conformation of compound **A1** in the active site of pancreatic lipase. **c** The docking conformation of newly synthesized compound **B1** in the binding pocket. **d** The new compound **C1** in the binding site of pancreatic lipase

The vibralactone derivatives **A1**–**A54** containing one long carbon chain toward subsite 2 were designed and synthesized, and the corresponding inhibitory activities against pancreatic lipase validated our strategy. As shown in Fig. 4b, the long chain of compound **A1** occupies subsite 2 very well, which presented an inhibitory activity of 0.083 μM. Subsequently, derivatives containing two long carbon chains that could simultaneously interact with subsites 2 and 3 were synthesized. The docking conformation of derivative B1 in the catalytic site of pancreatic lipase indicated that the two chains could properly interact with subsites 2 and 3 (Fig. 4c), with an IC_50_ value of 0.030 μM. Considering that subsite 2 is partially charged by the residues Asp79, Glu83 and Arg256, two long chain *N*,*N*-dialkyl amide derivatives were designed and synthesized to hydrophilically interact with the lipase. Finally, we obtained thirteen amide derivatives, and the most active derivative is shown in Fig. 4d, exhibiting an IC_50_ value of 14 nM. Using this sequential approach, we optimized the structure of vibralactone and increased the inhibitory activity from 47.26 μM to 14 nM.

## Conclusion

In summary, three series of 104 vibralactone-based analogs were designed by altering the length and functionality of the chain linking the 3-position of the vibralactone moiety. All of the synthesized compounds were evaluated for their inhibitory activities against pancreatic lipase in vitro. Compound **C1** appeared to be the most potent inhibitor of pancreatic lipase activity, with an IC_50_ value of 14 nM, which is more than 3000-fold higher than that of vibralactone. Compound **C1** was selected for further evaluation in vivo. The effect of compound **C1** on obesity was investigated using HFD-induced C57BL/6 J obese mice. Compound **C1** was administered at a dose of 100 mg/kg for 33 days. The antiobesity activity was evaluated by measuring the body weight, epididymal white adipose tissue and metabolic plasma parameters. From day 6 to day 33, the body weight of the compound **C1**-treated group was significantly low compared with the HFD-treated group. The metabolic parameters that increased in the HFD group decreased in the compound **C1**-treated group. Particularly, the increased triglyceride levels were significantly reduced in the compound **C1**-treated group. These results indicate that treatment with compound **C1** decreased HFD-induced obesity, primarily through the improvement of metabolic parameters, such as triglyceride and cholesterol levels. Therefore, compound **C1**, as a potent pancreatic lipase inhibitor, demonstrates potential benefits in the regulation of obesity. Although the investigated compounds were less potent than orlistat and no data was reported showing that they are safer than orlistat at moment, but this provides a fact that this type of compounds can be optimized and the possibility for further optimization to find better antiobesity agents.

## Experimental Protocols

### Materials Used for Chemical and Biological Experiments

All of the chemicals and reagents that were commercially available were purchased from Sigma-Aldrich and Acros and were used without further purification. All of the solvents were purified and dried using standard techniques and were distilled prior to use. All of the reactions were performed under a nitrogen atmosphere using oven-baked glassware unless otherwise noted. Flash chromatography was performed using mesh silica gel (200–300 mesh). Analytical thin-layer chromatography (TLC) on glass-backed silica gel GF 254 plates was used to monitor the reactions. Yields refer to chromatographically and spectroscopically homogeneous materials. NMR spectra were acquired on a Bruker DRX-400 or DRX-500 (Bruker BioSpin GmbH, Rheinstetten, Germany) spectrometer using deuterated chloroform signals (δ_H_ 7.26 ppm, δ_C_ 77.0 ppm) as the internal standard. EIMS (including HREIMS) spectra were measured on Finnigan MAT 90 (Thermo Fisher Scientific Inc., Waltham, MA, United States) and API QSTAR Pulsar i (MDS Sciex, Concord, Ontario, Canada) mass spectrometers, respectively. Preparative HPLC was performed using an Agilent 1100 Series HPLC system (ZORBAX SB-**C18** column, 5 μm, 9.4 × 150 mm).

### Vibralactone

The culture broth was filtered to remove the mycelium. The filtrate (500 L) was then successively extracted twice with ethyl acetate. The crude extract (150 g) was chromatographed on silica gel (200–300 mesh) and eluted with a gradient of petroleum ether/acetone to yield the vibralactone (10.5 g).

### General Procedure to Synthesize Compounds **A1**–**A54**

To a stirring solution of the appropriate carboxyl acids (0.12 mmol) in CH_2_Cl_2_ (2 mL) at 0 °C, thionyl chloride (0.6 mmol) was added dropwise. The reaction was monitored by TLC, and following the complete reaction of the starting material, the reaction mixture was concentrated to yield a brown–yellow oil. To a solution of vibralactone (0.1 mmol) in dichloromethane (2 mL) at 0 °C was added Et_3_N (0.2 mmol) and the corresponding acyl chloride dissolved in dichloromethane (2 mL). The reaction mixture was stirred at room temperature overnight. A saturated NH_4_Cl solution was added to quench the reaction, and the mixture was extracted with CH_2_Cl_2_ (3 × 10 mL). The combined organic layers were dried over MgSO_4_ and concentrated *in vacuo*. The products were purified using flash chromatography on silica gel.

#### ((1R,5S)-1-(3-methylbut-2-en-1-yl)-7-oxo-6-oxabicyclo[3.2.0]hept-2-en-3-yl)methyl-6-phenylhexanoate (**A1**)

Yield: 84 %. HR-EI-MS *m/z*: 382.2146 [M]^+^ (Calcd. for C_24_H_30_O_4_: 382.2144). ^1^H NMR (500 MHz, CDCl_3_) δ (ppm): 7.28 (2H, m), 7.18 (3H, m), 5.63 (1H, s), 5.13 (1H, m), 4.78 (1H, m), 4.65 (2H, s), 2.74 (2H, s), 2.61 (3H, t, *J* = 7.5 Hz), 2.41 (1H, m), 2.34 (2H, t, *J* = 7.5 Hz), 1.73 (3H, s), 1.67 (4H, m), 1.63 (3H, s), 1.37 (2H, m). ^13^C NMR (100 MHz, CDCl_3_) δ (ppm): 173.1, 172.3, 142.4, 141.5, 136.1, 128.4, 128.3, 125.7, 125.1, 117.2, 78.2, 75.4, 61.8, 37.7, 35.7, 34.0, 31.0, 28.7, 27.6, 25.7, 24.8, 18.0.

#### ((1R,5S)-1-(3-methylbut-2-en-1-yl)-7-oxo-6-oxabicyclo[3.2.0]hept-2-en-3-yl)methyl-3-(3-phenoxyphenyl)propanoate (**A2**)

Yield: 81 %. HR-EI-MS *m/z*: 432.1927 [M]^+^ (Calcd. for C_27_H_28_O_5_: 432.1937). ^1^H NMR (400 MHz, CDCl_3_) δ (ppm): 7.34 (2H, t, *J* = 7.5 Hz), 7.25 (1H, t, *J* = 7.5 Hz), 7.10 (1H, t, *J* = 7.4 Hz), 7.00 (2H, d, *J* = 8.3 Hz), 6.94 (1H, d, *J* = 7.5 Hz), 6.85 (2H, d, *J* = 6.9 Hz), 5.57 (1H, s), 5.10 (1H, t, *J* = 7.3 Hz), 4.77 (1H, s), 4.65 (2H, s), 2.94 (2H, t, *J* = 7.6 Hz), 2.67 (5H, m), 2.40 (1H, m), 1.72 (3H, s), 1.63 (3H, s). ^13^C NMR (100 MHz, CDCl_3_) δ (ppm): 172.4, 172.1, 157.4, 157.0, 142.2, 141.2, 136.2, 129.8, 129.7, 125.2, 123.3, 123.1, 118.9, 118.6, 117.1, 116.7, 78.1, 75.4, 62.0, 37.6, 35.4, 30.7, 27.5, 25.8, 18.0.

#### ((1R,5S)-1-(3-methylbut-2-en-1-yl)-7-oxo-6-oxabicyclo[3.2.0]hept-2-en-3-yl)methyltetradecanoate (**A3**)

Yield: 75 %. ESI-MS: 418 [M]^+^. ^1^H NMR (500 MHz, CDCl_3_) δ (ppm): 5.63 (1H, s), 5.11 (1H, m), 4.78 (1H, t, *J* = 2.9 Hz), 4.67 (2H, s), 2.75 (2H, s), 2.60 (1H, m), 2.41 (1H, m), 2.34 (2H, t, *J* = 7.6 Hz), 1.72 (3H, s), 1.63 (3H, s), 1.29 (22H, m), 0.87 (3H, t, *J* = 6.7 Hz). ^13^C NMR (125 MHz, CDCl_3_) δ (ppm): 173.3, 172.4, 141.5, 136.2, 125.0, 117.1, 78.1, 75.4, 61.7, 37.7, 34.1, 31.9, 29.7, 29.6, 29.5, 29.4, 29.3, 29.2, 29.1, 29.0, 27.5, 25.8, 24.9, 22.7, 18.0, 14.1.

#### ((1R,5S)-1-(3-methylbut-2-en-1-yl)-7-oxo-6-oxabicyclo[3.2.0]hept-2-en-3-yl)methyl-6-(4-fluorophenyl)hexanoate (**A4**)

Yield: 88 %. HR-EI-MS *m/z*: 400.2044 [M]^+^ (Calcd. for C_24_H_29_O_4_F: 400.2050). ^1^H NMR (400 MHz, CDCl_3_) δ (ppm): 7.11 (2H, m), 6.95 (2H, m), 5.63 (1H, s), 5.12 (1H, brs), 4.79 (1H, s), 4.65 (1H, s), 2.74 (2H, s), 2.60 (3H, m), 2.41 (1H, m), 2.34 (2H, t, *J* = 7.5 Hz), 1.72 (3H, s), 1.63 (9H, m), 1.35 (2H, m). ^13^C NMR (100 MHz, CDCl_3_) δ (ppm): 173.1, 172.4, 162.3, 159.9, 141.4, 137.9, 136.2, 129.6, 125.0, 117.1, 115.1, 114.8, 78.2, 75.3, 61.8, 37.7, 34.8, 34.0, 31.2, 28.6, 27.5, 25.8, 24.7, 18.0.

#### (Z)-((1R,5S)-1-(3-methylbut-2-en-1-yl)-7-oxo-6-oxabicyclo[3.2.0]hept-2-en-3-yl) methyldodec-5-enoate (**A5)**

Yield: 86 %. HR-ESI-MS *m/z*: 411.2513 [M + Na]^+^ (Calcd. for C_24_H_36_O_4_Na: 411.2511). ^1^H NMR (400 MHz, CDCl_3_) δ (ppm): 5.63 (1H, s), 5.34 (2H, m), 5.11 (1H, t, *J* = 6.9 Hz), 4.79 (1H, s), 4.66 (2H, s), 2.75 (2H, s), 2.61 (1H, m), 2.42 (1H, m), 2.35 (2H, t, *J* = 7.5 Hz), 2.04 (4H, m), 1.72 (3H, s), 1.63 (3H, s), 1.28 (10H, m), 0.87 (3H, m). ^13^C NMR (100 MHz, CDCl_3_) δ (ppm): 173.1, 172.4, 141.5, 136.2, 131.3, 128.1, 125.0, 117.1, 78.1, 75.4, 61.8, 37.7, 33.5, 31.7, 31.6, 29.6, 29.4, 29.0, 27.5, 27.2, 26.5, 25.8, 24.8, 22.6, 18.0, 14.1.

#### ((1R,5S)-1-(3-methylbut-2-en-1-yl)-7-oxo-6-oxabicyclo[3.2.0]hept-2-en-3-yl)methyl-3-phenylpropanoate (**A6**)

Yield: 85 %. HR-EI-MS *m/z*: 340.1657 [M]^+^ (Calcd. for C_21_H_24_O_4_: 340.1675). ^1^H NMR (400 MHz, CDCl_3_) δ (ppm): 7.30 (2H, m), 7.21 (3H, m), 5.54 (1H, s), 5.10 (1H, m), 4.75 (1H, m), 4.65 (2H, s), 2.97 (2H, t, *J* = 7.6 Hz), 2.67 (4H, m), 2.60 (1H, m), 2.42 (1H, m), 2.21 (1H, m), 1.71 (3H, s), 1.64 (3H, s). ^13^C NMR (100 MHz, CDCl_3_) δ (ppm): 172.4, 172.3, 141.3, 140.2, 136.2, 128.5, 128.3, 126.3, 125.1, 117.1, 78.1, 75.4, 62.0, 37.6, 35.6, 30.9, 27.5, 25.8, 18.0.

#### ((1R,5S)-1-(3-methylbut-2-en-1-yl)-7-oxo-6-oxabicyclo[3.2.0]hept-2-en-3-yl)methyl-5-(thiophen-2-yl)pentanoate (**A7**)

Yield: 87 %. HR-EI-MS *m/z*: 374.1557 [M]^+^ (Calcd. for C_21_H_26_O_4_S: 374.1552). ^1^H NMR (400 MHz, CDCl_3_) δ (ppm): 7.11 (1H, d, *J* = 4.8 Hz), 6.91 (1H, q, *J* = 3.6 Hz), 6.78 (1H, s), 5.63 (1H, s), 5.11 (1H, m), 4.78 (1H, s), 4.66 (2H, s), 2.86 (2H, s), 2.74 (2H, s), 2.60 (1H, m), 2.43 (3H, m), 1.72 (3H, s), 1.63 (3H, s). ^13^C NMR (100 MHz, CDCl_3_) δ (ppm): 172.9, 144.7, 141.4, 136.2, 126.7, 125.1, 124.2, 123.0, 117.1, 78.1, 75.4, 61.8, 37.7, 33.7, 31.1, 29.5, 27.5, 25.8, 24.3, 18.0.

#### ((1R,5S)-1-(3-methylbut-2-en-1-yl)-7-oxo-6-oxabicyclo[3.2.0]hept-2-en-3-yl)methyl-4-(4-methoxyphenyl)-4-methylpentanoate (**A8**)

Yield: 81 %. HR-EI-MS *m/z*: 412.2253 [M]^+^ (Calcd. for C_25_H_32_O_5_: 412.2250). ^1^H NMR (400 MHz, CDCl_3_) δ (ppm): 7.24 (2H, d, *J* = 8.8 Hz), 6.85 (2H, d, *J* = 8.8 Hz), 5.58 (1H, s), 5.10 (1H, m), 4.77 (1H, m), 4.61 (2H, s), 3.79 (3H, s), 2.71 (2H, s), 2.61 (1H, m), 2.42 (1H, m), 2.09 (2H, m), 1.95 (2H, m), 1.72 (3H, s), 1.63 (3H, s), 1.30 (6H, s). ^13^C NMR (100 MHz, CDCl_3_) δ (ppm): 173.5, 172.4, 150.5, 141.4, 139.8, 136.2, 126.8, 124.9, 117.1, 113.5, 78.1, 75.3, 61.8, 55.2, 39.0, 37.7, 36.7, 30.0, 28.9, 27.5, 25.8, 18.0.

#### (E)-((1R,5S)-1-(3-methylbut-2-en-1-yl)-7-oxo-6-oxabicyclo[3.2.0]hept-2-en-3-yl)methyldodec-2-enoate (**A9**)

Yield: 85 %. HR-EI-MS *m/z*: 388.2609 [M]^+^ (Calcd. for C_24_H_36_O_4_: 388.2614). ^1^H NMR (400 MHz, CDCl_3_) δ (ppm): 5.63 (1H, s), 5.53 (2H, m), 5.11 (1H, t, *J* = 7.3 Hz), 4.78 (1H, t, *J* = 2.9 Hz), 4.68 (2H, s), 3.12 (1H, d, *J* = 6.6 Hz), 3.06 (1H, d, *J* = 6.6 Hz), 2.74 (2H, s), 2.61 (1H, m), 2.41 (1H, m), 2.04 (2H, m), 1.72 (3H, s), 1.63 (3H, s), 1.27 (12H, m), 0.87 (3H, t, *J* = 6.4 Hz). ^13^C NMR(100 MHz, CDCl_3_) δ (ppm): 172.4, 171.5, 141.3, 136.2, 135.4, 133.9, 125.0, 121.0, 120.2, 117.1, 78.1, 75.3, 62.0, 37.9, 37.7, 32.8, 32.5, 31.8, 29.5, 29.4, 29.3, 29.2, 29.1, 27.5, 27.4, 25.8, 22.6, 18.0, 14.1.

#### 1-(((1R,5S)-1-(3-methylbut-2-en-1-yl)-7-oxo-6-oxabicyclo[3.2.0]hept-2-en-3-yl)methyl)-12-(((1S,5R)-1-(3-methylbut-2-en-1-yl)-7-oxo-6-oxabicyclo[3.2.0]hept-2-en-3-yl)methyl)dodecanedioate (**A10**)

Yield: 79 %. HR-EI-MS *m/z*: 610.3502 [M]^+^ (Calcd. for C_36_H_50_O_8_: 610.3506). ^1^H NMR (400 MHz, CDCl_3_) δ (ppm): 5.63 (1H, s), 5.11 (1H, t, *J* = 7.3 Hz), 4.78 (1H, t, *J* = 2.7 Hz), 4.65 (2H, s), 2.75 (2H, s), 2.60 (1H, m), 2.42 (1H, m), 2.33 (2H, t, *J* = 7.5 Hz), 1.72 (3H, s), 1.63 (3H, s), 1.29 (6H, s). ^13^C NMR (100 MHz, CDCl_3_) δ (ppm): 173.2, 172.4, 141.5, 136.2, 125.0, 117.1, 78.2, 75.4, 61.7, 37.7, 34.1, 29.3, 29.2, 29.1, 27.5, 25.8, 24.9, 18.0.

#### ((1R,5S)-1-(3-methylbut-2-en-1-yl)-7-oxo-6-oxabicyclo[3.2.0]hept-2-en-3-yl)methyl-2-((1S,2R,4R)-bicyclo[2.2.1]heptan-2-yl)acetate (**A11**)

Yield: 84 %. HR-ESI-MS *m/z*: 367.1891[M + Na]^+^ (Calcd. for C_21_H_28_O_4_Na: 367.1885). ^1^H NMR (400 MHz, CDCl_3_) δ (ppm): 5.63 (1H, s), 5.11 (1H, t, *J* = 7.3 Hz), 4.79 (2H, t, *J* = 3.0 Hz), 4.65 (2H, s), 2.75 (2H, s), 2.60 (1H, m), 2.42 (1H, m), 2.35-2.14 (3H, m), 1.98 (1H, s), 1.87 (1H. m), 1.72 (3H, s), 1.64 (3H, s), 1.57 (2H, s), 1.50 (2H, m), 1.31-1.04 (4H, m). ^13^C NMR (100 MHz, CDCl_3_) δ (ppm): 172.6, 141.5, 136.2, 125.0, 117.1, 78.2, 75.3, 61.7, 41.1, 41.0, 38.4, 37.8, 37.7, 36.7, 35.2, 29.7, 28.5, 27.5, 25.8, 18.0.

#### ((1R,5S)-1-(3-methylbut-2-en-1-yl)-7-oxo-6-oxabicyclo[3.2.0]hept-2-en-3-yl)methyl-5-(4-methoxyphenyl)pentanoate (**A12**)

Yield: 75 %. HR-EI-MS *m/z*: 398.2087 [M]^+^ (Calcd. for C_24_H_30_O_5_: 398.2093). ^1^H NMR (400 MHz, CDCl_3_) δ (ppm): 7.10 (2H, d, *J* = 8.6 Hz), 6.82 (2H, d, *J* = 8.6 Hz), 5.62 (1H, s), 5.11 (1H, m), 4.78 (1H, m), 4.67 (2H, s), 3.79 (3H, s), 2.74 (2H, s), 2.60 (3H, m), 2.41 (3H, m), 1.72 (3H, s), 1.67 (3H, s), 1.65 (4H, m). ^13^C NMR (100 MHz, CDCl_3_) δ (ppm): 173.1, 172.5, 157.7, 141.4, 136.2, 134.0, 129.2, 125.0, 117.1, 113.7, 78.1, 75.4, 61.8, 55.2, 37.7, 34.6, 33.9, 31.1, 27.5, 25.8, 24.4, 18.0.

#### 1-(((1R,5S)-1-(3-methylbut-2-en-1-yl)-7-oxo-6-oxabicyclo[3.2.0]hept-2-en-3-yl)methyl)-10-(((1S,5R)-1-(3-methylbut-2-en-1-yl)-7-oxo-6-oxabicyclo[3.2.0]hept-2-en-3-yl)methyl)decanedioate (**A13**)

Yield: 87 %. HR-EI-MS *m/z*: 582.3207 [M]^+^ (Calcd. for C_34_H_46_O_8_: 582.3193). ^1^H NMR (400 MHz, CDCl_3_) δ (ppm): 5.63 (1H, s), 5.11 (1H, m), 4.80 (1H, t, *J* = 2.7 Hz), 4.66 (2H, s), 2.75 (2H, s), 2.60 (1H, m), 2.42 (1H, m), 2.34 (2H, t, *J* = 7.5 Hz), 1.72 (3H, s), 1.63 (6H, s), 1.30 (4H, s). ^13^C NMR (100 MHz, CDCl_3_) δ (ppm): 173.2, 172.5, 141.5, 136.2, 126.5, 117.1, 78.2, 75.4, 61.8, 37.7, 34.0, 29.0, 27.5, 25.8, 24.8, 18.0.

#### ((1R,5S)-1-(3-methylbut-2-en-1-yl)-7-oxo-6-oxabicyclo[3.2.0]hept-2-en-3-yl)methyl-5-phenylpentanoate (**A14**)

Yield: 85 %. HR-EI-MS *m/z*: 368.1978 [M]^+^ (Calcd. for C_23_H_28_O_4_: 368.1988). ^1^H NMR (400 MHz, CDCl_3_) δ (ppm): 7.29 (2H, m), 7.19 (3H, m), 5.63 (1H, s), 5.11 (1H, m), 4.77 (1H, m), 4.65 (2H, s), 2.74 (2H, s), 2.66 (3H, m), 2.42 (3H, m), 1.73 (3H, s), 1.68 (4H, m), 1.63 (3H, s). ^13^C NMR (100 MHz, CDCl_3_) δ (ppm): 173.0, 172.4, 142.0, 141.4, 136.2, 128.4, 128.3, 125.8, 125.0, 117.0, 78.1, 75.4, 61.8, 37.7, 35.5, 33.9, 30.8, 27.5, 25.8, 24.5, 18.0.

#### ((1R,5S)-1-(3-methylbut-2-en-1-yl)-7-oxo-6-oxabicyclo[3.2.0]hept-2-en-3-yl)methyl-4-phenylbutanoate (**A15**)

Yield: 80 %. HR-EI-MS *m/z*: 354.1765 [M]^+^ (Calcd. for C_22_H_26_O_4_: 354.1831). ^1^H NMR (400 MHz, CDCl_3_) δ (ppm): 7.30 (2H, m), 7.21 (3H, m), 5.63 (1H, s), 5.11 (1H, m), 4.79 (1H, m), 4.69 (2H, s), 2.75 (2H, s), 2.66 (2H, t, *J* = 7.5 Hz), 2.60 (1H, m), 2.42 (1H, m), 2.37 (2H, t, *J* = 7.5 Hz), 1.98 (2H, t, *J* = 7.5 Hz), 1.71 (3H, s), 1.63 (3H, s). ^13^C NMR (100 MHz, CDCl_3_) δ (ppm): 172.9, 172.4, 141.4, 141.1, 136.2, 128.4, 128.3, 126.0, 125.1, 117.1, 78.1, 75.4, 61.8, 37.8, 35.1, 33.3, 27.5, 26.4, 25.8, 18.0.

#### ((1R,5S)-1-(3-methylbut-2-en-1-yl)-7-oxo-6-oxabicyclo[3.2.0]hept-2-en-3-yl)methyldodecanoate (**A16**)

Yield: 81 %. HR-EI-MS *m/z*: 390.1830 [M]^+^ (Calcd. for C_24_H_26_O_4_: 390.1831). ^1^H NMR (400 MHz, CDCl_3_) δ (ppm): 5.63 (1H, s), 5.11 (1H, m), 4.78 (1H, t, *J* = 2.9 Hz), 4.65 (2H, s), 2.74 (2H, s), 2.60 (1H, m), 2.41 (1H, m), 2.34 (2H, t, *J* = 7.6 Hz), 1.72 (3H, s), 1.63 (3H, s), 1.29 (18H, m), 0.87 (3H, t, *J* = 6.7 Hz). ^13^C NMR (125 MHz, CDCl_3_) δ (ppm): 173.3, 172.4, 141.5, 136.2, 125.0, 117.1, 78.1, 75.4, 61.7, 37.7, 34.1, 31.9, 29.6, 29.5, 29.4, 29.3, 29.2, 29.1, 29.0, 28.8, 27.5, 25.8, 24.9, 24.2, 22.7, 18.0, 14.1.

#### ((1R,5S)-1-(3-methylbut-2-en-1-yl)-7-oxo-6-oxabicyclo[3.2.0]hept-2-en-3-yl)methyl-3-(thiophen-2-yl)propanoate (**A17**)

Yield: 86 %. HR-EI-MS *m/z*: 346.1238 [M]^+^ (Calcd. for C_19_H_22_O_4_S: 346.1239). ^1^H NMR (500 MHz, CDCl_3_) δ (ppm): 7.13 (1H, t, *J* = 0.9 Hz), 6.90 (1H, q, *J* = 3.5 Hz), 6.82 (1H, d, *J* = 3.5 Hz), 5.57 (1H, s), 5.10 (1H, m), 4.76 (1H, t, *J* = 3.5 Hz), 4.67 (2H, s), 3.17 (2H, t, *J* = 7.4 Hz), 2.73 (2H, t, *J* = 7.4 Hz), 2.69 (2H, s), 2.60 (1H, m), 2.40 (1H, m), 1.72 (3H, s), 1.63 (3H, s). ^13^C NMR (100 MHz, CDCl_3_) δ (ppm): 172.4, 171.8, 142.7, 141.2, 136.2, 126.9, 125.2, 124.8, 123.6, 117.1, 78.1, 75.4, 62.1, 37.7, 35.9, 27.5, 25.8, 25.1, 18.0.

#### ((1R,5S)-1-(3-methylbut-2-en-1-yl)-7-oxo-6-oxabicyclo[3.2.0]hept-2-en-3-yl)methyl palmitate (**A18**)

Yield: 89 %. ESI-MS: 446 [M]^+^. ^1^H NMR (400 MHz, CDCl_3_) δ (ppm): 5.63 (1H, s), 5.11 (1H, m), 4.78 (1H, t, *J* = 2.9 Hz), 4.60 (2H, s), 2.75 (2H, s), 2.60 (1H, m), 2.41 (1H, m), 2.34 (2H, t, *J* = 7.6 Hz), 1.75 (3H, s), 1.65 (3H, s), 1.29 (26H, m), 0.87 (3H, t, *J* = 6.7 Hz). ^13^C NMR (125 MHz, CDCl_3_) δ (ppm): 173.3, 172.4, 141.5, 136.2, 125.0, 117.1, 78.1, 75.4, 61.7, 37.7, 34.1, 31.9, 29.7, 29.6, 29.5, 29.4, 29.3, 29.2, 29.1, 29.0, 28.8, 27.5, 24.2, 22.7, 18.0, 14.1.

#### ((1R,5S)-1-(3-methylbut-2-en-1-yl)-7-oxo-6-oxabicyclo[3.2.0]hept-2-en-3-yl)methyl-(((1S,5R)-1-(3-methylbut-2-en-1-yl)-7-oxo-6-oxabicyclo[3.2.0]hept-2-en-3-yl)methyl) oxalate (**A19**)

Yield: 80 %. ESI-MS: 493 [M + Na]^+^. ^1^H NMR (400 MHz, CD_3_COCD_3_) δ (ppm): 5.56 (1H, s), 5.17 (1H, m), 4.87 (1H, d, *J* = 4.6 Hz), 4.15 (2H, m), 4.08 (1H, t, *J* = 4.3 Hz), 2.60 (3H, m), 2.42 (1H, m), 1.69 (3H, s), 1.64 (3H, s). ^13^C NMR (100 MHz, CDCl_3_) δ (ppm): 173.6, 149.1, 136.0, 122.1, 119.0, 79.3, 76.0, 61.2, 38.0, 28.3, 26.0, 18.0.

#### 1-(((1R,5S)-1-(3-methylbut-2-en-1-yl)-7-oxo-6-oxabicyclo[3.2.0]hept-2-en-3-yl)methyl)-8-(((1S,5R)-1-(3-methylbut-2-en-1-yl)-7-oxo-6-oxabicyclo[3.2.0]hept-2-en-3-yl)methyl)octanedioate (**A20**)

Yield: 85 %. HR-EI-MS *m/z*: 554.2889 [M]^+^ (Calcd. for C_32_H_42_O_8_: 554.2880). ^1^H NMR (400 MHz, CDCl_3_) δ (ppm): 5.63 (1H, s), 5.11 (1H, t, *J* = 7.3 Hz), 4.78 (1H, t, *J* = 2.7 Hz), 4.65 (2H, s), 2.75 (2H, s), 2.60 (1H, m), 2.42 (1H, m), 2.33 (2H, t, *J* = 7.5 Hz), 1.72 (3H, s), 1.63 (3H, s), 1.35 (4H, s). ^13^C NMR (100 MHz, CDCl_3_) δ (ppm): 173.0, 172.4, 141.4, 136.2, 125.1, 117.1, 78.2, 75.4, 61.7, 37.7, 33.9, 28.7, 27.5, 25.8, 24.6, 18.0.

#### (2E,4E)-((1R,5S)-1-(3-methylbut-2-en-1-yl)-7-oxo-6-oxabicyclo[3.2.0]hept-2-en-3-yl)methylhexa-2,4-dienoate (**A21**)

Yield: 89 %. HR-ESI-MS *m/z*: 325.1415 [M + Na]^+^ (Calcd. for C_18_H_22_O_4_Na: 325.1415). ^1^H NMR (400 MHz, CDCl_3_) δ (ppm): 6.33 (1H, m), 6.16 (1H, m), 5.79 (1H, m), 5.64 (1H, s), 5.14 (3H, m), 4.79 (1H, t, *J* = 3.0 Hz), 4.68 (2H, s), 3.17 (2H, d, *J* = 7.1 Hz), 2.75 (2H, s), 2.60 (1H, m), 2.42 (1H, m), 1.72 (3H, s), 1.63 (3H, s). ^13^C NMR (100 MHz, CDCl_3_) δ (ppm): 172.4, 170.9, 141.1, 136.2, 136.1, 134.7, 125.2, 124.9, 117.3, 117.0, 78.1, 75.4, 62.2, 37.7, 37.6, 27.5, 25.8, 18.0.

#### ((1R,5S)-1-(3-methylbut-2-en-1-yl)-7-oxo-6-oxabicyclo[3.2.0]hept-2-en-3-yl)methylundecanoate (**A22**)

Yield: 85 %. HR-ESI-MS *m/z*: 399.2513 [M + Na]^+^ (Calcd. for C_23_H_36_O_4_Na: 399.2511). ^1^H NMR (400 MHz, CDCl_3_) δ (ppm): 5.63 (1H, s), 5.11 (1H, m), 4.78 (1H, t, *J* = 3.0 Hz), 4.66 (2H, s), 2.75 (2H, s), 2.62 (1H, m), 2.41 (1H, m), 2.34 (2H, t, *J* = 7.5 Hz), 1.72 (3H, s), 1.64 (3H, s), 1.29 (16H, m), 0.87 (3H, t, *J* = 6.5 Hz). ^13^C NMR (100 MHz, CDCl_3_) δ (ppm): 173.3, 172.4, 141.5, 136.2, 125.0, 117.1, 78.1, 75.4, 61.7, 37.7, 34.1, 31.9, 29.5, 29.4, 29.3, 29.2, 29.1, 25.8, 24.9, 22.7, 18.0, 14.1.

#### ((1R,5S)-1-(3-methylbut-2-en-1-yl)-7-oxo-6-oxabicyclo[3.2.0]hept-2-en-3-yl)methylundec-10-enoate (**A23**)

Yield: 80 %. HR-ESI-MS *m/z*: 397.2347 [M + Na]^+^ (Calcd. for C_23_H_34_O_4_Na: 397.2354). ^1^H NMR (400 MHz, CDCl_3_) δ (ppm): 5.80 (1H, m), 5.63 (1H, s), 5.11 (1H, m), 4.96 (2H, m), 4.79 (1H, t, *J* = 3.0 Hz), 4.66 (2H, s), 2.75 (2H, s), 2.62 (1H, m), 2.41 (1H, m), 2.34 (2H, t, *J* = 7.5 Hz), 2.04 (2H, q, *J* = 6.8 Hz), 1.72 (3H, s), 1.64 (3H, s), 1.60 (2H, m), 1.29 (10H, m). ^13^C NMR (100 MHz, CDCl_3_) δ (ppm): 173.3, 172.4, 141.5, 139.1, 136.2, 125.0, 117.1, 114.1, 78.1, 75.4, 61.7, 37.7, 34.1, 33.7, 29.2, 29.1, 29.0, 28.8, 27.5, 25.8, 24.9, 18.0.

#### ((1R,5S)-1-(3-methylbut-2-en-1-yl)-7-oxo-6-oxabicyclo[3.2.0]hept-2-en-3-yl)methyl-(((1S,5R)-1-(3-methylbut-2-en-1-yl)-7-oxo-6-oxabicyclo[3.2.0]hept-2-en-3-yl)methyl) succinate (**A24**)

Yield: 83 %. HR-EI-MS *m/z*: 498.2262 [M] (Calcd. for C_28_H_34_O_8_: 498.2254). ^1^H NMR (500 MHz, CDCl_3_) δ (ppm): 5.68 (1H, s), 5.13 (1H, t, *J* = 6.3 Hz), 4.81 (1H, t, *J* = 2.8 Hz), 4.71 (2H, s), 2.78 (2H, s), 2.71 (2H, s), 2.60 (1H, m), 2.42 (1H, m), 1.75 (3H, s), 1.66 (3H, s). ^13^C NMR (100 MHz, CDCl_3_) δ (ppm): 172.4, 171.7, 141.1, 136.2, 125.3, 117.0, 78.1, 75.4, 62.0, 37.7, 28.8, 27.5, 25.8, 18.0.

#### (9Z,12Z)-((1R,5S)-1-(3-methylbut-2-en-1-yl)-7-oxo-6-oxabicyclo[3.2.0]hept-2-en-3-yl)methyl octadeca-9,12-dienoate (**A25**)

Yield: 80 %. HR-ESI-MS *m/z*: 493.3297 [M + Na]^+^ (Calcd. for C_30_H_46_O_4_Na: 493.3293). ^1^H NMR (400 MHz, CDCl_3_) δ (ppm): 5.63 (1H, s), 5.35 (4H, m), 5.11 (1H, m), 4.78 (1H, t, *J* = 3.0 Hz), 4.66 (2H, s), 2.76 (4H, m), 2.60 (1H, m), 2.41 (1H, m), 2.33 (2H, t, *J* = 7.5 Hz), 2.05 (4H, m), 1.72 (3H, s), 1.63 (3H, s), 1.32 (16H, m), 0.88 (3H, t, *J* = 6.7 Hz). ^13^C NMR (100 MHz, CDCl_3_) δ (ppm): 173.2, 172.4, 141.5, 136.2, 130.2, 130.0, 128.0, 127.9, 125.0, 117.1, 78.2, 75.3, 61.8, 37.7, 34.1, 31.5, 29.6, 29.3, 29.1, 29.0, 27.5, 27.2, 27.1, 25.8, 25.6, 24.9, 22.6, 18.0, 14.1.

#### ((1R,5S)-1-(3-methylbut-2-en-1-yl)-7-oxo-6-oxabicyclo[3.2.0]hept-2-en-3-yl)methyltridecanoate (**A26**)

Yield: 83 %. HR-ESI-MS *m/z*: 427.2824 [M + Na]^+^ (Calcd. for C_25_H_40_O_4_Na: 427.2824). ^1^H NMR (400 MHz, CDCl_3_) δ (ppm): 5.63 (1H, s), 5.11 (1H, m), 4.78 (1H, t, *J* = 3.0 Hz), 4.66 (2H, s), 2.75 (2H, s), 2.60 (1H, m), 2.41 (1H, m), 2.34 (2H, t, *J* = 7.5 Hz), 1.72 (3H, s), 1.64 (3H, s), 1.25 (20H, m), 0.87 (3H, t, *J* = 6.4 Hz). ^13^C NMR (100 MHz, CDCl_3_) δ (ppm): 173.3, 172.4, 141.5, 136.2, 125.0, 117.1, 78.1, 75.4, 61.7, 37.7, 34.1, 31.9, 29.6, 29.5, 29.4, 29.3, 29.2, 29.1, 27.5, 25.8, 24.9, 22.6, 18.0, 14.1.

#### ((1R,5S)-1-(3-methylbut-2-en-1-yl)-7-oxo-6-oxabicyclo[3.2.0]hept-2-en-3-yl)methyl-(((1S,5R)-1-(3-methylbut-2-en-1-yl)-7-oxo-6-oxabicyclo[3.2.0]hept-2-en-3-yl)methyl) adipate (**A27**)

Yield: 85 %. HR-EI-MS *m/z*: 526.1998 [M]^+^ (Calcd. for C_30_H_38_O_8_: 526.2567). ^1^H NMR (400 MHz, CDCl_3_) δ (ppm): 5.67 (1H, s), 5.11 (1H, s), 4.78 (2H, m), 4.70 (1H, m), 2.78 (2H, m), 2.60 (1H, m), 2.42 (1H, m), 2.31 (2H, m), 2.15 (1H, m), 1.89 (1H, m), 1.72 (3H, s), 1.63 (3H, s). ^13^C NMR (100 MHz, CDCl_3_) δ (ppm): 172.4, 168.8, 140.9, 136.2, 125.5, 117.1, 78.2, 75.4, 62.7, 38.0, 37.6, 27.5, 27.3, 25.7, 20.9.

#### ((1R,5S)-1-(3-methylbut-2-en-1-yl)-7-oxo-6-oxabicyclo[3.2.0]hept-2-en-3-yl)methyl stearate (**A28**)

Yield: 86 %. ESI-MS: 474 [M]^+^. ^1^H NMR (500 MHz, CDCl_3_) δ (ppm): 5.63 (1H, s), 5.11 (1H, m), 4.78 (1H, s), 4.65 (2H, s), 2.75 (2H, s), 2.60 (1H, m), 2.41 (1H, m), 2.33 (2H, t, *J* = 7.5 Hz), 1.72 (3H, s), 1.63 (3H, s), 1.25 (30H, m), 0.87 (3H, t, *J* = 6.7 Hz). ^13^C NMR (125 MHz, CDCl_3_) δ (ppm): 173.3, 172.4, 141.5, 136.2, 125.0, 117.1, 78.1, 75.4, 61.7, 37.7, 34.1, 31.9, 29.7, 29.6, 29.5, 29.4, 29.3, 29.2, 29.1, 28.8, 27.5, 25.8, 24.9, 22.7, 18.0, 14.1.

#### ((1R,5S)-1-(3-methylbut-2-en-1-yl)-7-oxo-6-oxabicyclo[3.2.0]hept-2-en-3-yl)methyl-3-(furan-2-yl)propanoate (**A29**)

Yield: 85 %. HR-EI-MS *m/z*: 330.1470 [M]^+^ (Calcd. for C_19_H_22_O_5_: 330.1467). ^1^H NMR (500 MHz, CDCl_3_) δ (ppm): 7.31 (1H,s), 6.28 (1H, s), 6.03 (1H, s), 5.60 (1H, s), 5.10 (1H, t, *J* = 6.4 Hz), 4.77 (1H, t, *J* = 3.0 Hz), 4.68 (2H, s), 2.98 (2H, t, *J* = 7.5 Hz), 2.71 (3H, s), 2.59 (1H, m), 2.40 (1H, m), 1.72 (3H, s), 1.63 (3H, s). ^13^C NMR (100 MHz, CDCl_3_) δ (ppm): 172.4, 172.0, 153.8, 141.3, 136.2, 125.2, 117.1, 110.2, 105.4, 78.1, 75.4, 62.1, 37.7, 325, 27.5, 25.8, 23.4, 18.0.

#### (R)-((1R,5S)-1-(3-methylbut-2-en-1-yl)-7-oxo-6-oxabicyclo[3.2.0]hept-2-en-3-yl)methyl-1-formylpyrrolidine-2-carboxylate (**A30**)

Yield: 79 %. HR-EI-MS *m/z*: 333.1558 [M]^+^ (Calcd. for C_18_H_23_NO_5_: 333.1576). ^1^H NMR (500 MHz, CDCl_3_) δ (ppm): 8.29 (1H, s), 5.66 (1H, m), 5.10 (1H, m), 4.76 (2H, m), 4.46 (1H, m), 3.64 (2H, m), 2.74 (2H, m), 2.60 (1H, m), 2.40 (1H, m), 2.25 (2H, m), 2.00 (2H, m), 1.72 (3H, s), 1.63 (3H, s), 1.26 (2H, m). ^13^C NMR (100 MHz, CDCl_3_) δ (ppm): 171.2, 161.4, 160.7, 140.9, 136.2, 125.5, 117.1, 78.2, 75.4, 62.7, 56.4, 46.3, 37.7, 29.4, 27.5, 25.8, 22.8, 16.9.

#### (Z)-((1R,5S)-1-(3-methylbut-2-en-1-yl)-7-oxo-6-oxabicyclo[3.2.0]hept-2-en-3-yl)methylheptadec-10-enoate (**A31**)

Yield: 82 %. HR-ESI-MS *m/z*: 481.3290 [M + Na]^+^ (Calcd. for C_29_H_46_O_4_Na: 481.3293). ^1^H NMR (500 MHz, CDCl_3_) δ (ppm): 5.63 (1H, s), 5.34 (2H, m), 5.11 (1H, m), 4.78 (1H, t, *J* = 3.0 Hz), 4.66 (2H, s), 2.75 (2H, s), 2.60 (1H, m), 2.41 (1H, m), 2.33 (2H, t, *J* = 7.5 Hz), 2.00 (4H, s), 1.72 (3H, s), 1.63 (3H, s), 1.27 (20H, m), 0.88 (3H, t, *J* = 6.5 Hz). ^13^C NMR (125 MHz, CDCl_3_) δ (ppm): 173.2, 172.3, 141.6, 136.2, 123.0, 129.8, 125.1, 117.2, 78.2, 75.5, 61.8, 37.8, 34.1, 31.8, 29.7, 29.3, 29.2, 29.1, 29.0, 27.5, 27.2, 27.1, 25.7, 24.9, 22.6, 18.0, 14.0.

#### ((1R,5S)-1-(3-methylbut-2-en-1-yl)-7-oxo-6-oxabicyclo[3.2.0]hept-2-en-3-yl)methyl-7-phenylheptanoate (**A32**)

Yield: 85 %. HR-EI-MS *m/z*: 396.2308 [M]^+^ (Calcd. for C_25_H_32_O_4_: 396.2301). ^1^H NMR (400 MHz, CDCl_3_) δ (ppm): 7.28 (2H, m), 7.18 (3H, m), 5.63 (1H, s), 5.13 (1H, m), 4.79 (1H, m), 4.66 (2H, s), 2.75 (2H, s), 2.61 (3H, t, *J* = 7.5 Hz), 2.44 (1H, m), 2.35 (2H, t, *J* = 7.5 Hz), 1.73 (3H, s), 1.64 (7H, m), 1.37 (4H, m). ^13^C NMR (100 MHz, CDCl_3_) δ (ppm): 179.7, 173.2, 142.6, 141.5, 136.2, 128.4, 128.3, 125.6, 125.0, 117.0, 78.1, 75.4, 61.8, 37.7, 35.5, 33.9, 31.2, 28.8, 27.5, 25.8, 24.5, 18.0.

#### ((1R,5S)-1-(3-methylbut-2-en-1-yl)-7-oxo-6-oxabicyclo[3.2.0]hept-2-en-3-yl)methyl-4-acetamidobenzoate (**A33**)

Yield: 85 %. HR-EI-MS *m/z*: 369.1556 [M]^+^ (Calcd. for C_21_H_23_NO_5_: 369.1576). ^1^H NMR (400 MHz, CDCl_3_) δ (ppm):8.19 (1H, s), 7.97 (2H, d, *J* = 8.5 Hz), 7.63 (2H, d, *J* = 8.5 Hz), 5.69 (1H, s), 5.09 (1H, t, *J* = 7.2 Hz), 4.87 (2H, s), 4.83 (1H, s), 2.77 (2H, s), 2.60 (1H, m), 2.42 (1H, m), 2.18 (3H, s), 1.69 (3H, s), 1.61 (3H, s). ^13^C NMR (100 MHz, CDCl_3_) δ (ppm): 172.7, 168.9, 165.4, 142.7, 141.6, 136.2, 130.8, 124.9, 118.8, 116.9, 78.3, 75.4, 62.2, 37.8, 27.5, 25.7, 24.6, 17.9.

#### methyl (((1R,5S)-1-(3-methylbut-2-en-1-yl)-7-oxo-6-oxabicyclo[3.2.0]hept-2-en-3- yl)methyl) glutarate (**A34**)

Yield: 87 %. HR-EI-MS *m/z*: 336.1552 [M]^+^ (Calcd. for C_18_H_24_O_6_: 336.1573). ^1^H NMR (400 MHz, CDCl_3_) δ (ppm): 5.63 (1H, s), 5.11 (1H, m), 4.78 (1H, m), 4.66 (2H, s), 3.68 (3H, s), 2.74 (2H, s), 2.62 (1H, m), 2.40 (5H, m), 1.95 (2H, m), 1.71 (3H, s), 1.64 (3H, s). ^13^C NMR (100 MHz, CDCl_3_) δ (ppm): 173.2, 172.3, 141.3, 136.2, 125.2, 117.0, 78.1, 75.4, 62.0, 51.6, 37.7, 33.0, 32.9, 27.5, 25.7, 18.0.

#### methyl (((1R,5S)-1-(3-methylbut-2-en-1-yl)-7-oxo-6-oxabicyclo[3.2.0]hept-2-en-3- yl)methyl) malonate (**A35**)

Yield: 85 %. HR-ESI-MS *m/z*: 331.1152 [M + Na]^+^ (Calcd. for C_16_H_20_O_6_Na: 331.1157). ^1^H NMR (400 MHz, CDCl_3_) δ (ppm): 5.67 (1H, s), 5.11 (1H, m), 4.79 (1H, m), 4.74 (2H, s), 3.76 (3H, s), 3.44 (2H, s), 2.76 (2H, s), 2.62 (1H, m), 2.42 (1H, m), 1.72 (3H, s), 1.64 (3H, s). ^13^C NMR (100 MHz, CDCl_3_) δ (ppm): 172.3, 140.6, 136.3, 125.8, 117.0, 78.1, 75.4, 62.9, 52.6, 41.1, 37.6, 27.5, 25.8, 18.0.

#### (Z)-((1R,5S)-1-(3-methylbut-2-en-1-yl)-7-oxo-6-oxabicyclo[3.2.0]hept-2-en-3-yl)methyloctadec-6-enoate (**A36**)

Yield: 85 %. HR-ESI-MS *m/z*: 495.3443 [M + Na]^+^ (Calcd. for C_30_H_48_O_4_Na: 495.3450). ^1^H NMR (400 MHz, CDCl_3_) δ (ppm): 5.63 (1H, s), 5.34 (2H, m), 5.11 (1H, t, *J* = 7.3 Hz), 4.78 (1H, t, *J* = 3.0 Hz), 4.65 (2H, s), 2.75 (2H, s), 2.59 (1H, m), 2.43 (1H, m), 2.34 (2H, t, *J* = 7.5 Hz), 2.00 (4H, m), 1.72 (3H, s), 1.63 (3H, s), 1.26 (22H, m), 0.87 (3H, t, *J* = 6.4 Hz). ^13^C NMR (100 MHz, CDCl_3_) δ (ppm): 173.1, 172.4, 141.5, 136.2, 130.5, 128.9, 125.0, 117.1, 78.1, 75.4, 61.8, 37.7, 34.0, 31.9, 29.7, 29.6, 29.5, 29.3, 29.2, 29.1, 27.5, 27.2, 26.8, 25.8, 24.5, 22.7, 18.0, 14.1.

#### (S)-((1R,5S)-1-(3-methylbut-2-en-1-yl)-7-oxo-6-oxabicyclo[3.2.0]hept-2-en-3-yl)methyl-2-formamido-3-phenylpropanoate (**A37**)

Yield: 85 %. HR-EI-MS *m/z*: 383.1729 [M]^+^ (Calcd. for C_22_H_25_O_5_N: 383.1733). ^1^H NMR (600 MHz, CDCl_3_) δ (ppm): 8.17 (1H, s), 7.25 (3H, m), 7.10 (2H, d, *J* = 6.6 Hz), 6.02 (1H, d, *J* = 6.6 Hz), 5.53 (1H, s), 5.08 (1H, t, *J* = 7.2 Hz), 4.97 (1H, q, *J* = 6.6 Hz), 4.76 (1H, s), 4.72 (1H, q, *J* = 7.2 Hz), 3.12 (1H, d, *J* = 6.0 Hz), 2.65 (2H, s), 2.59 (1H, m), 2.41 (1H, m), 1.71 (3H, s), 1.62 (3H, s). ^13^C NMR (100 MHz, CDCl_3_) δ (ppm): 172.4, 171.0, 160.7, 140.4, 136.6, 135.4, 129.4, 129.3, 127.7, 126.1, 117.1, 78.3, 75.6, 63.3, 52.1, 38.1, 37.9, 27.7, 26.1, 18.0.

#### (S)-((1R,5S)-1-(3-methylbut-2-en-1-yl)-7-oxo-6-oxabicyclo[3.2.0]hept-2-en-3-yl)methyl-2-formamido-4-methylpentanoate (**A38**)

Yield: 80 %. HR-EI-MS *m/z*: 349.1911 [M]^+^ (Calcd. for C_19_H_27_O_5_N: 349.1889). ^1^H NMR (400 MHz, CDCl_3_) δ (ppm): 8.22 (1H, s), 6.01 (1H, d, *J* = 7.4 Hz), 5.67 (1H, s), 5.11 (1H, m), 4.79 (1H, m), 4.72 (3H, m), 2.77 (2H, s), 2.62 (1H, m), 2.42 (1H, m), 1.72 (3H, s), 1.68 (2H, m), 1.64 (3H, s), 1.25 (1H, m), 0.96 (6H, s). ^13^C NMR (100 MHz, CDCl_3_) δ (ppm): 172.1, 160.8, 140.5, 136.3, 126.1, 125.8, 117.0, 78.1, 75.4, 62.9, 49.3, 41.5, 37.7, 27.5, 25.8, 24.8, 22.7, 21.8, 18.0.

#### (Z)-((1R,5S)-1-(3-methylbut-2-en-1-yl)-7-oxo-6-oxabicyclo[3.2.0]hept-2-en-3-yl)methyloctadec-11-enoate (**A39**)

Yield: 80 %. HR-ESI-MS *m/z*: 495.3461 [M + Na]^+^ (Calcd. for C_30_H_48_O_4_Na: 495.3450). ^1^H NMR (400 MHz, CDCl_3_) δ (ppm): 5.63 (1H, s), 5.34 (2H, m), 5.11 (1H, t, *J* = 7.4 Hz), 4.78 (1H, t, *J* = 3.0 Hz), 4.67 (2H, s), 2.75 (2H, s), 2.60 (1H, m), 2.41 (1H, m), 2.33 (2H, t, *J* = 7.5 Hz), 2.00 (4H, s), 1.72 (3H, s), 1.63 (3H, s), 1.27 (22H, m), 0.87 (3H, t, *J* = 6.5 Hz). ^13^C NMR (100 MHz, CDCl_3_) δ (ppm): 173.3, 172.4, 141.5, 136.2, 129.9, 129.8, 124.9, 117.1, 78.2, 75.3, 61.8, 37.9, 37.7, 34.1, 31.7, 29.7, 29.5, 29.4, 29.3, 29.2, 29.1, 29.0, 27.5, 27.2, 27.1, 25.8, 24.9, 22.6, 18.0, 14.1.

#### ((1R,5S)-1-(3-methylbut-2-en-1-yl)-7-oxo-6-oxabicyclo[3.2.0]hept-2-en-3-yl)methylpentadecanoate (**A40**)

Yield: 88 %. HR-ESI-MS *m/z*: 455.3138 [M + Na]^+^ (Calcd. for C_27_H_44_O_4_Na: 455.3137). ^1^H NMR (400 MHz, CDCl_3_) δ (ppm): 5.63 (1H, s), 5.11 (1H, t, *J* = 7.0 Hz), 4.78 (1H, s), 4.65 (2H, s), 2.74 (2H, s), 2.60 (1H, m), 2.41 (1H, m), 2.33 (2H, t, *J* = 7.5 Hz), 2.17 (1H, s), 1.72 (3H, s), 1.63 (3H, s), 1.24 (23H, m), 0.87 (3H, t, *J* = 6.4 Hz). ^13^C NMR (100 MHz, CDCl_3_) δ (ppm): 173.3, 172.4, 141.5, 136.2, 124.9, 117.1, 78.1, 75.3, 61.7, 37.7, 34.1, 31.9, 29.6, 29.5, 29.4, 29.3, 29.2, 29.1, 27.5, 25.8, 24.9, 22.7, 18.0, 14.1.

#### ((1R,5S)-1-(3-methylbut-2-en-1-yl)-7-oxo-6-oxabicyclo[3.2.0]hept-2-en-3-yl)methyl-3-(pyridin-3-yl)propanoate (**A41**)

Yield: 83 %. HR-EI-MS *m/z*: 341.1627 [M]^+^ (Calcd. for C_20_H_23_NO_4_: 341.1627). ^1^H NMR (400 MHz, CDCl_3_) δ (ppm): 8.48 (2H, m), 7.59 (1H, d, *J* = 7.7 Hz),7.28 (1H, m), 5.58 (1H, s), 5.09 (1H, t, *J* = 7.3 Hz), 4.76 (1H, t, *J* = 3.2 Hz), 4.65 (2H, s), 2.98 (2H, t, *J* = 7.5 Hz), 2.70 (4H, m), 2.58 (1H, m), 2.39 (1H, m), 1.72 (3H, s), 1.63 (3H, s). ^13^C NMR (100 MHz, CDCl_3_) δ (ppm): 172.3, 171.6, 149.0, 147.1, 141.0, 136.7, 136.2, 136.0, 125.4, 123.7, 117.0, 78.1, 75.4, 62.1, 37.7, 35.0, 27.9, 27.5, 25.8, 18.0.

#### (S)-((1R,5S)-1-(3-methylbut-2-en-1-yl)-7-oxo-6-oxabicyclo[3.2.0]hept-2-en-3-yl)methyl 2-formamido-3-(4-hydroxyphenyl)propanoate (**A42**)

Yield: 80 %. HR-EI-MS *m/z*: 399.1670 [M]^+^ (Calcd. for C_22_H_25_O_6_N: 399.1682). ^1^H NMR (500 MHz, CDCl_3_) δ (ppm): 8.20 (1H, s), 6.98 (2H, d, *J* = 8.5 Hz), 6.75 (2H, d, *J* = 8.5 Hz), 6.14 (1H, s), 5.08 (1H, m), 4.90 (1H, m), 4.78 (1H, s), 4.72 (1H, d, *J* = 14.7 Hz), 4.54 (1H, d, *J* = 14.7 Hz) 3.10 (1H, m), 2.96 (1H, m), 2.62 (4H, m), 2.41 (1H, m), 1.92 (1H, m), 1.72 (3H, s), 1.64 (3H, s).^13^C NMR (100 MHz, CDCl_3_) δ (ppm): 173.1, 171.0, 160.7, 155.4, 139.9, 136.4, 130.3, 127.2, 125.0, 116.9, 116.2, 115.6, 78.4, 75.4, 62.8, 52.1, 36.7, 36.6, 32.5, 27.4, 25.7, 24.8, 18.0.

#### (Z)-((1R,5S)-1-(3-methylbut-2-en-1-yl)-7-oxo-6-oxabicyclo[3.2.0]hept-2-en-3-yl)methyl tetradec-9-enoate (**A43**)

Yield: 73 %. ESI-MS *m/z*: 439 [M + Na]^+^. ^1^H NMR (400 MHz, CDCl_3_) δ (ppm): 5.63 (1H, s), 5.34 (2H, m), 5.11 (1H, t, *J* = 7.5 Hz), 4.79 (1H, t, *J* = 3.0 Hz), 4.66 (2H, s), 2.75 (2H, s), 2.61 (1H, m), 2.42 (1H, m), 2.34 (2H, t, *J* = 7.5 Hz), 2.01 (4H, s), 1.72 (3H, s), 1.63 (3H, s), 1.56 (4H, s), 1.30 (10H, m), 0.90 (3H, t, *J* = 6.5 Hz). ^13^C NMR (125 MHz, CDCl_3_) δ (ppm): 165.8, 141.5, 129.9, 129.7, 125.0, 117.1, 78.1, 75.4, 61.7, 37.8, 34.3, 34.1, 31.9, 29.6, 29.1, 29.0, 27.6, 27.1, 26.9, 25.7, 24.9, 22.3, 17.9, 13.9.

#### (E)-((1R,5S)-1-(3-methylbut-2-en-1-yl)-7-oxo-6-oxabicyclo[3.2.0]hept-2-en-3-yl)methyl octadec-9-enoate (**A44**)

Yield: 78 %. HR-ESI-MS *m/z*: 495.3448 [M + Na]^+^ (Calcd. for C_30_H_48_O_4_Na: 495.3450). ^1^H NMR (400 MHz, CDCl_3_) δ (ppm): 5.63 (1H, s), 5.38 (2H, m), 5.11 (1H, t, *J* = 6.1 Hz), 4.78 (1H, t, *J* = 2.7 Hz), 4.68 (2H, s), 2.75 (2H, s), 2.60 (1H, m), 2.41 (1H, m), 2.33 (2H, m), 1.96 (4H, s), 1.72 (3H, s), 1.63 (3H, s), 1.27 (22H, m), 0.87 (3H, t, *J* = 6.6 Hz). ^13^C NMR (100 MHz, CDCl_3_) δ (ppm): 173.3, 172.4, 141.5, 136.2, 134.1, 130.5, 124.9, 117.1, 78.2, 75.3, 61.8, 37.9, 37.7, 34.1, 32.6, 32.5, 31.9, 29.6, 29.5, 29.4, 29.3, 29.2, 29.1, 28.9, 27.5, 25.8, 24.9, 22.6, 18.0, 14.1.

#### ((1R,5S)-1-(3-methylbut-2-en-1-yl)-7-oxo-6-oxabicyclo[3.2.0]hept-2-en-3-yl)methyl 3-methoxypropanoate (**A45**)

Yield: 80 %. HR-ESI-MS *m/z*: 317.1369 [M + Na]^+^ (Calcd. for C_16_H_22_O_5_Na: 317.1364). ^1^H NMR (400 MHz, CDCl_3_) δ (ppm): 5.63 (1H, s), 5.11 (1H, m), 4.78 (2H, t, *J* = 3.0 Hz), 4.70 (2H, s), 3.67 (2H, t, *J* = 6.2 Hz), 3.36 (3H, s), 2.75 (2H, s), 2.60 (3H, m), 2.42 (1H, m), 1.72 (3H, s), 1.63 (3H, s).^13^C NMR (100 MHz, CDCl_3_) δ (ppm): 172.4, 171.1, 141.3, 136.2, 125.1, 117.1, 78.2, 75.4, 67.8, 62.0, 58.8, 37.7, 34.9, 27.5, 25.8, 18.0.

#### (Z)-((1R,5S)-1-(3-methylbut-2-en-1-yl)-7-oxo-6-oxabicyclo[3.2.0]hept-2-en-3-yl)methyl hexadec-9-enoate (**A46**)

Yield: 85 %. HR-ESI-MS *m/z*: 467.3126 [M + Na]^+^ (Calcd. for C_28_H_44_O_4_Na: 467.3137). ^1^H NMR (400 MHz, CDCl_3_) δ (ppm): 5.63 (1H, s), 5.34 (2H, m), 5.11 (1H, t, *J* = 6.9 Hz), 4.79 (1H, s), 4.66 (2H, s), 2.75 (2H, s), 2.61 (1H, m), 2.42 (1H, m), 2.34 (2H, t, *J* = 7.5 Hz), 2.00 (4H, m), 1.72 (3H, s), 1.63 (3H, s), 1.28 (18H, m), 0.87 (3H, t, *J* = 6.0 Hz). ^13^C NMR (100 MHz, CDCl_3_) δ (ppm): 173.1, 172.4, 141.5, 136.2, 130.5, 128.9, 125.0, 117.1, 78.1, 75.4, 61.8, 37.7, 34.0, 31.9, 29.7, 29.6, 29.5, 29.3, 29.2, 29.1, 27.5, 27.2, 26.8, 25.8, 24.5, 22.7, 18.0, 14.1.

#### (E)-((1R,5S)-1-(3-methylbut-2-en-1-yl)-7-oxo-6-oxabicyclo[3.2.0]hept-2-en-3-yl)methyl tridec-2-enoate (**A47**)

Yield: 81 %. HR-ESI-MS *m/z*: 425.2673 [M + Na]^+^ (Calcd. for C_25_H_38_O_4_Na: 425.2667). ^1^H NMR (400 MHz, CDCl_3_) δ (ppm): 5.68 (1H, s), 5.56 (2H, m), 5.11 (1H, t, *J* = 7.3 Hz), 4.78 (1H, t, *J* = 2.9 Hz), 4.67 (2H, s), 3.14-3.05 (2H, dd, *J* = 6.6 Hz, *J* = 26.5 Hz), 2.75 (2H, s), 2.60 (1H, m), 2.42 (1H, m), 2.04 (2H, m), 1.72 (3H, s), 1.63 (3H, s), 1.59 (2H, s), 1.26 (12H, m), 0.88 (3H, t, *J* = 6.5 Hz). ^13^C NMR (100 MHz, CDCl_3_) δ (ppm): 171.6, 171.5, 141.3, 136.2, 135.4, 134.0, 125.0, 120.9, 120.2, 117.1, 78.1, 75.4, 62.0, 37.9, 37.7, 32.8, 31.9, 29.6, 29.5, 29.4, 29.3, 29.2, 29.1, 27.5, 27.4, 25.8, 22.6, 18.0, 14.0.

#### (S)-((1R,5S)-1-(3-methylbut-2-en-1-yl)-7-oxo-6-oxabicyclo[3.2.0]hept-2-en-3-yl)methyl 2-formamido-3-methylbutanoate (**A48**)

Yield: 81 %. HR-EI-MS *m/z*: 335.1682 [M]^+^ (Calcd. for C_18_H_25_O_5_N: 335.1733). ^1^H NMR (400 MHz, CDCl_3_) δ (ppm): 8.26 (1H, s), 6.23 (1H, brs), 5.67 (1H, s), 5.09 (1H, m), 4.79 (1H, s), 4.73 (2H, s), 4.67 (1H, brs), 2.76 (2H, s), 2.61 (1H, m), 2.42 (1H, m), 2.21 (1H, m), 1.71 (3H, s), 1.62 (3H, s), 0.97(3H, d, *J* = 6.8 Hz), 0.91 (3H, d, *J* = 6.8 Hz). ^13^C NMR (100 MHz, CDCl_3_) δ (ppm): 171.7, 171.1, 140.5, 136.3, 126.3, 126.0, 116.9, 78.1, 75.4, 62.8, 55.6, 37.8, 31.2, 27.4, 25.8, 19.0, 18.0, 17.6.

#### ((1R,5S)-1-(3-methylbut-2-en-1-yl)-7-oxo-6-oxabicyclo[3.2.0]hept-2-en-3-yl)methyl 2-(2-(2-methoxyethoxy)ethoxy)acetate (**A49**)

Yield: 88 %. HR-EI-MS *m/z*: 368.1826 [M]^+^ (Calcd. for C_19_H_28_O_7_: 368.1835). ^1^H NMR (400 MHz, CDCl_3_) δ (ppm): 5.66 (1H, s), 5.10 (1H, m), 4.79 (1H, m), 4.74 (2H, s), 4.20 (2H, s), 3.75 (2H, m), 3.70 (2H, m), 3.65 (2H, m), 3.55 (2H, m), 3.38 (3H, s), 2.76 (2H, s), 2.62 (1H, m), 2.40 (1H, m), 1.95 (2H, m), 1.71 (3H, s), 1.64 (3H, s). ^13^C NMR (100 MHz, CDCl_3_) δ (ppm): 172.2, 170.0, 140.8, 136.3, 125.7, 117.0, 78.1, 75.4, 71.9, 71.0, 70.6, 70.5, 68.5, 62.2, 59.0, 37.8, 27.5, 25.8, 18.0.

#### ((1R,5S)-1-(3-methylbut-2-en-1-yl)-7-oxo-6-oxabicyclo[3.2.0]hept-2-en-3-yl)methyl heptadecanoate (**A50**)

Yield: 86 %. HR-ESI-MS *m/z*: 483.3460 [M + Na]^+^ (Calcd. for C_29_H_48_O_4_Na: 483.3450). ^1^H NMR (400 MHz, CDCl_3_) δ (ppm): 5.63 (1H, s), 5.11 (1H, t, *J* = 7.3 Hz), 4.78 (1H, s), 4.65 (2H, s), 2.75 (2H, s), 2.60 (1H, m), 2.41 (1H, m), 2.33 (2H, t, *J* = 7.5 Hz), 1.72 (3H, s), 1.63 (3H, s), 1.24 (28H, m), 0.87 (3H, t, *J* = 6.4 Hz). ^13^C NMR (100 MHz, CDCl_3_) δ (ppm): 173.3, 172.4, 141.5, 136.2, 124.9, 117.1, 78.1, 75.3, 61.7, 37.7, 34.1, 31.9, 29.7, 29.6, 29.5, 29.4, 29.3, 29.2, 29.1, 27.5, 25.8, 24.9, 22.7, 18.0, 14.1.

#### ((1R,5S)-1-(3-methylbut-2-en-1-yl)-7-oxo-6-oxabicyclo[3.2.0]hept-2-en-3-yl)methyl 4-(naphthalen-2-yl)butanoate (**A51**)

Yield: 80 %. HR-EI-MS *m/z*: 404.1988 [M]^+^ (Calcd. for C_26_H_28_O_4_: 404.2002). ^1^H NMR (400 MHz, CDCl_3_) δ (ppm): 8.18 (1H, t, *J* = 4.2 Hz), 7.81 (1H, t, *J* = 3.7 Hz), 7.48 (4H, m), 6.84 (1H, d, *J* = 7.0 Hz), 5.63 (1H, s), 5.11 (1H, m), 4.80 (1H, s), 4.67 (2H, s), 2.75 (2H, s), 2.60 (1H, m), 2.41 (1H, m), 2.34 (2H, t, *J* = 7.3 Hz), 1.72 (3H, s), 1.66 (4H, m), 1.63 (3H, s). ^13^C NMR (125 MHz, CDCl_3_) δ (ppm): 172.4, 172.1, 157.4, 141.6, 127.6, 126.4, 125.8, 125.2, 125.1, 120.5, 117.1, 108.6, 78.2, 75.4, 37.8, 36.0, 32.2, 27.6, 25.9, 24.5, 18.4.

#### ((1R,5S)-1-(3-methylbut-2-en-1-yl)-7-oxo-6-oxabicyclo[3.2.0]hept-2-en-3-yl)methyl 2-acetoxyacetate (**A52**)

Yield: 85 %. HR-ESI-MS *m/z*: 331.0938[M + Na]^+^ (Calcd. for C_19_H_16_O_4_Na: 331.0946). ^1^H NMR (400 MHz, CDCl_3_) δ (ppm): 5.66 (1H, s), 5.11 (1H, t, *J* = 7.4 Hz), 4.79 (2H, m), 4.76 (2H, s), 4.64 (2H, s), 2.76 (2H, s), 2.62 (1H, m), 2.42 (1H, m), 2.17 (3H, s), 1.73 (3H, s), 1.64 (3H, s). ^13^C NMR (100 MHz, CDCl_3_) δ (ppm): 172.2, 170.3, 167.4, 140.5, 136.3, 126.0, 117.0, 113.8, 78.1, 75.4, 62.7, 60.5, 37.7, 27.5, 25.8, 20.4, 18.0.

#### ((1R,5S)-1-(3-methylbut-2-en-1-yl)-7-oxo-6-oxabicyclo[3.2.0]hept-2-en-3-yl)methyl oleate (**A53**)

Yield: 82 %. HR-ESI-MS *m/z*: 495.3453 [M + Na]^+^ (Calcd. for C_30_H_48_O_4_Na: 495.3450). ^1^H NMR (500 MHz, CDCl_3_) δ (ppm): 5.63 (1H, s), 5.34 (2H, m), 5.11 (1H, t, *J* = 6.9 Hz), 4.78 (1H, t, *J* = 3.0 Hz), 4.65 (2H, s), 2.75 (2H, s), 2.61 (1H, m), 2.42 (1H, m), 2.34 (2H, t, *J* = 7.5 Hz), 2.00 (4H, s), 1.72 (3H, s), 1.63 (3H, s), 1.26 (22H, m), 0.87 (3H, t, *J* = 6.5 Hz). ^13^C NMR (125 MHz, CDCl_3_) δ (ppm): 173.2, 172.3, 141.6, 136.2, 130.0, 129.7, 125.1, 117.2, 78.2, 75.4, 61.8, 37.8, 34.1, 31.9, 29.8, 29.7, 29.5, 29.3, 29.1, 29.0, 27.6, 27.2, 27.1, 25.8, 24.9, 22.7, 18.0, 14.0.

#### ((1R,5S)-1-(3-methylbut-2-en-1-yl)-7-oxo-6-oxabicyclo[3.2.0]hept-2-en-3-yl)methyl 2-(2-methoxyethoxy)acetate (**A54**)

Yield: 80 %. HR-EI-MS *m/z*: 324.1546 [M]^+^ (Calcd. for C_17_H_24_O_6_: 324.1573). ^1^H NMR (400 MHz, CDCl_3_) δ (ppm): 5.66 (1H, s), 5.10 (1H, m), 4.78 (1H, m), 4.74 (2H, s), 4.20 (2H, s), 3.73 (2H, m), 3.58 (2H, m), 3.39 (3H, s), 2.76 (2H, s), 2.62 (1H, m), 2.42 (1H, m), 1.95 (2H, m), 1.72 (3H, s), 1.64 (3H, s). ^13^C NMR (100 MHz, CDCl_3_) δ (ppm): 172.2, 170.0, 140.8, 136.3, 125.7, 117.0, 78.1, 75.4, 71.9, 70.9, 68.5, 62.2, 59.0, 37.7, 27.5, 25.8, 18.0.

### General Procedure to Synthesize Compounds **B1**–**B33**

A solution of the vibralactone (208 mg, 1.0 mmol) in dichloromethane (5 mL) was cooled to 0 °C, and PCC reagent (1.2 mmol) was slowly added. Stirring was continued for 1 h at 0 °C. The reaction was monitored by TLC. Following complete reaction of the starting material, the reaction mixture was quenched by water. The aqueous layer was extracted with Et_2_O (3 × 30 mL). The combined organic extracts were washed with brine and dried over Na_2_SO_4_, filtered and concentrated in vacuo. The resultant oil was purified by chromatography on silica gel with petroleum ether/ethyl acetate (8:1) as the eluent, yielding the title compound as a colorless oil (199 mg, 96 %).

To the aldehyde (166 mg, 0.81 mmol) in 8 mL of THF at −78 °C was added C_6_H_13_MgBr (0.81 mL, 1.62 mmol, 2.0 M in Et_2_O) dropwise. The mixture was stirred for 6 h at −78 °C and then warmed to room temperature. The reaction was quenched by the addition of 5 % NH_4_OH. The aqueous phase was extracted several times with ethyl acetate. The combined organic extracts were washed with brine, dried over Na_2_SO_4_, filtered and concentrated *in vacuo*. The resultant oil was purified by chromatography on silica gel using petroleum ether/ethyl acetate (10:1) as the eluent, yielding the diastereoisomers. Preparative HPLC separation yielded the two pure diastereoisomers, and the absolute stereochemistry of the hydroxyl group in the products was assigned using the Mosher method. The synthesis of the Mosher ester derivatives was achieved with one of the diastereoisomers using 3.5 equivalents of DCC, 1.0 equivalent of DMAP, and either (*S*)-MTPA-Cl or (*R*)-MTPA-Cl. Mosher ester analysis confirmed the hydroxyl stereochemistry.

Representative data points for the difference in NMR chemical shift values [ppm], *i.e*., *δ*(*S*)-Mosher ester-*δ*(*R*)-Mosher ester, are shown for the ester (400 MHz, CDCl_3_). MTPA = α-methoxy-α-trifluoromethylphenylacetic acid (Mosher); DMAP = 4-(dimethylamino)pyridine.
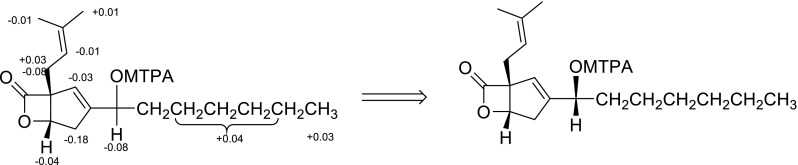


To a stirring solution of the appropriate carboxyl acids (0.12 mmol) in CH_2_Cl_2_ (2 mL) at 0 °C was added thionyl chloride (0.6 mmol) dropwise. The reaction was monitored by TLC, and following the complete reaction of the starting material, the reaction mixture was concentrated to yield a brown–yellow oil. To a solution of (1*R*,5*S*)-3-(1-hydroxyalkyl)-1-(3-methylbut-2-en-1-yl)-6-oxabicyclo[3.2.0]hept-2-en-7-one (0.1 mmol) in dichloromethane (2 mL) at 0 °C was added Et_3_N (0.2 mmol) and the corresponding acyl chloride dissolved in dichloromethane (2 mL). The reaction mixture was stirred at room temperature overnight. A saturated NH_4_Cl solution was added to quench the reaction, and the mixture was extracted with CH_2_Cl_2_ (3 × 10 mL). The combined organic layers were dried over MgSO_4_ and concentrated *in vacuo*. The products were purified using flash chromatography on silica gel.

#### (R)-(R)-1-((1R,5S)-1-(3-methylbut-2-en-1-yl)-7-oxo-6-oxabicyclo[3.2.0]hept-2-en-3-yl)heptyl-3,3,3-trifluoro-2-methoxy-2-phenylpropanoate (**B1**)

Yield: 80 %. HR-EI-MS *m/z*: 508.2449 [M]^+^ (Calcd. for C_28_H_35_O_5_F_3_: 508.2437). ^1^H NMR (600 MHz, CDCl_3_) δ (ppm): 7.38 (5H, m), 5.63 (2H, m), 5.02 (1H, t, *J* = 7.2 Hz), 4.74 (1H, brs), 3.46 (3H, s), 2.70 (2H, s), 2.56 (1H, m), 2.38 (1H, m), 1.68 (3H, s), 1.59 (3H, s), 1.17 (10H, m), 0.83 (3H, t, *J* = 7.2 Hz). ^13^C NMR (150 MHz, CDCl_3_) δ (ppm): 172.3, 166.1, 143.8, 136.5, 132.3, 129.9, 128.7, 127.5, 126.5, 124.5, 122.5, 117.1, 78.3, 75.4, 74.0, 55.5, 36.8, 32.5, 31.7, 28.9, 27.7, 26.1, 25.4, 22.7, 18.2, 14.2.

#### (S)-1-((1R,5S)-1-(3-methylbut-2-en-1-yl)-7-oxo-6-oxabicyclo[3.2.0]hept-2-en-3-yl)heptyl-3-phenylpropanoate (**B2**)

Yield: 87 %. HR-EI-MS *m/z*: 424.2620 [M]^+^ (Calcd. for C_27_H_36_O_4_: 424.2614). ^1^H NMR (400 MHz, CDCl_3_) δ (ppm): 7.31 (2H, m), 7.22 (3H, m), 5.52 (1H, s), 5.43 (1H, t, *J* = 6.4 Hz), 5.07 (1H, t, *J* = 7.3 Hz), 4.73 (1H, brs), 2.98 (2H, t, *J* = 7.6 Hz), 2.66 (4H, m), 2.56 (1H, m), 2.36 (1H, m), 1.74 (3H, s), 1.66 (3H, s), 1.60 (4H, m), 1.27 (6H, s), 0.90 (3H, t, *J* = 6.6 Hz). ^13^C NMR (100 MHz, CDCl_3_) δ (ppm): 172.6, 172.0, 144.9, 140.3, 136.1, 128.5, 128.3, 126.3, 124.5, 117.2, 77.9, 75.1, 72.2, 36.2, 35.9, 32.8, 31.6, 31.0, 29.0, 27.6, 25.8, 24.8, 22.5, 18.0, 14.0.

#### (R)-1-((1R,5S)-1-(3-methylbut-2-en-1-yl)-7-oxo-6-oxabicyclo[3.2.0]hept-2-en-3-yl)heptyl-6-(4-fluorophenyl)hexanoate (**B3**)

Yield: 84 %. HR-EI-MS *m/z*: 484.2979 [M]^+^ (Calcd. for C_30_H_41_O_4_F: 484.2989). ^1^H NMR (600 MHz, CDCl_3_) δ (ppm): 7.08 (2H, m), 6.93 (2H, m), 5.52 (1H, s), 5.36 (1H, t, *J* = 6.0 Hz), 5.05 (1H, m), 4.74 (1H, brs), 2.70 (2H, s), 2.55 (3H, m), 2.37 (1H, m), 2.31 (2H, t, *J* = 7.2 Hz), 1.69 (3H, s), 1.65 (6H, m), 1.61 (3H, s), 1.30 (10H, m), 0.85 (3H, t, *J* = 7.2 Hz). ^13^C NMR (100 MHz, CDCl_3_) δ (ppm): 173.1, 172.8, 162.1, 160.5, 145.5, 138.2, 136.3, 129.8, 124.1, 117.3, 115.2, 115.1, 78.4, 75.3, 71.5, 37.1, 35.3, 35.0, 32.9, 31.8, 31.4, 28.5, 26.0, 25.2, 22.8, 18.2, 14.3.

#### (R)-1-((1R,5S)-1-(3-methylbut-2-en-1-yl)-7-oxo-6-oxabicyclo[3.2.0]hept-2-en-3-yl)heptyl-3-phenylpropanoate (**B4**)

Yield: 83 %. HR-EI-MS *m/z*: 424.2618 [M]^+^ (Calcd. for C_27_H_36_O_4_: 424.2614). ^1^H NMR (400 MHz, CDCl_3_) δ (ppm): 7.28 (2H, m), 7.17 (3H, m), 5.44 (1H, s), 5.37 (1H, t, *J* = 6.5 Hz), 5.07 (1H, t, *J* = 7.3 Hz), 4.74 (1H, brs), 2.96 (2H, t, *J* = 7.6 Hz), 2.67 (4H, m), 2.56 (1H, m), 2.36 (1H, m), 1.71 (3H, s), 1.62 (3H, s), 1.60 (4H, m), 1.24 (6H, s), 0.87 (3H, t, *J* = 6.6 Hz). ^13^C NMR (100 MHz, CDCl_3_) δ (ppm): 172.6, 172.0, 145.2, 140.2, 136.1, 128.5, 128.2, 126.3, 123.8, 117.1, 78.2, 75.1, 71.6, 37.0, 35.8, 32.6, 31.6, 30.9, 28.9, 27.6, 25.8, 24.8, 22.5, 18.0, 14.0.

#### (S)-(R)-1-((1R,5S)-1-(3-methylbut-2-en-1-yl)-7-oxo-6-oxabicyclo[3.2.0]hept-2-en-3-yl)nonyl-2-formamido-4-methylpentanoate (**B5**)

Yield: 68 %. HR-ESI-MS *m/z*: 484.3034 [M + Na]^+^ (Calcd. for C_27_H_43_NO_5_Na: 484.3039). ^1^H NMR (400 MHz, CDCl_3_) δ (ppm): 8.21 (1H, s), 5.97 (1H, brs), 5.61 (1H, s), 5.40 (1H, m), 5.08 (1H, m), 4.76 (1H, m), 4.72 (1H, m), 2.73 (2H, s), 2.60 (1H, m), 2.42 (1H, m), 1.72 (3H, s), 1.63 (3H, s), 1.26 (17H, s), 0.97 (6H, m), 0.88 (3H, t, *J* = 6.4 Hz). ^13^C NMR (100 MHz, CDCl_3_) δ (ppm): 172.4, 171.9, 160.6, 144.5, 136.2, 125.4, 124.6, 117.1, 78.1, 75.3, 72.9, 49.6, 41.8, 37.2, 36.7, 32.5, 31.8, 29.4, 29.2, 27.6, 25.8, 24.9, 24.9, 22.8, 22.6, 21.9, 18.0, 14.0.

#### (S)-1-((1R,5S)-1-(3-methylbut-2-en-1-yl)-7-oxo-6-oxabicyclo[3.2.0]hept-2-en-3-yl)heptyl-5-(4-methoxyphenyl)pentanoate (**B6**)

Yield: 75 %. HR-EI-MS *m/z*: 482.3038 [M]^+^ (Calcd. for C_30_H_42_O_5_: 482.3032). ^1^H NMR (400 MHz, CDCl_3_) δ (ppm): 7.08 (2H, d, *J* = 8.4 Hz), 6.82 (2H, d, *J* = 8.8 Hz), 5.58 (1H, s), 5.43 (1H, t, *J* = 6.4 Hz), 5.30 (1H, m), 4.74 (1H, d, *J* = 5.2 Hz), 3.78 (3H, s), 2.75-2.55 (5H, m), 2.42 (1H, m), 2.33 (2H, t, *J* = 7.2 Hz), 1.71(3H, s), 1.63 (8H, s), 1.56 (3H, s), 1.25 (6H, s), 0.87 (3H, t, *J* = 7.2 Hz). ^13^C NMR (100 MHz, CDCl_3_) δ (ppm): 172.7, 157.8, 145.0, 136.1, 134.1, 132.2, 129.2, 124.5, 117.2, 113.8, 113.5, 77.9, 75.2, 71.9, 55.3, 36.4, 34.6, 34.3, 32.8, 31.6, 31.1, 29.7, 28.9, 27.6, 25.8, 24.9, 24.6, 22.5, 18.0, 14.1.

#### (S)-(S)-1-((1R,5S)-1-(3-methylbut-2-en-1-yl)-7-oxo-6-oxabicyclo[3.2.0]hept-2-en-3-yl)heptyl-3,3,3-trifluoro-2-methoxy-2-phenylpropanoate (**B7**)

Yield: 78 %. HR-EI-MS *m/z*: 508.2438 [M]^+^ (Calcd. for C_28_H_35_O_5_F_3_: 508.2437). ^1^H NMR (600 MHz, CDCl_3_) δ (ppm): 7.45 (2H, m), 7.39 (3H, m), 5.57 (1H, t, *J* = 6.0 Hz), 5.50 (1H, s), 5.02 (1H, m), 4.70 (1H, d, *J* = 6.0 Hz), 3.54 (3H, s), 2.63 (1H, m), 2.54 (2H, m), 2.35 (1H, m), 1.71 (3H, s), 1.66 (3H, s), 1.25 (10H, m), 0.85 (3H, t, *J* = 6.6 Hz). ^13^C NMR (150 MHz, CDCl_3_) δ (ppm): 172.3, 166.0, 143.8, 136.5, 132.3, 129.9, 128.7, 127.3, 126.0, 124.4, 122.5, 117.2, 78.2, 75.4, 74.2, 55.8, 36.8, 32.6, 31.8, 29.9, 29.0, 27.8, 26.2, 25.1, 22.7, 18.2, 14.2.

#### (S)-(R)-1-((1R,5S)-1-(3-methylbut-2-en-1-yl)-7-oxo-6-oxabicyclo[3.2.0]hept-2-en-3-yl)nonyl-2-formamido-3-phenylpropanoate (**B8**)

Yield: 81 %. HR-ESI-MS *m/z*: 518.2883 [M + Na]^+^ (Calcd. for C_30_H_41_NO_5_Na: 518.2882). ^1^H NMR (400 MHz, CDCl_3_) δ (ppm): 8.16 (1H, s), 7.28 (3H, m), 7.15 (2H, m), 6.04 (1H, d, *J* = 7.6 Hz), 5.55 (1H, m), 5.36 (1H, m), 5.06 (1H, m), 4.95 (1H, m), 4.76 (1H, m), 3.17 (2H, m), 2.68 (2H, d, *J* = 8.0 Hz), 2.58 (1H, m), 2.41 (1H, m), 1.71 (3H, s), 1.62 (3H, s), 1.25 (15H, m), 0.88 (3H, t, *J* = 6.4 Hz). ^13^C NMR (100 MHz, CDCl_3_) δ (ppm): 172.4, 170.6, 160.5, 144.2, 136.3, 135.4, 129.4, 129.3, 128.7, 127.4, 124.8, 117.1, 78.1, 75.2, 73.3, 51.9, 37.8, 37.0, 32.5, 31.8, 29.3, 29.2, 27.6, 25.8, 24.8, 22.6, 18.1, 14.1.

#### (R)-1-((1R,5S)-1-(3-methylbut-2-en-1-yl)-7-oxo-6-oxabicyclo[3.2.0]hept-2-en-3-yl)nonyl-3-phenylpropanoate (**B9**)

Yield: 89 %. HR-ESI-MS *m/z*: 475.2820 [M + Na]^+^ (Calcd. for C_29_H_40_O_4_Na: 475.2824). ^1^H NMR (400 MHz, CDCl_3_) δ (ppm): 7.26 (2H, m), 7.19 (3H, m), 5.44 (1H, s), 5.36 (1H, t, *J* = 6.4 Hz), 5.07 (1H, m), 4.73 (1H, m), 2.96 (2H, t, *J* = 7.6 Hz), 2.66 (4H, m), 2.56 (1H, m), 2.36 (1H, m), 1.71 (3H, s), 1.62 (3H, s), 1.24 (14H, s), 0.88 (3H, t, *J* = 6.8 Hz). ^13^C NMR (100 MHz, CDCl_3_) δ (ppm): 172.7, 172.2, 145.5, 140.5, 136.3, 128.7, 128.5, 126.5, 124.1, 117.4, 78.4, 75.3, 71.8, 37.2, 36.0, 32.9, 32.0, 31.1, 29.6, 29.4, 27.8, 26.0, 25.1, 22.8, 18.2, 14.3.

#### (R)-1-((1R,5S)-1-(3-methylbut-2-en-1-yl)-7-oxo-6-oxabicyclo[3.2.0]hept-2-en-3-yl)heptyl-6-phenylhexanoate (**B10**)

Yield: 89 %. HR-EI-MS *m/z*: 466.3102 [M]^+^ (Calcd. for C_30_H_42_O_4_: 466.3083). ^1^H NMR (500 MHz, CDCl_3_) δ (ppm): 7.28 (2H,m), 7,17 (3H, m), 5.53 (1H, s), 5.38 (1H, t, *J* = 6.4 Hz), 5.08 (1H, t, *J* = 7.1 Hz), 4.76 (1H, brs), 2.72 (2H, s), 2.59 (3H, m), 2.40 (1H, m), 2.31 (2H, t, *J* = 7.5 Hz), 1.71 (3H, s), 1.64 (6H, m), 1.62 (3H, s), 1.36 (2H, t, *J* = 7.3 Hz), 1.25 (8H, s), 0.87 (3H, t, *J* = 6.5 Hz). ^13^C NMR (100 MHz, CDCl_3_) δ (ppm): 172.8, 172.6, 145.4, 142.4, 136.1, 128.3, 128.2, 125.7, 123.8, 117.1, 78.2, 75.1, 71.3, 37.0, 35.7, 34.3, 32.7, 31.6, 31.1, 28.9, 28.7, 27.6, 25.8, 24.9, 22.5, 18.0, 14.0.

#### (R)-1-((1R,5S)-1-(3-methylbut-2-en-1-yl)-7-oxo-6-oxabicyclo[3.2.0]hept-2-en-3-yl)heptyl-5-(4-methoxyphenyl)pentanoate (**B11**)

Yield: 79 %. HR-EI-MS *m/z*: 482.3042 [M]^+^ (Calcd. for C_30_H_42_O_5_: 482.3032). ^1^H NMR (600 MHz, CDCl_3_) δ (ppm): 7.06 (2H, d, *J* = 11.4 Hz), 6.80 (2H, d, *J* = 11.4 Hz), 5.51 (1H, s), 5.36 (1H, t, *J* = 6.0 Hz), 5.04 (1H, m), 4.74 (1H, brs), 3.76 (3H, s), 2.69 (2H, s), 2.54 (3H, m), 2.36 (1H, m), 2.31 (2H, t, *J* = 7.8 Hz), 1.68 (3H, s), 1.62 (8H, m), 1.25(9H, m), 0.85 (3H, t, *J* = 7.2 Hz). ^13^C NMR (150 MHz, CDCl_3_) δ (ppm): 173.0, 172.9, 157.9, 145.6, 136.3, 134.3, 132.5, 129.5, 124.0, 113.9, 78.4, 75.3, 71.5, 55.4, 37.3, 34.8, 34.5, 32.9, 31.8, 31.4, 29.9, 29.2, 27.8, 26.0, 25.2, 24.8, 22.8, 18.2, 14.3.

#### (R)-(R)-1-((1R,5S)-1-(3-methylbut-2-en-1-yl)-7-oxo-6-oxabicyclo[3.2.0]hept-2-en-3-yl)nonyl-3,3,3-trifluoro-2-methoxy-2-phenylpropanoate (**B12**)

Yield: 78 %. HR-EI-MS *m/z*: 536.2756 [M]^+^ (Calcd. for C_30_H_39_O_5_F_3_: 536.2750). ^1^H NMR (400 MHz, CDCl_3_) δ (ppm): 7.48 (2H, m), 7.43 (3H, m), 5.69 (2H, m), 5.08 (1H, t, *J* = 7.2 Hz), 4.79 (1H, brs), 3.52 (3H, s), 2.75 (2H, s), 2.61 (1H, m), 2.44 (1H, m), 1.74 (3H, s), 1.65 (3H, s), 1.25 (14H, m), 0.90 (3H, t, *J* = 7.2 Hz). ^13^C NMR (100 MHz, CDCl_3_) δ (ppm): 172.0, 165.9, 143.7, 136.3, 132.0, 129.7, 128.6, 127.4, 126.3, 124.5, 122.5, 117.0, 78.1, 75.3, 73.8, 55.3, 36.6, 32.5, 31.8, 29.3, 29.1, 27.5, 25.8, 24.7, 22.6, 18.0, 14.1.

#### (S)-1-((1R,5S)-1-(3-methylbut-2-en-1-yl)-7-oxo-6-oxabicyclo[3.2.0]hept-2-en-3-yl)heptyl-6-(4-fluorophenyl)hexanoate (**B13**)

Yield: 82 %. HR-EI-MS *m/z*: 484.2987 [M]^+^ (Calcd. for C_30_H_41_O_4_F: 484.2989). ^1^H NMR (600 MHz, CDCl_3_) δ (ppm): 7.13 (2H, m), 6.95 (2H, m), 5.58 (1H, s), 5.43 (1H, t, *J* = 6.0 Hz), 5.10 (1H, m), 4.76 (1H, brs), 2.68 (2H, s), 2.58 (3H, m), 2.41 (1H, m), 2.31 (2H, t, *J* = 7.2 Hz), 1.71 (3H, s), 1.65 (6H, m), 1.61 (3H, s), 1.33 (10H, m), 0.87 (3H, t, *J* = 7.2 Hz). ^13^C NMR (100 MHz, CDCl_3_) δ (ppm): 172.6, 162.2, 145.1, 138.3, 136.1, 129.7, 129.6, 124.5, 117.2, 115.1, 114.9, 77.9, 75.2, 71.8, 36.5, 34.8, 34.3, 32.8, 31.6, 31.2, 28.7, 27.6, 25.8, 24.9, 22.5, 18.0, 14.0.

#### (S)-(R)-1-((1R,5S)-1-(3-methylbut-2-en-1-yl)-7-oxo-6-oxabicyclo[3.2.0]hept-2-en-3-yl)nonyl-3,3,3-trifluoro-2-methoxy-2-phenylpropanoate (**B14**)

Yield: 79 %. HR-EI-MS *m/z*: 536.2744 [M]^+^ (Calcd. for C_30_H_39_O_5_F_3_: 536.2750). ^1^H NMR (400 MHz, CDCl_3_) δ (ppm): 7.45 (2H, m), 7.41 (3H, m), 5.61 (1H, t, *J* = 6.0 Hz), 5.55 (1H, s), 5.06 (1H, m), 4.74 (1H, d, *J* = 6.0 Hz), 3.58 (3H, s), 2.71 (1H, m), 2.66 (2H, m), 2.40 (1H, m), 1.77 (3H, s), 1.65 (3H, s), 1.27 (14H, m), 0.90 (3H, t, *J* = 6.6 Hz). ^13^C NMR (100 MHz, CDCl_3_) δ (ppm): 172.1, 165.8, 143.6, 136.2, 132.2, 129.7, 128.6, 127.2, 125.8, 124.4, 122.5, 117.2, 78.0, 75.2, 74.0, 55.6, 36.6, 31.8, 29.3, 29.2, 29.1, 27.5, 25.8, 25.0, 22.6, 18.0, 14.1.

#### (S)-1-((1R,5S)-1-(3-methylbut-2-en-1-yl)-7-oxo-6-oxabicyclo[3.2.0]hept-2-en-3-yl)heptyl-3-(3-phenoxyphenyl)propanoate (**B15**)

Yield: 89 %. HR-EI-MS *m/z*: 516.2873 [M]^+^ (Calcd. for C_33_H_40_O_5_: 516.2876). ^1^H NMR (800 MHz, CDCl_3_) δ (ppm): 7.36 (2H, t, *J* = 7.5 Hz), 7.25 (1H, t, *J* = 7.5 Hz), 7.13 (1H, t, *J* = 7.4 Hz), 7.01 (2H, d, *J* = 8.3 Hz), 6.96 (1H, d, *J* = 7.5 Hz), 6.86 (2H, d, *J* = 6.9 Hz), 5.56 (1H, s), 5.44 (1H, m), 5.12 (1H, m), 4.74 (1H, s), 2.95 (2H, t, *J* = 7.4 Hz), 2.66 (4H, m), 2.60 (1H, m), 2.43 (1H, m), 1.74 (3H, s), 1.65 (3H, s), 1.25 (10H, m), 0.90 (3H, t, *J* = 6.6 Hz). ^13^C NMR (200 MHz, CDCl_3_) δ (ppm): 172.6, 171.8, 157.4, 157.2, 144.8, 142.4, 136.1, 129.8, 124.7, 123.3, 118.9, 118.8, 117.2, 116.8, 77.9, 75.2, 72.3, 36.2, 35.7, 32.8, 31.6, 30.8, 27.6, 25.8, 24.9, 22.6, 18.1, 14.1.

#### (R)-1-((1R,5S)-1-(3-methylbut-2-en-1-yl)-7-oxo-6-oxabicyclo[3.2.0]hept-2-en-3-yl)nonyl-5-(4-methoxyphenyl)pentanoate (**B16**)

Yield: 78 %. ESI-MS m/z: 533 [M + Na]^+^. ^1^H NMR (400 MHz, CDCl_3_) δ (ppm): 7.08 (2H, d, *J* = 8.4 Hz), 6.82 (2H, d, *J* = 8.8 Hz), 5.53 (1H, s), 5.38 (1H, t, *J* = 6.4 Hz), 5.08 (1H, m), 4.75 (1H, brs), 3.78 (3H, s), 2.72(2H, s), 2.59 (3H, m), 2.35 (1H, m), 2.32 (2H, t, *J* = 7.2 Hz), 1.71(3H, s), 1.63 (6H, s), 1.61 (3H, s), 1.25 (12H, s), 0.87 (3H, t, *J* = 7.2 Hz). ^13^C NMR (100 MHz, CDCl_3_) δ (ppm): 172.7, 172.6, 157.8, 145.8, 136.1, 134.1, 132.2, 129.2, 123.8, 117.2, 113.8, 78.2, 75.1, 71.4, 55.2, 37.1, 34.6, 34.3, 32.8, 31.8, 31.1, 29.7, 29.4, 29.2, 27.6, 25.8, 25.0, 24.6, 22.6, 18.0, 14.1.

#### (S)-(R)-1-((1R,5S)-1-(3-methylbut-2-en-1-yl)-7-oxo-6-oxabicyclo[3.2.0]hept-2-en-3-yl)nonyl-2-formamidopentanoate (**B17**)

Yield: 82 %. HR-ESI-MS *m/z*: 470.2877 [M + Na]^+^ (Calcd. for C_26_H_41_NO_5_Na: 470.2882). ^1^H NMR (400 MHz, CDCl_3_) δ (ppm): 8.27 (1H, s), 6.04 (1H, d, *J* = 8.8 Hz), 5.62 (1H, d, *J* = 9.6 Hz), 5.45 (1H, m), 5.07 (1H, m), 4.77 (1H, s), 4.67 (1H, m), 2.74 (2H, s), 2.60 (1H, m), 2.41 (1H, m), 2.20 (1H, m), 1.71 (3H, s), 1.61 (3H, s), 1.26 (14H, s), 0.97 (6H, m), 0.88 (3H, m). ^13^C NMR (100 MHz, CDCl_3_) δ (ppm): 172.4, 170.9, 160.9, 144.4, 144.2, 136.2, 125.9, 117.1, 78.1, 75.2, 72.9, 55.7, 55.4, 37.1, 36.6, 32.6, 32.4, 31.8, 31.4, 29.7, 29.4, 29.2, 27.5, 25.8, 24.9, 22.6, 19.1, 18.0, 17.5, 17.2, 14.1.

#### (R)-1-((1R,5S)-1-(3-methylbut-2-en-1-yl)-7-oxo-6-oxabicyclo[3.2.0]hept-2-en-3-yl)heptyl-3-(3-phenoxyphenyl)propanoate (**B18**)

Yield: 85 %. HR-EI-MS *m/z*: 516.2878 [M]^+^ (Calcd. for C_30_H_40_O_5_: 516.2876). ^1^H NMR (500 MHz, CDCl_3_) δ (ppm): 7.34 (2H, t, *J* = 7.5 Hz), 7.25 (1H, t, *J* = 7.5 Hz), 7.10 (1H, t, *J* = 7.4 Hz), 7.00 (2H, d, *J* = 8.3 Hz), 6.94 (1H, d, *J* = 7.5 Hz), 6.85 (2H, d, *J* = 6.9 Hz), 5.47 (1H, s), 5.36 (1H, m), 5.05 (1H, m), 4.74 (1H, s), 2.93 (2H, t, *J* = 7.4 Hz), 2.65 (4H, m), 2.55 (1H, m), 2.40 (1H, m), 1.73 (3H, s), 1.64 (3H, s), 1.24 (10H, s), 0.87 (3H, t, *J* = 6.6 Hz). ^13^C NMR (150 MHz, CDCl_3_) δ (ppm): 172.9, 172.1, 157.6, 145.4, 142.5, 130.0, 129.0, 127.7, 124.1, 123.5, 123.4, 119.1, 118.9, 117.4, 117.0, 78.4, 75.3, 70.5, 37.2, 35.6, 32.9, 32.7, 31.8, 31.0, 30.0, 29.1, 27.8, 26.1, 25.1, 22.8, 18.3, 14.3.

#### (S)-1-((1R,5S)-1-(3-methylbut-2-en-1-yl)-7-oxo-6-oxabicyclo[3.2.0]hept-2-en-3-yl)heptyl-6-phenylhexanoate (**B19**)

Yield: 98 %. HR-EI-MS *m/z*: 466.3079 [M]^+^ (Calcd. for C_30_H_42_O_4_: 466.3083). ^1^H NMR (800 MHz, CDCl_3_) δ (ppm): 7.29 (2H, m), 7.19 (3H, m), 5.60 (1H, s), 5.45 (1H, t, *J* = 6.4 Hz), 5.12 (1H, m), 4.77 (1H, brs), 2.77-2.61 (5H, m), 2.43 (1H, m), 2.33 (2H, t, *J* = 7.5 Hz), 1.73 (3H, s), 1.69 (6H, m), 1.65 (3H, s), 1.41-1.24 (10H, m), 0.90 (3H, t, *J* = 6.5 Hz). ^13^C NMR (200 MHz, CDCl_3_) δ (ppm): 172.9, 172.7, 145.1, 142.3, 136.1, 128.4, 128.3, 125.7, 124.5, 117.2, 78.0, 75.1, 71.9, 36.4, 35.7, 34.3, 32.8, 31.6, 31.1, 28.9, 28.8, 27.6, 25.8, 24.9, 22.6, 18.0, 14.0.

#### (S)-(S)-1-((1R,5S)-1-(3-methylbut-2-en-1-yl)-7-oxo-6-oxabicyclo[3.2.0]hept-2-en-3-yl)heptyl 2-formamido-4-methylpentanoate (**B20**)

Yield: 85 %. HR-EI-MS *m/z*: 433.2834 [M]^+^ (Calcd. for C_25_H_39_NO_5_: 433.2828). ^1^H NMR (400 MHz, CDCl_3_) δ (ppm): 8.20 (1H, d, *J* = 7.2 Hz), 6.05 (1H, s), 5.61 (1H, s), 5.46 (1H, m), 5.10 (1H, m), 4.78 (1H, m), 4.72 (1H, m), 2.73 (2H, m), 2.60 (1H, m), 2.42 (1H, m), 1.72 (3H, s), 1.64 (3H, s), 1.26 (13H, s), 0.97 (6H, m), 0.88 (3H, t, *J* = 5.6 Hz). ^13^C NMR (100 MHz, CDCl_3_) δ (ppm): 172.4, 171.7, 160.6, 144.2, 136.2, 125.4, 117.0, 77.8, 75.1, 73.5, 49.5, 41.7, 36.3, 35.9, 32.7, 31.5, 29.7, 28.8, 27.5, 25.8, 24.9, 24.8, 22.8, 22.7, 21.9, 18.0, 14.0.

#### (Z)-(R)-1-((1R,5S)-1-(3-methylbut-2-en-1-yl)-7-oxo-6-oxabicyclo[3.2.0]hept-2-en-3-yl)heptyl-dodec-5-enoate (**B21**)

Yield: 87 %. HR-EI-MS *m/z*: 472.3529 [M]^+^ (Calcd. for C_30_H_48_O_4_: 472.3553). ^1^H NMR (400 MHz, CDCl_3_) δ (ppm): 5.54 (1H,s), 5.45-5.29 (3H, m), 5.08 (1H, t, *J* = 7.3 Hz), 4.77 (1H, brs), 2.73 (2H, s), 2.58 (1H, m), 2.42 (1H, m), 2.32 (2H, t, *J* = 7.7 Hz), 2.10-1.98 (4H, m), 1.71 (3H, s), 1.66 (6H, m), 1.62 (3H, s), 1.27 (12H, s), 0.86 (6H, s). ^13^C NMR (100 MHz, CDCl_3_) δ (ppm): 172.8, 172.6, 145.4, 136.1, 131.2, 128.2, 123.7, 117.1, 78.2, 75.1, 71.3, 37.1, 33.8, 32.7, 31.7, 31.6, 29.6, 29.0, 27.6, 27.2, 26.5, 25.8, 25.0, 24.9, 22.6, 22.5, 18.0, 14.1, 14.0.

#### (S)-(S)-1-((1R,5S)-1-(3-methylbut-2-en-1-yl)-7-oxo-6-oxabicyclo[3.2.0]hept-2-en-3-yl)heptyl-2-formamido-3-phenylpropanoate (**B22**)

Yield: 86 %. HR-EI-MS *m/z*: 467.2695 [M]^+^ (Calcd. for C_28_H_37_NO_5_: 467.2672). ^1^H NMR (400 MHz, CDCl_3_) δ (ppm): 8.16 (1H, brs), 7.28 (3H, m), 7.13 (2H, m), 6.08 (1H, s), 5.47 (2H, m), 5.10 (1H, m), 4.96 (1H, m), 4.73 (1H, m), 3.12 (2H, m), 2.63 (3H, m), 2.42 (2H, m), 1.72 (3H, s), 1.65 (3H, s), 1.63 (3H, m), 1.25 (6H, s), 0.88 (3H, t, *J* = 4.0 Hz). ^13^C NMR (100 MHz, CDCl_3_) δ (ppm): 172.3, 170.5, 160.5, 143.9, 136.3, 135.4, 129.3, 128.7, 127.3, 125.5, 117.1, 77.8, 75.2, 73.8, 51.9, 38.1, 36.1, 32.7, 31.5, 28.8, 27.5, 25.8, 24.8, 22.5, 18.1, 14.0;

#### (S)-(R)-1-((1R,5S)-1-(3-methylbut-2-en-1-yl)-7-oxo-6-oxabicyclo[3.2.0]hept-2-en-3-yl)heptyl-2-formamido-4-methylpentanoate (**B23**)

Yield: 86 %. HR-EI-MS *m/z*: 433.2819 [M]^+^ (Calcd. for C_25_H_39_O_5_N: 433.2828). ^1^H NMR (400 MHz, CDCl_3_) δ (ppm): 8.11 (1H, s), 5.65 (1H, m), 5.45 (1H, m), 5.15 (1H, m), 4.89 (1H, m), 4.55 (1H, m), 2.80 (2H, m), 2.62 (1H, m), 2.45 (1H, m), 1.77 (3H, s), 1.65 (3H, s), 1.32 (14H, m), 0.99 (9H, m). ^13^C NMR (100 MHz, CDCl_3_) δ (ppm): 173.1, 171.4, 162.2, 144.9, 144.7, 135.5, 124.9, 124.2, 117.3, 78.5, 75.0, 72.6, 49.7, 40.3, 36.4, 36.0, 32.1, 31.5, 28.5, 27.0, 24.6, 22.2, 20.6, 16.7, 13.0.

#### (R)-1-((1R,5S)-1-(3-methylbut-2-en-1-yl)-7-oxo-6-oxabicyclo[3.2.0]hept-2-en-3-yl)nonyl-3-(3-phenoxyphenyl)propanoate (**B24**)

Yield: 94 %. HR-ESI-MS *m/z*: 567.3076 [M + Na]^+^ (Calcd. for C_35_H_44_O_5_Na: 567.3086). ^1^H NMR (400 MHz, CDCl_3_) δ (ppm): 7.33 (2H, t, *J* = 8.9 Hz), 7.24 (1H, t, *J* = 7.6 Hz), 7.09 (1H, t, *J* = 7.6 Hz), 7.00 (2H, d, *J* = 7.6 Hz), 6.94 (1H, d, *J* = 13.6 Hz), 6.85 (2H, d, *J* = 6.9 Hz), 5.48 (1H, s), 5.37 (1H, m), 5.08 (1H, m), 4.74 (1H, s), 2.93 (2H, t, *J* = 7.6 Hz), 2.66 (4H, m), 2.58 (1H, m), 2.36 (1H, m), 1.71 (3H, s), 1.62 (3H, s), 1.24 (12H, m), 0.88 (3H, t, *J* = 7.6 Hz). ^13^C NMR (100 MHz, CDCl_3_) δ (ppm): 172.6, 171.8, 157.5, 157.2, 145.3, 142.4, 136.1, 129.8, 124.0, 123.3, 118.9, 118.8, 117.3, 116.8, 78.2, 75.2, 71.8, 37.1, 35.7, 32.7, 31.9, 30.8, 29.4, 29.3, 27.7, 25.9, 25.0, 22.7, 18.1, 14.2.

#### (S)-(R)-1-((1R,5S)-1-(3-methylbut-2-en-1-yl)-7-oxo-6-oxabicyclo[3.2.0]hept-2-en-3-yl)heptyl 2-formamido-3-phenylpropanoate (**B25**)

Yield: 83 %. HR-EI-MS *m/z*: 467.2671 [M]^+^ (Calcd. for C_28_H_37_NO_5_: 467.2672). ^1^H NMR (800 MHz, CDCl_3_) δ (ppm): 8.17 (1H, s), 7.30 (3H, m), 7.17 (2H, m), 6.13 (1H, m), 5.57 (1H, m), 5.38 (1H, m), 5.10 (1H, m), 4.96 (1H, m), 4.78 (1H, m), 3.17 (2H, m), 2.70 (2H, m), 2.60 (1H, m), 2.43 (1H, m), 1.73 (3H, s), 1.65 (3H, s), 1.28 (11H, m), 0.90 (3H, t, *J* = 4.0 Hz). ^13^C NMR (200 MHz, CDCl_3_) δ (ppm): 172.4, 170.5, 160.5, 144.3, 136.3, 135.4, 129.3, 128.7, 127.4, 124.8, 117.1, 78.1, 75.2, 73.8, 52.0, 37.8, 37.0, 32.5, 31.6, 28.9, 27.6, 25.8, 24.8, 22.6, 18.1, 14.1.

#### (Z)-(S)-1-((1R,5S)-1-(3-methylbut-2-en-1-yl)-7-oxo-6-oxabicyclo[3.2.0]hept-2-en-3-yl)heptyl dodec-5-enoate (**B26**)

Yield: 85 %. HR-EI-MS *m/z*: 472.3553 [M]^+^ (Calcd. for C_30_H_48_O_4_: 472.3553). ^1^H NMR (400 MHz, CDCl_3_) δ (ppm): 5.60 (1H, s), 5.45 (2H, m), 5.33 (1H, m), 5.12 (1H, m), 4.77 (1H, d, *J* = 8.0 Hz), 2.76 (2H, m), 2.63 (1H, m), 2.44 (1H, m), 2.33 (2H, t, *J* = 8.0 Hz), 2.12-2.00 (4H, m), 1.73 (3H, s), 1.68 (6H, m), 1.65 (3H, s), 1.29 (12H, s), 0.90 (6H, s). ^13^C NMR (100 MHz, CDCl_3_) δ (ppm): 172.8, 172.6, 145.1, 136.1, 131.3, 128.2, 124.5, 117.2, 77.9, 75.2, 71.9, 36.3, 33.9, 32.8, 31.8, 31.6, 29.7, 29.0, 28.9, 27.6, 27.3, 26.6, 25.8, 25.0, 24.9, 22.7, 22.5, 18.0, 14.1, 14.0.

#### (R)-1-((1R,5S)-1-(3-methylbut-2-en-1-yl)-7-oxo-6-oxabicyclo[3.2.0]hept-2-en-3-yl)nonyl-6-phenylhexanoate (**B27**)

Yield: 97 %. HR-ESI-MS *m/z*: 517.3290 [M + Na]^+^ (Calcd. for C_32_H_46_O_4_Na: 517.3294). ^1^H NMR (400 MHz, CDCl_3_) δ (ppm): 7.29 (2H, m), 7.18 (3H, m), 5.53 (1H, s), 5.38 (1H, t, *J* = 6.4 Hz), 5.08 (1H, m), 4.76 (1H, brs), 2.71(2H, s), 2.58 (3H, m), 2.41 (1H, m), 2.31 (2H, t, *J* = 7.6 Hz), 1.71 (3H, s), 1.69 (6H, m), 1.62 (3H, s), 1.38 (2H, m), 1.25 (12H, m), 0.87 (3H, t, *J* = 6.4 Hz). ^13^C NMR (100 MHz, CDCl_3_) δ (ppm): 172.8, 172.5, 145.5, 142.5, 136.1, 128.4, 128.3, 125.7, 123.9, 117.2, 78.2, 75.1, 71.3, 37.1, 35.7, 34.4, 32.8, 31.8, 31.1, 29.4, 29.3, 29.2, 28.8, 27.6, 25.8, 25.0, 22.7, 18.0, 14.1.

#### (R)-1-((1R,5S)-1-(3-methylbut-2-en-1-yl)-7-oxo-6-oxabicyclo[3.2.0]hept-2-en-3-yl)nonyl-6-(4-fluorophenyl)hexanoate (**B28**)

Yield: 96 %. HR-ESI-MS *m/z*: 535.3196 [M + Na]^+^ (Calcd. for C_32_H_45_O_4_FNa: 535.3200). ^1^H NMR (400 MHz, CDCl_3_) δ (ppm): 7.11 (2H, m), 6.95 (2H, m), 5.54 (1H, s), 5.38 (1H, t, *J* = 6.4 Hz), 5.08 (1H, m), 4.76 (1H, brs), 2.71 (2H, s), 2.58 (3H, m), 2.41 (1H, m), 2.31 (2H, t, *J* = 7.2 Hz), 1.71 (3H, s), 1.63 (6H, m), 1.61 (3H, s), 1.25 (14H, m), 0.87 (3H, t, *J* = 7.2 Hz). ^13^C NMR (100 MHz, CDCl_3_) δ (ppm): 172.8, 172.5, 162.4, 160.0, 145.4, 138.0, 136.1, 129.7, 129.6, 124.0, 117.2, 115.1, 114.9, 78.2, 75.2, 71.4, 37.0, 34.9, 34.3, 32.7, 31.8, 31.2, 29.4, 29.3, 29.2, 28.7, 27.6, 25.8, 24.9, 22.7, 18.0, 14.0.

#### (S)-(S)-1-((1R,5S)-1-(3-methylbut-2-en-1-yl)-7-oxo-6-oxabicyclo[3.2.0]hept-2-en-3-yl)heptyl- 2-formamido-3-methylbutanoate (**B29**)

Yield: 81 %. HR-EI-MS *m/z*: 419.2655 [M]^+^ (Calcd. for C_24_H_37_NO_5_: 419.2672). ^1^H NMR (400 MHz, CDCl_3_) δ (ppm): 8.27 (1H, brs), 6.11 (1H, brs), 5.65 (1H, s), 5.50 (1H, m), 5.06 (1H, m), 4.77 (1H, brs), 4.67 (1H, m), 2.70-2.58 (3H, m), 2.43 (1H, m), 2.20 (1H, m), 1.71 (3H, s), 1.63 (3H, s), 1.26 (10H, s), 0.97 (6H, m), 0.89 (3H, m). ^13^C NMR (100 MHz, CDCl_3_) δ (ppm): 172.3, 170.9, 160.9, 144.2, 136.2, 125.9, 117.0, 77.8, 75.1, 73.5, 55.5, 36.3, 35.9, 32.8, 31.5, 31.4, 28.8, 27.5, 25.8, 24.9, 22.5, 19.1, 18.0, 17.5, 17.3, 14.0.

#### (R)-1-((1R,5S)-1-(3-methylbut-2-en-1-yl)-7-oxo-6-oxabicyclo[3.2.0]hept-2-en-3-yl)nonylpalmitate (**B30**)

Yield: 90 %. HR-ESI-MS *m/z*: 581.4546 [M + Na]^+^ (Calcd. for C_36_H_62_O_4_Na: 581.4546). ^1^H NMR (400 MHz, CDCl_3_) δ (ppm): 5.53 (1H, s), 5.37 (1H, t, *J* = 6.6 Hz), 5.08 (1H, m), 4.75 (1H, brs), 2.73 (2H, m), 2.55 (1H, m), 2.41 (1H, m), 2.30 (2H, t, *J* = 7.8 Hz), 1.71 (3H, s), 1.62 (3H, s), 1.25 (36H, s), 0.87 (6H, t, *J* = 6.6 Hz). ^13^C NMR (100 MHz, CDCl_3_) δ (ppm): 173.0, 172.6, 145.6, 136.1, 123.7, 117.2, 78.2, 75.1, 71.3, 37.1, 34.5, 32.8, 31.9, 29.7, 29.6, 29.5, 29.4, 29.3, 29.2, 27.6, 25.8, 25.1, 24.9, 22.7, 22.6, 18.0, 14.1, 14.0.

#### (S)-(R)-1-((1R,5S)-1-(3-methylbut-2-en-1-yl)-7-oxo-6-oxabicyclo[3.2.0]hept-2-en-3-yl)heptyl-2-formamido-3-methylbutanoate (**B31**)

Yield: 82 %. HR-EI-MS *m/z*: 419.2665 [M]^+^ (Calcd. for C_24_H_37_O_5_N: 419.2672). ^1^H NMR (400 MHz, CDCl_3_) δ (ppm): 8.15 (1H, s), 5.67 (2H, m), 5.49 (1H, m), 5.14 (1H, m), 4.44 (1H, m), 2.83 (2H, m), 2.62 (1H, m), 2.45 (1H, m), 2.20 (1H, m), 1.74 (3H, s), 1.67 (3H, s), 1.33 (11H, m), 0.99 (9H, m). ^13^C NMR (100 MHz, CDCl_3_) δ (ppm): 173.0, 170.4, 144.9, 144.6, 135.3, 125.3, 124.5, 117.2, 78.5, 75.0, 72.6, 56.5, 56.3, 36.3, 36.0, 32.0, 31.5, 30.3, 28.5, 27.0, 24.6, 22.2, 18.2, 16.7, 13.0.

#### (R)-1-((1R,5S)-1-(3-methylbut-2-en-1-yl)-7-oxo-6-oxabicyclo[3.2.0]hept-2-en-3-yl)nonyltetradecanoate (**B32**)

Yield: 92 %. HR-ESI-MS *m/z*: 553.4229 [M + Na]^+^ (Calcd. for C_34_H_58_O_4_Na: 553.4233). ^1^H NMR (400 MHz, CDCl_3_) δ (ppm): 5.54 (1H, s), 5.38 (1H, t, *J* = 6.6 Hz), 5.09 (1H, m), 4.76 (1H, brs), 2.73 (2H, m), 2.58 (1H, m), 2.41 (1H, m), 2.32 (2H, t, *J* = 7.8 Hz), 1.71 (3H, s), 1.62 (3H, s), 1.26 (36H, s), 0.88 (6H, t, *J* = 6.6 Hz). ^13^C NMR (100 MHz, CDCl_3_) δ (ppm): 173.0, 172.6, 145.6, 136.1, 123.7, 117.2, 78.2, 75.1, 71.3, 37.1, 34.5, 32.8, 31.9, 29.7, 29.6, 29.5, 29.4, 29.3, 29.2, 27.6, 26.0, 25.8, 25.1, 24.9, 22.7, 22.5, 18.0, 14.1.

#### (Z)-(R)-1-((1R,5S)-1-(3-methylbut-2-en-1-yl)-7-oxo-6-oxabicyclo[3.2.0]hept-2-en-3-yl)nonyldodec-5-enoate (**B33**)

Yield: 85 %. HR-ESI-MS *m/z*: 523.3760 [M + Na]^+^ (Calcd. for C_32_H_52_O_4_Na: 523.3763). ^1^H NMR (400 MHz, CDCl_3_) δ (ppm): 5.54 (1H, s), 5.43 (2H, m),5.35 (1H, m), 5.08 (1H, m), 4.76 (1H, brs), 2.73 (2H, m), 2.58 (1H, m), 2.41 (1H, m), 2.32 (2H, t, *J* = 7.6 Hz), 2.10-2.00 (4H, m), 1.71 (3H, s), 1.68 (6H, m), 1.62 (3H, s), 1.26 (16H, s), 0.88 (6H, s). ^13^C NMR (100 MHz, CDCl_3_) δ (ppm): 172.8, 172.5, 145.5, 136.1, 131.3, 128.2, 123.8, 117.2, 78.2, 75.1, 71.4, 37.1, 33.9, 32.8, 31.8, 31.8, 29.7, 29.4, 29.2, 29.0, 27.6, 27.3, 26.6, 25.8, 25.0, 22.6, 18.0, 14.0.

#### (S)-1-((1R,5S)-1-(3-methylbut-2-en-1-yl)-7-oxo-6-oxabicyclo[3.2.0]hept-2-en-3-yl)heptyl tetradecanoate (**B34**)

Yield: 80 %. HR-EI-MS *m/z*: 502.4028 [M]^+^ (Calcd. for C_32_H_54_O_4_: 502.4022). ^1^H NMR (400 MHz, CDCl_3_) δ (ppm): 5.60 (1H, s), 5.46 (1H, t, *J* = 6.6 Hz), 5.11 (1H, m), 4.77 (1H, brs), 2.74 (2H, m), 2.63 (1H, m), 2.43 (1H, m), 2.32 (2H, t, *J* = 7.8 Hz), 1.73 (3H, s), 1.65 (3H, s), 1.27 (32H, s), 0.88 (6H, t, *J* = 6.6 Hz). ^13^C NMR (100 MHz, CDCl_3_) δ (ppm): 172.9, 172.6, 145.1, 136.1, 124.5, 117.2, 77.9, 75.1, 71.8, 36.4, 34.5, 32.8, 31.9, 31.6, 29.7, 29.6, 29.5, 29.4, 29.3, 29.2, 28.9, 27.6, 26.0, 25.8, 25.1, 24.9, 22.7, 22.5, 18.0, 14.1, 14.0.

#### (R)-1-((1R,5S)-1-(3-methylbut-2-en-1-yl)-7-oxo-6-oxabicyclo[3.2.0]hept-2-en-3-yl)heptyl tetradecanoate (**B35**)

Yield: 83 %. HR-EI-MS *m/z*: 502.4010 [M]^+^ (Calcd. for C_32_H_54_O_4_: 502.4022). ^1^H NMR (600 MHz, CDCl_3_) δ (ppm): 5.51 (1H, s), 5.36 (1H, t, *J* = 6.6 Hz), 5.06 (1H, m), 4.75 (1H, brs), 2.71 (2H, s), 2.56 (1H, m), 2.37 (1H, m), 2.29 (2H, t, *J* = 7.8 Hz), 1.69 (3H, s), 1.62 (3H, s), 1.25 (32H, s), 0.85 (6H, t, *J* = 6.6 Hz). ^13^C NMR (150 MHz, CDCl_3_) δ (ppm): 173.3, 172.9, 145.7, 136.3, 123.9, 117.4, 78.5, 75.3, 71.5, 37.3, 34.7, 33.5, 32.9, 32.1, 31.9, 29.9, 29.8, 29.7, 29.6, 29.5, 29.4, 29.3, 29.2, 27.8, 26.0, 25.3, 25.2, 24.9, 22.8, 18.3, 14.0.

#### (R)-1-((1R,5S)-1-(3-methylbut-2-en-1-yl)-7-oxo-6-oxabicyclo[3.2.0]hept-2-en-3-yl)heptyl palmitate (**B36**)

Yield: 85 %. HR-EI-MS *m/z*: 530.4354 [M]^+^ (Calcd. for C_34_H_58_O_4_: 530.4335). ^1^H NMR (600 MHz, CDCl_3_) δ (ppm): 5.51 (1H, s), 5.36 (1H, t, *J* = 6.6 Hz), 5.04 (1H, m), 4.75 (1H, brs), 2.71 (2H, s), 2.56 (1H, m), 2.37 (1H, m), 2.29 (2H, t, *J* = 7.8 Hz), 1.69 (3H, s), 1.62 (3H, s), 1.25 (32H, s), 0.85 (6H, t, *J* = 6.6 Hz). ^13^C NMR (150 MHz, CDCl_3_) δ (ppm): 173.3, 172.9, 145.7, 136.3, 123.9, 117.4, 78.5, 75.3, 71.5, 37.3, 34.7, 33.5, 32.9, 32.1, 31.9, 29.9, 29.8, 29.7, 29.6, 29.5, 29.4, 29.3, 29.2, 27.8, 26.0, 25.3, 25.2, 24.9, 22.8, 18.3, 14.3.

#### (S)-1-((1R,5S)-1-(3-methylbut-2-en-1-yl)-7-oxo-6-oxabicyclo[3.2.0]hept-2-en-3-yl)heptyl palmitate (**B37**)

Yield: 85 %. HR-EI-MS *m/z*: 530.4327 [M]^+^ (Calcd. for C_34_H_58_O_4_: 530.4335). ^1^H NMR (400 MHz, CDCl_3_) δ (ppm): 5.51 (1H, s), 5.36 (1H, t, *J* = 6.6 Hz), 5.03 (1H, m), 4.68 (1H, d, *J* = 4.0 Hz), 2.66 (2H, m), 2.52 (1H, m), 2.34 (1H, m), 2.24 (2H, t, *J* = 7.8 Hz), 1.64 (3H, s), 1.56 (3H, s), 1.19 (32H, s), 0.80 (6H, t, *J* = 6.6 Hz). ^13^C NMR (100 MHz, CDCl_3_) δ (ppm): 172.9, 172.6, 145.1, 136.1, 124.5, 117.2, 77.9, 75.1, 71.8, 36.4, 34.5, 32.8, 31.9, 31.6, 29.7, 29.6, 29.5, 29.4, 29.3, 29.2, 28.9, 27.6, 25.8, 25.1, 24.9, 22.7, 18.0, 14.1, 14.0.

### General Procedure to Synthesize Compounds **C1**–**C13**

A solution of the vibralactone (208 mg, 1.0 mmol) in acetone (5 mL) was cooled to 0 °C, and Jones reagent (0.8 mL, 2 mmol, 2.5 M) was slowly added. Stirring was continued for 1 h at 0 °C. Sodium bisulfite was added in small portions until the brown color of chromic acid disappeared. The aqueous layer was extracted with Et_2_O (3 × 30 mL). The combined organic extracts were washed with brine, dried over Na_2_SO_4_, filtered and concentrated *in vacuo*. The resultant oil was purified by chromatography on silica gel using petroleum ether/ethyl acetate (4:1) as the eluent, yielding the title compound as a colorless oil (199 mg, 90 %).

To a solution of vibralactone acid (22 mg, 0.1 mmol) in dichloromethane (2 mL) at 0 °C was added COCl_2_ (0.25 mmol) and DMF (1 μL). The reaction was monitored by TLC, and following the complete reaction of the starting material, the reaction mixture was concentrated to yield a brown–yellow oil. To a solution of diamine (0.15 mmol) in dichloromethane (2 mL) at 0 °C was added Et_3_N (0.2 mmol) and the corresponding acyl chloride dissolved in dichloromethane (2 mL). The reaction mixture was stirred at room temperature overnight, washed with 5 % HCl (2 × 10 mL), saturated NaHCO_3_ (2 × 10 mL), and brine (2 × 10 mL), and then dried (Na_2_SO_4_) and concentrated. The crude oil was purified by flash chromatography on silica gel using petroleum ether/ethyl acetate as the eluent to yield the compound.

#### (1R,5S)-N-heptyl-1-(3-methylbut-2-en-1-yl)-N-octyl-7-oxo-6-oxabicyclo[3.2.0]hept-2-ene-3-carboxamide (**C1**)

Yield: 58 %. HR-ESI-MS *m/z* 454.3289 [M + Na]^+^ (Calc. for C_27_H_45_NNaO_3_, 454.3297). ^1^H-NMR (CDCl_3_, 400 MHz) δ (ppm): 5.69 (1H, br s), 5.13 (1H, t, *J* = 7.4 Hz), 4.81 (1H, d, *J* = 5.6 Hz), 3.36 (2H, t), 3.22 (2H, m), 3.13 (1H, ddd), 2.97 (1H, dd), 2.63 (1H, dd), 2.49 (1H, dd), 1.73 (3H, s), 1.64 (3H, s), 1.27 (22H, br s), 0.88 (6H, t, *J* = 6.6 Hz). ^13^C-NMR (CDCl_3_, 100 MHz) δ (ppm): 171.8, 166.7, 140.3, 136.5, 126.5, 116.9, 76.2, 48.6, 44.9, 39.5, 31.7, 29.7, 29.2, 29.0, 27.4, 27.3, 27.0, 26.7, 25.8, 22.6, 22.58, 18.0, 14.07, 14.05.

#### (1R,5S)-1-(3-methylbut-2-en-1-yl)-N,N-dioctyl-7-oxo-6-oxabicyclo[3.2.0]hept-2-ene-3-carboxamide (**C2**)

Yield: 56 %. HR-EI-MS *m/z*: 445.3564 [M]^+^ (Calcd. for C_28_H_47_NO_3_: 445.3556). ^1^H NMR (400 MHz, CDCl_3_) δ (ppm): 5.69 (1H, s), 5.14 (1H, t, *J* = 6.5 Hz), 4.81 (1H, d, *J* = 5.6 Hz), 3.34 (2H, m), 3.22 (2H, m), 3.16-3.09 (2H, m), 2.63 (1H, m), 2.48 (1H, m), 1.73 (3H, s), 1.64 (3H, s), 1.52 (4H, s), 1.26 (20H, s), 0.89 (6H, t, *J* = 4.0 Hz). ^13^C NMR (100 MHz, CDCl_3_) δ (ppm): 171.8, 166.7, 140.3, 136.5, 126.5, 116.9, 77.6, 76.2, 50.0, 48.7, 39.5, 31.8, 29.7, 29.5, 29.2, 27.5, 27.3, 27.0, 26.8, 25.8, 22.6, 18.1, 14.1.

#### (1R,5S)-N,N-diheptyl-1-(3-methylbut-2-en-1-yl)-7-oxo-6-oxabicyclo[3.2.0]hept-2-ene-3-carboxamide (**C3**)

Yield: 60 %. HR-EI-MS *m/z*: 417.3257 [M]^+^ (Calcd. for C_26_H_43_NO_3_: 417.3243). ^1^H NMR (400 MHz, CDCl_3_) δ (ppm): 5.71 (1H, s), 5.15 (1H, m), 4.83 (1H, d, *J* = 5.6 Hz), 3.36 (2H, m), 3.26 (2H, m), 3.18-3.10 (1H, m), 3.01 (1H, m), 2.66 (1H, m), 2.51 (1H, m), 1.73 (3H, s), 1.64 (3H, s), 1.52 (4H, m), 1.26 (16H, m), 0.89 (6H, t, *J* = 6.8 Hz). ^13^C NMR (100 MHz, CDCl_3_) δ (ppm): 171.7, 166.7, 140.3, 136.5, 126.5, 116.9, 77.6, 76.2, 48.6, 44.9, 39.5, 31.7, 29.7, 29.2, 29.0, 28.9, 27.5, 27.3, 27.0, 26.7, 25.8, 22.6, 18.0, 14.1.

#### (1R,5S)-1-(3-methylbut-2-en-1-yl)-N-octyl-7-oxo-N-pentyl-6-oxabicyclo[3.2.0]hept-2-ene-3-carboxamide (**C4**)

Yield: 64 %. HR-EI-MS *m/z*: 403.3094 [M]^+^ (Calcd. for C_25_H_41_NO_3_: 403.3086). ^1^H NMR (400 MHz, CDCl_3_) δ (ppm): 5.69 (1H, s), 5.14 (1H, m), 4.81 (1H, d, *J* = 5.6 Hz), 3.35 (2H, m), 3.24 (2H, m), 3.16-3.10 (1H, m), 2.96 (1H, m), 2.64 (1H, m), 2.48 (1H, m), 1.73 (3H, s), 1.64 (3H, s), 1.50 (4H, m), 1.26 (14H, m), 0.89 (6H, t, *J* = 6.8 Hz). ^13^C NMR (100 MHz, CDCl_3_) δ (ppm): 171.7, 166.7, 140.3, 136.5, 126.5, 116.9, 77.6, 76.2, 48.7, 44.9, 39.5, 31.8, 29.2, 28.9, 27.4, 27.3, 27.1, 27.0, 26.7, 25.8, 22.6, 22.4, 18.0, 14.1.

#### (1R,5S)-1-(3-methylbut-2-en-1-yl)-N,N-dinonyl-7-oxo-6-oxabicyclo[3.2.0]hept-2-ene-3-carboxamide (**C5**)

Yield: 68 %. HR-EI-MS *m/z*: 473.3871 [M]^+^ (Calcd. for C_30_H_51_NO_3_: 473.3869). ^1^H NMR (400 MHz, CDCl_3_) δ (ppm): 5.69 (1H, s), 5.14 (1H, m), 4.81 (1H, d, *J* = 5.6 Hz), 3.35 (2H, m), 3.23 (2H, m), 3.16-3.10 (1H, m), 2.99 (1H, m), 2.64 (1H, m), 2.48 (1H, m), 1.73 (3H, s), 1.64 (3H, s), 1.50 (4H, m), 1.26 (24H, m), 0.89 (6H, t, *J* = 6.8 Hz). ^13^C NMR (100 MHz, CDCl_3_) δ (ppm): 171.8, 166.7, 140.3, 136.5, 126.5, 116.9, 77.6, 76.2, 48.7, 44.9, 39.5, 31.9, 29.7, 29.6, 29.4, 29.2, 27.4, 25.8, 22.6, 18.0, 14.1.

#### (1R,5S)-N,N-dihexyl-1-(3-methylbut-2-en-1-yl)-7-oxo-6-oxabicyclo[3.2.0]hept-2-ene-3-carboxamide (**C6**)

Yield: 70 %. HR-EI-MS *m/z*: 389.2923 [M]^+^ (Calcd. for C_24_H_39_NO_3_: 389.2930). ^1^H NMR (400 MHz, CDCl_3_) δ (ppm): 5.69 (1H, s), 5.13 (1H, m), 4.81 (1H, d, *J* = 5.6 Hz), 3.35 (2H, m), 3.24 (2H, m), 3.16-3.10 (1H, m), 2.96 (1H, m), 2.64 (1H, m), 2.48 (1H, m), 1.72 (3H, s), 1.63 (3H, s), 1.50 (4H, m), 1.26 (12H, m), 0.89 (6H, t, *J* = 6.8 Hz). ^13^C NMR (100 MHz, CDCl_3_) δ (ppm): 171.8, 166.7, 140.3, 136.5, 126.5, 116.9, 77.6, 76.2, 48.7, 44.9, 39.5, 31.9, 29.7, 29.6, 29.4, 29.2, 27.4, 25.8, 22.6, 18.0, 14.1.

#### (1R,5S)-N,N-didecyl-1-(3-methylbut-2-en-1-yl)-7-oxo-6-oxabicyclo[3.2.0]hept-2-ene-3-carboxamide (**C7**)

Yield: 65 %. HR-EI-MS *m/z*: 501.4168 [M]^+^ (Calcd. for C_32_H_55_NO_3_: 501.4182). ^1^H NMR (400 MHz, CDCl_3_) δ (ppm): 5.69 (1H, s), 5.13 (1H, m), 4.81 (1H, d, *J* = 4.0 Hz), 3.35 (2H, m), 3.26 (3H, m), 3.16-3.09 (1H, m), 2.99 (1H, m), 2.65 (1H, m), 2.48 (1H, m), 1.73 (3H, s), 1.64 (3H, s), 1.52 (4H, s), 1.26 (28H, s), 0.89 (6H, t, *J* = 8.0 Hz). ^13^C NMR (100 MHz, CDCl_3_) δ (ppm): 171.8, 166.7, 140.3, 136.5, 126.5, 116.9, 77.6, 76.2, 57.9, 48.7, 44.9, 39.5, 31.9, 29.6, 29.5, 29.3, 27.5, 27.3, 27.0, 26.8, 25.8, 22.6, 18.1, 14.1.

#### (1R,5S)-1-(3-methylbut-2-en-1-yl)-7-oxo-N,N-dipentyl-6-oxabicyclo[3.2.0]hept-2-ene-3-carboxamide (**C8**)

Yield: 63 %. HR-EI-MS *m/z*: 361.2627 [M]^+^ (Calcd. for C_22_H_35_NO_3_: 361.2617). ^1^H NMR (400 MHz, CDCl_3_) δ (ppm): 5.70 (1H, s), 5.13 (1H, m), 4.81 (1H, d, *J* = 5.6 Hz), 3.36 (2H, m), 3.26 (2H, m), 3.16-3.09 (1H, m), 3.01 (1H, m), 2.68 (1H, m), 2.50 (1H, m), 1.73 (3H, s), 1.64 (3H, s), 1.52 (4H, m), 1.26 (8H, m), 0.89 (6H, t, *J* = 6.8 Hz). ^13^C NMR (100 MHz, CDCl_3_) δ (ppm): 171.8, 166.7, 140.3, 136.5, 126.5, 116.9, 77.6, 76.2, 49.9, 48.6, 44.9, 39.5, 32.9, 29.2, 28.8, 28.6, 27.3, 27.1, 25.8, 22.3, 18.0, 14.0.

#### (1R,5S)-1-(3-methylbut-2-en-1-yl)-7-oxo-N,N-diundecyl-6-oxabicyclo[3.2.0]hept-2-ene-3-carboxamide (**C9**)

Yield: 70 %. HR-EI-MS *m/z*: 529.4467 [M]^+^ (Calcd. for C_34_H_59_NO_3_: 529.4495). ^1^H NMR (600 MHz, CDCl_3_) δ (ppm): 5.70 (1H, s), 5.13 (1H, m), 4.81 (1H, d, *J* = 5.6 Hz), 3.35 (2H, m), 3.24 (2H, m), 3.16-3.11 (1H, m), 3.10 (1H, m), 2.65 (1H, m), 2.48 (1H, m), 1.73 (3H, s), 1.64 (3H, s), 1.52 (4H, m), 1.26 (32H, m), 0.89 (6H, t, *J* = 8.0 Hz). ^13^C NMR (150 MHz, CDCl_3_) δ (ppm): 171.8, 166.7, 140.2, 136.6, 126.5, 116.9, 77.6, 76.2, 50.1, 48.6, 44.9, 39.5, 31.9, 29.7, 29.6, 29.5, 29.4, 29.3, 29.2, 27.7, 27.4, 27.3, 27.0, 26.8, 25.8, 22.7, 18.1, 14.1.

#### (1R,5S)-N,N-didodecyl-1-(3-methylbut-2-en-1-yl)-7-oxo-6-oxabicyclo[3.2.0]hept-2-ene-3-carboxamideCompound (**C10**)

Yield: 76 %. ESI-MS *m/z*: 580 [M + Na]^+^. ^1^H NMR (400 MHz, CDCl_3_) δ (ppm): 5.70 (1H, s), 5.13 (1H, m), 4.81 (1H, d, *J* = 5.6 Hz), 3.33 (2H, m), 3.26 (2H, m), 3.16-3.09 (1H, m), 2.99 (1H, m), 2.65 (1H, m), 2.48 (1H, m), 1.73 (3H, s), 1.64 (3H, s), 1.52 (4H, m), 1.26 (36H, m), 0.89 (6H, t, *J* = 8.0 Hz). ^13^C NMR (100 MHz, CDCl_3_) δ (ppm): 171.8, 166.7, 140.3, 136.5, 126.5, 116.9, 77.6, 76.2, 48.7, 44.9, 39.5, 31.9, 29.6, 29.5, 29.4, 27.3, 27.0, 26.8, 25.8, 22.7, 18.1, 14.1.

#### (1R,5S)-1-(3-methylbut-2-en-1-yl)-7-oxo-N,N-ditridecyl-6-oxabicyclo[3.2.0]hept-2-ene-3-carboxamide (**C11**)

Yield: 72 %. HR-EI-MS *m/z*: 585.5109 [M]^+^ (Calcd. for C_38_H_67_NO_3_: 585.5121). ^1^H NMR (400 MHz, CDCl_3_) δ (ppm): 5.69 (1H, s), 5.15 (1H, m), 4.81 (1H, d, *J* = 5.6 Hz), 3.35 (2H, m), 3.23 (2H, m), 3.16-3.10 (1H, m), 3.00 (1H, m), 2.64 (1H, m), 2.48 (1H, m), 1.73 (3H, s), 1.64 (3H, s), 1.50 (4H, m), 1.26 (40H, m), 0.89 (6H, t, *J* = 6.8 Hz). ^13^C NMR (100 MHz, CDCl_3_) δ (ppm): 171.7, 166.7, 140.3, 136.5, 126.5, 116.9, 77.6, 76.2, 48.7, 44.9, 39.5, 31.9, 29.7, 29.6, 29.4, 29.2, 27.5, 27.3, 27.0, 26.7, 25.8, 22.6, 18.0, 14.1.

#### (1R,5S)-N-butyl-1-(3-methylbut-2-en-1-yl)-7-oxo-N-propyl-6-oxabicyclo[3.2.0]hept-2-ene-3-carboxamide (**C12**)

Yield: 74 %. HR-EI-MS *m/z*: 319.2146 [M]^+^ (Calcd. for C_19_H_29_NO_3_: 319.2147). ^1^H NMR (400 MHz, CDCl_3_) δ (ppm): 5.72 (1H, s), 5.15 (1H, m), 4.82 (1H, d, *J* = 5.6 Hz), 3.35 (2H, m), 3.25 (2H, m), 3.18-3.11 (1H, m), 2.98 (1H, m), 2.66 (1H, m), 2.50 (1H, m), 1.75 (3H, s), 1.66 (3H, s), 1.50 (4H, m), 1.26 (2H, m), 0.86 (6H, m). ^13^C NMR (100 MHz, CDCl_3_) δ (ppm): 171.8, 166.8, 140.3, 136.5, 126.5, 116.9, 77.6, 76.2, 48.4, 44.7, 39.5, 31.3, 29.6, 27.3, 25.8, 22.4, 20.2, 18.0, 14.1.

#### (1R,5S)-N,N-dibutyl-1-(3-methylbut-2-en-1-yl)-7-oxo-6-oxabicyclo[3.2.0]hept-2-ene-3-carboxamide (**C13**)

Yield: 67 %. ESI-MS *m/z*: 356 [M + Na]^+^. ^1^H NMR (400 MHz, CDCl_3_) δ (ppm): 5.70 (1H, s), 5.13 (1H, m), 4.81 (1H, d, *J* = 5.6 Hz), 3.35 (2H, m), 3.26 (2H, m), 3.16-3.09 (1H, m), 2.99 (1H, m), 2.65 (1H, m), 2.48 (1H, m), 1.73 (3H, s), 1.64 (3H, s), 1.52 (4H, m), 1.26 (4H, m), 0.89 (6H, m). ^13^C NMR (100 MHz, CDCl_3_) δ (ppm): 171.8, 166.7, 140.3, 136.5, 126.5, 116.9, 77.6, 76.2, 48.7, 44.6, 39.5, 31.9, 29.5, 28.5, 27.3, 25.8, 21.8, 19.9, 18.1, 13.6.

### Measurement of the Inhibitory Activity Against Pancreatic Lipase

Porcine pancreatic lipase was used to evaluate the inhibitory activity of the monascus pigment derivatives. *p*-Nitrophenyl butyrate (*p*-NPS) was used as the substrate. First, an enzyme-buffer solution was prepared by adding 30 μL of lipase solution (in 10 mM morpholinepropanesulfonic acid (MOPS) and 1 mM EDTA, pH 6.8) to 850 μL of Tris buffer (100 mM Tris–HCl and 5 mM CaCl_2_, pH 7.0). Pigment solutions were prepared by dissolving each pigment in a mixture (1:1) of ethanol and distilled water. Subsequently, 100 μL of the pigment solution was mixed with 880 μL of the enzyme-buffer solution. Following incubation of the enzyme-pigment mixture for 15 min at 37 °C, 20 μL of the substrate solution (10 mM *p*-NPB in dimethyl formamide) was added. Enzymatic reactions were conducted for 15 min at 37 °C. The hydrolysis of *p*-NPB to *p*-nitrophenol was monitored at 400 nm using a spectrophotometer. One unit of the enzyme was defined as the amount required to liberate 1 μmol of *p*-nitrophenol under standard assay conditions.

### In vivo Experiments

#### Animals

Male C57BL/6 J mice were obtained from Beijing HFK Bioscience Co., Ltd. (Certificate No. SCXK 2014-0004), housed in a temperature- and humidity-controlled room with a 12-hour light/dark cycle, and allowed free access to solid food and tap water for one week prior to the experiments. All of the procedures were performed in accordance with the Institute Ethical Committee for Experimental Animal Use of the Yunnan Province and the Kunming Institute of Botany.

#### Reagents

The kits used to determine the triglyceride (TG) and cholesterol (CHO) levels were obtained from the Zhongsheng Beikong Bioengineering Institute.

#### Diet-Induced Obesity in Mice

Three-week-old C57BL/6 J male mice were fed a normal diet for 1 week for acclimatization. Randomly selected mice were fed a normal diet or a 45 % fat diet. After 4 months, the body weights of the mice in the obese group were 20 % higher than those in the control group. These obese mice were randomly divided into the following three groups: the model (45 % fat diet), orlistat (45 % fat diet + orlistat) and Compound groups (45 % fat diet + compound **C1**). The drugs (50 mg/kg orlistat or 100 mg/kg compound **C1**) were intragastrically administered for 1 month. During this period, the mice were weighed once every 3 days. Following administration of the final dose, blood samples were collected via the orbital vein after ethyl ether exposure, and epididymal fat was isolated. TG and CHO levels were measured using kits. The ratio of epididymal fat to body weight represents the amount of white adipose tissue (WAT) of each mouse (normalized to the body weight).

#### Statistical Analysis

Student’s *t* test was used for comparisons between groups, as indicated in Fig. 2. #p < 0.05, ## or **p < 0.01.

## Electronic supplementary material

Supplementary material 1 (DOCX 1947 kb)
