# Supplementary material for: Structure-Based Optimization and Biological Evaluation of Pancreatic Lipase Inhibitors as Novel Potential Antiobesity Agents
Source: Nat Prod Bioprospect. 2015 Jun 18;5(3):129–57. doi: 10.1007/s13659-015-0062-6 (PMC4488150; doi:10.1007/s13659-015-0062-6)
Supplement: Supplementary file 1 — Supplementary material 1 (DOCX 1947 kb) [file 13659_2015_62_MOESM1_ESM.docx]

**Supporting Information**

**Structure-Based Optimization and Biological Evaluation of Pancreatic Lipase Inhibitors as Novel Potential Antiobesity Agents**

Kun Wei,^a,1^ Gang-Qiang Wang,^a, b, 1^ Xue Bai,^a^ Yan-Fen Niu,^a^ He-Ping Chen,^a^ Chun-Nan Wen,^a^ Zheng-Hui Li,^a^ Ze-Jun Dong,^a^ Zhi-Li Zuo,^a,*^ Wen-Yong Xiong,^a,*^ Ji-Kai Liu^a,*^

^a^ State Key Laboratory of Phytochemistry and Plant Resources in West China, Kunming Institute of Botany, Chinese Academy of Sciences, Kunming 650201, China

^b^ School of Nuclear Technology and Chemistry & Biology, Hubei University of Science and Technology, Xianning 437100, China

***** Corresponding Author. Kunming Institute of Botany, Chinese Academy of Sciences, Kunming 650201, China

Email address: [jkliu@mail.kib.ac.cn](mailto:jkliu@mail.kib.ac.cn) (J. K. Liu). [zuozhili@mail.kib.ac.cn](mailto:zuozhili@mail.kib.ac.cn) (Z. L. Zuo). [xiongwenyong@mail.kib.ac.cn](mailto:xiongwenyong@mail.kib.ac.cn) (W. Y. Xiong)

^1^ These authors contributed equally to this paper.

**Contents:**

**Copies of representative ^1^H and ^13^C NMR spectra (Compounds A1, A2, B1-B11, C1-C4)**

**Compound A1: ^1^H NMR spectrum in CDCl_3_ (500MHz).**


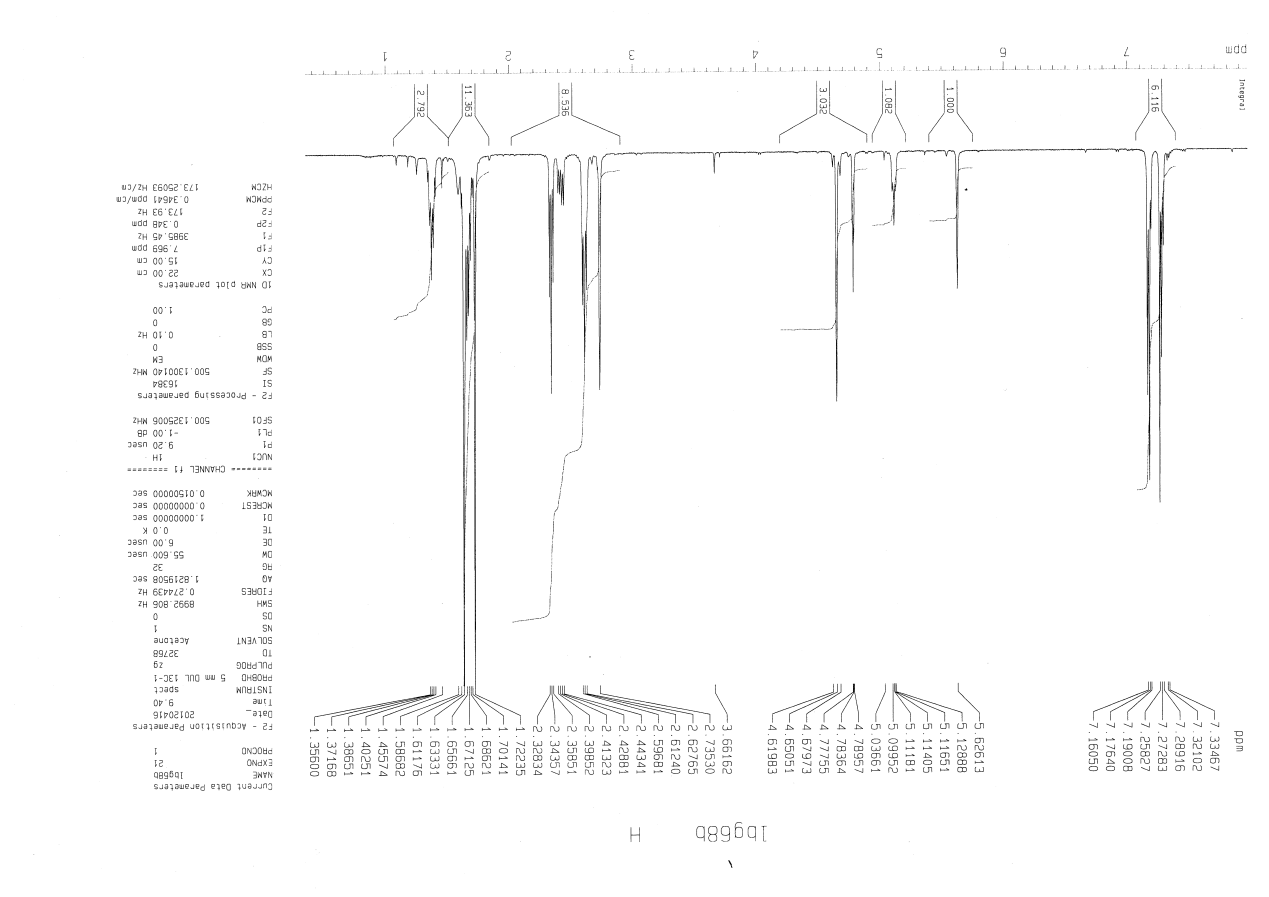

**Compound A1: ^13^C NMR and DEPTspectra in CDCl_3_(125 MHz).**


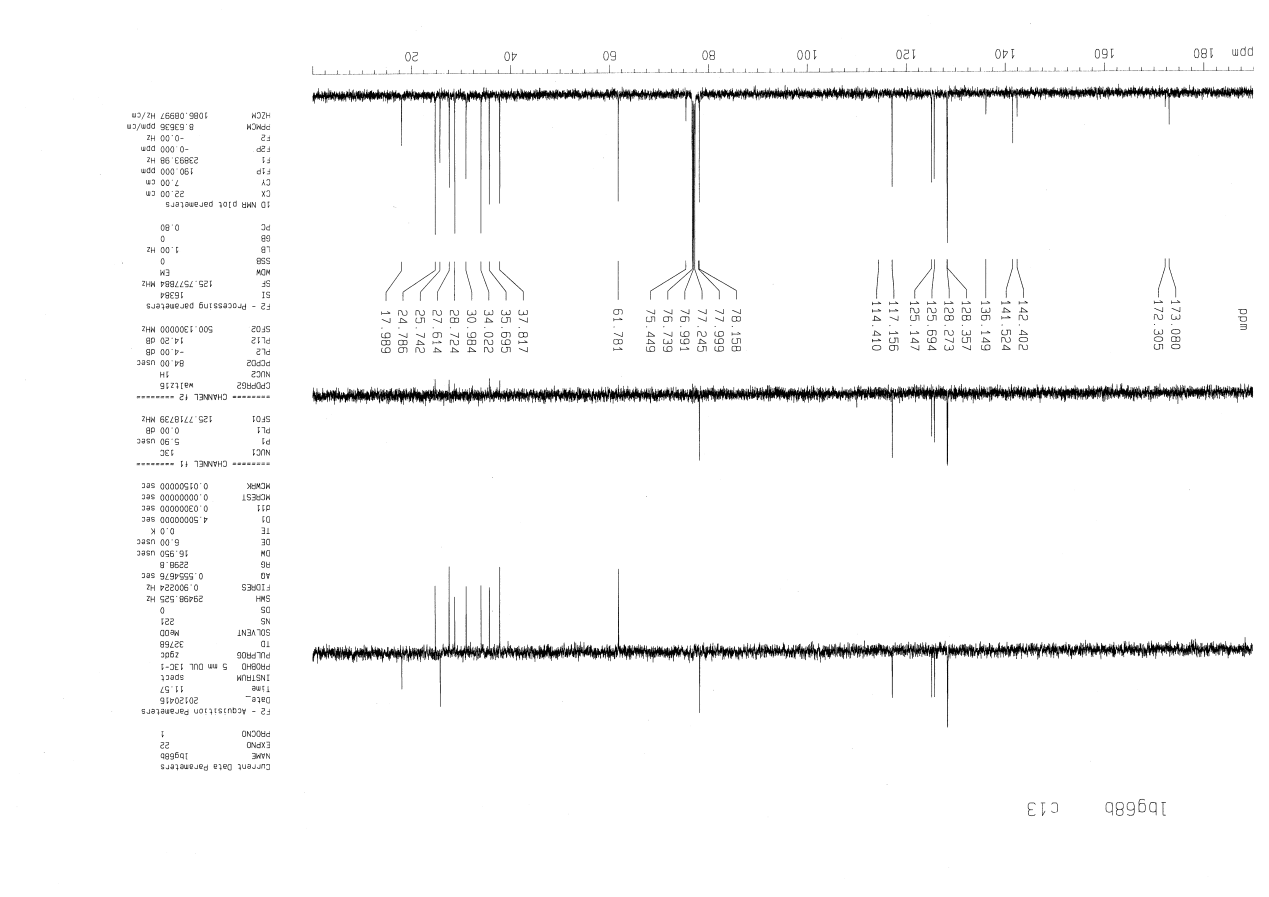

**Compound A1: HREIMS**


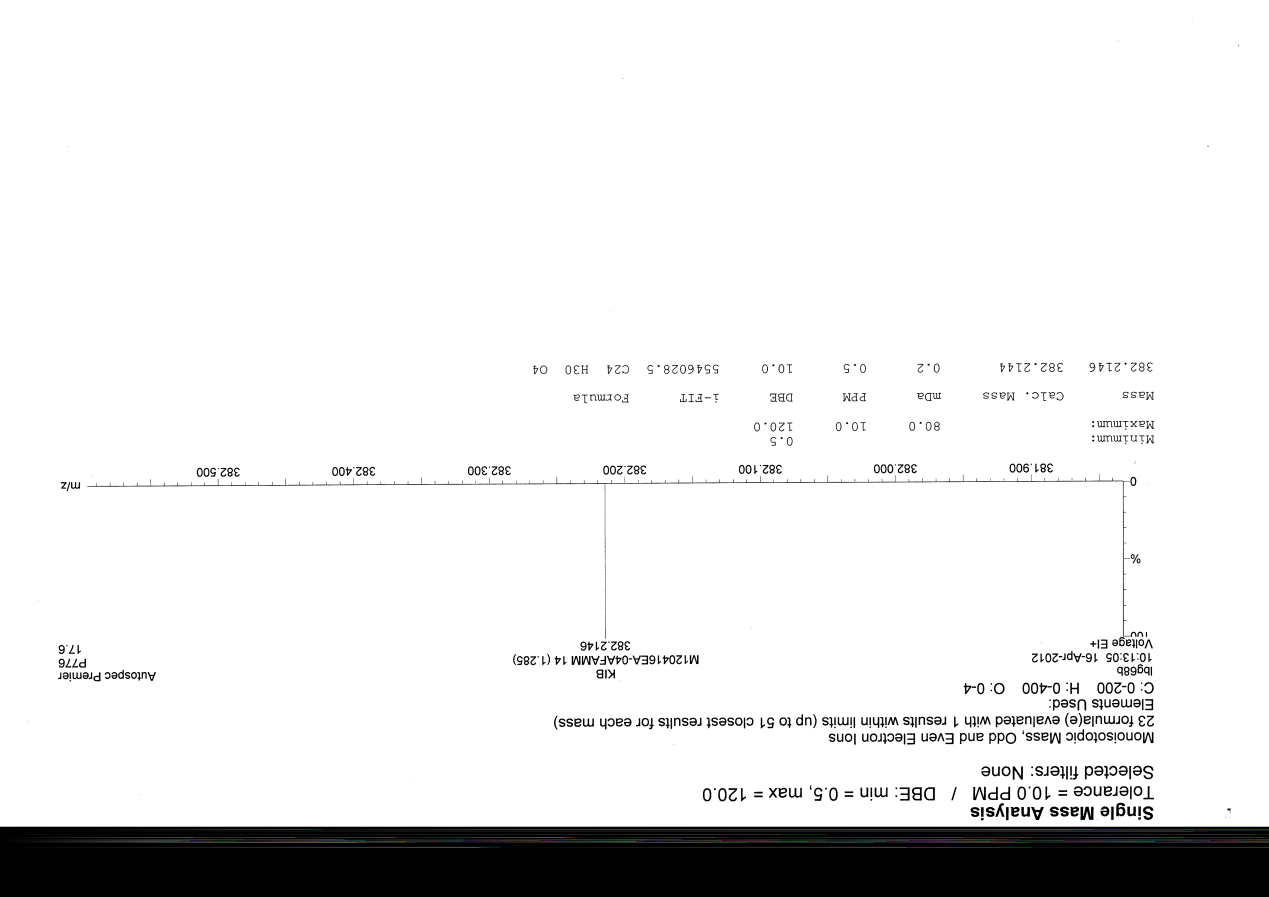


**Compound A2: ^1^H NMR in CDCl_3_(400MHz).**

**

**

**Compound A2: ^13^C NMR and DEPTspectra in CDCl_3_(100 MHz).**

**

**

**Compound B1: ^1^H NMR spectrum in CDCl_3_(400 MHz).**


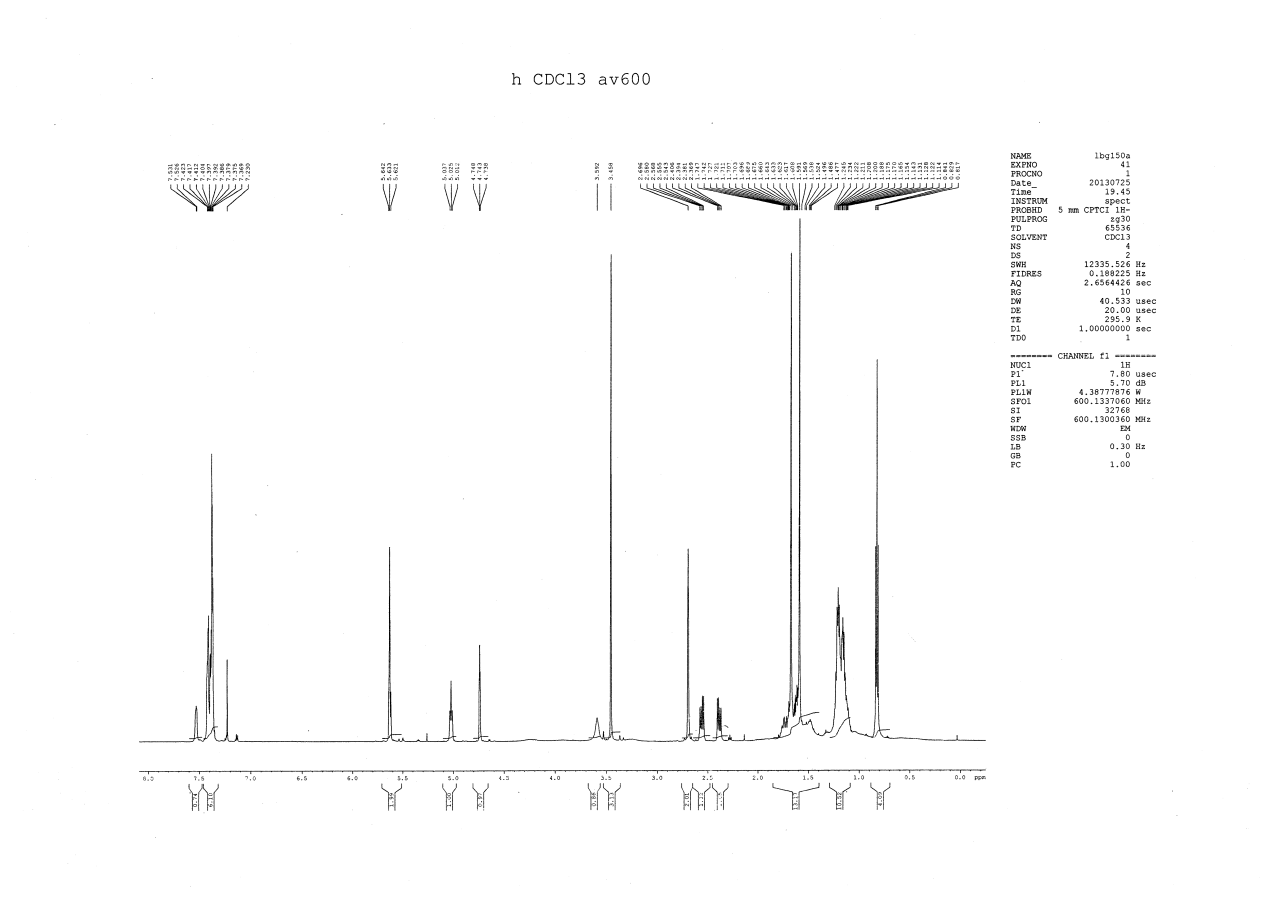

**Compound B1: ^13^C NMR and DEPT spectra in CDCl_3_(100 MHz).**


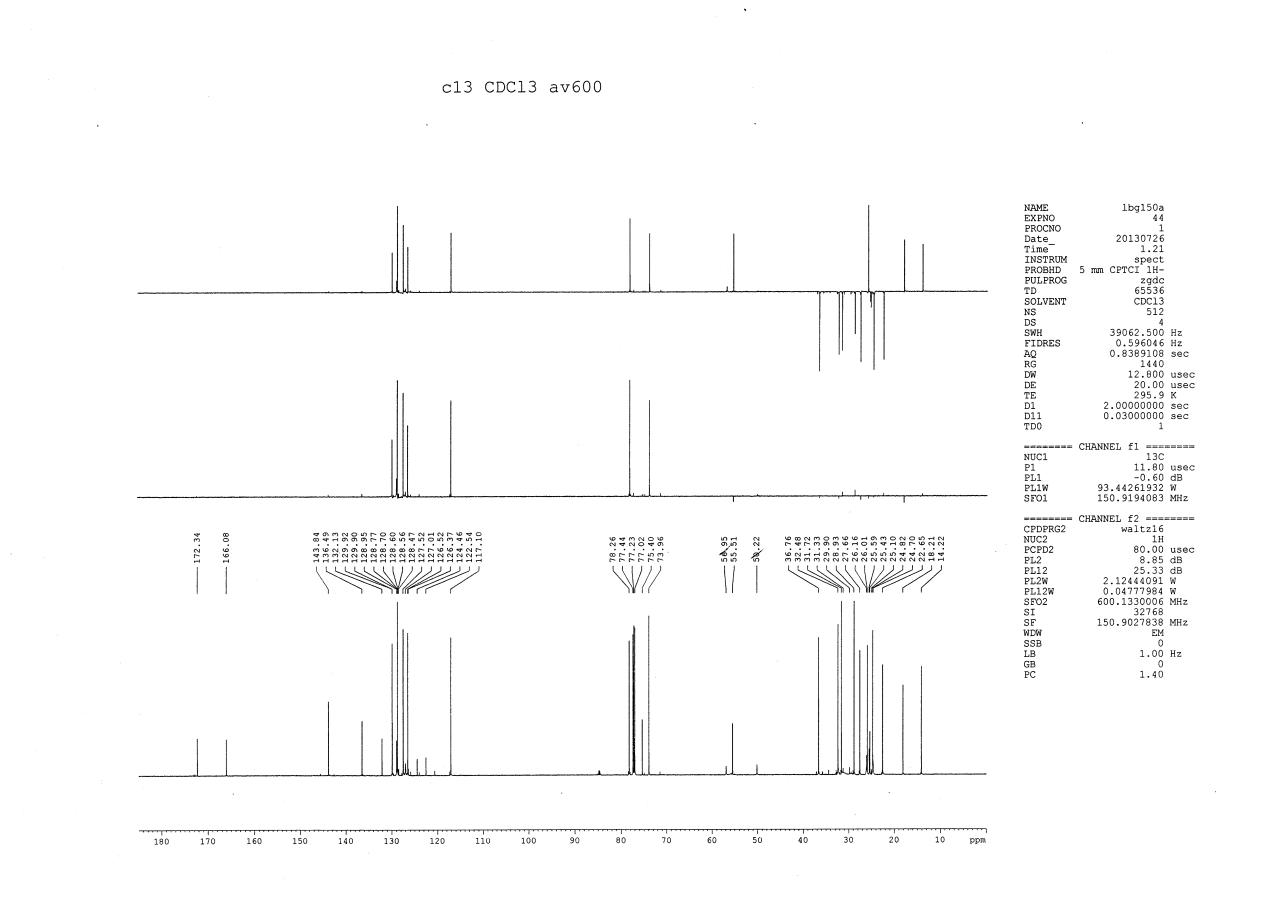

**Compound B1: HREIMS**


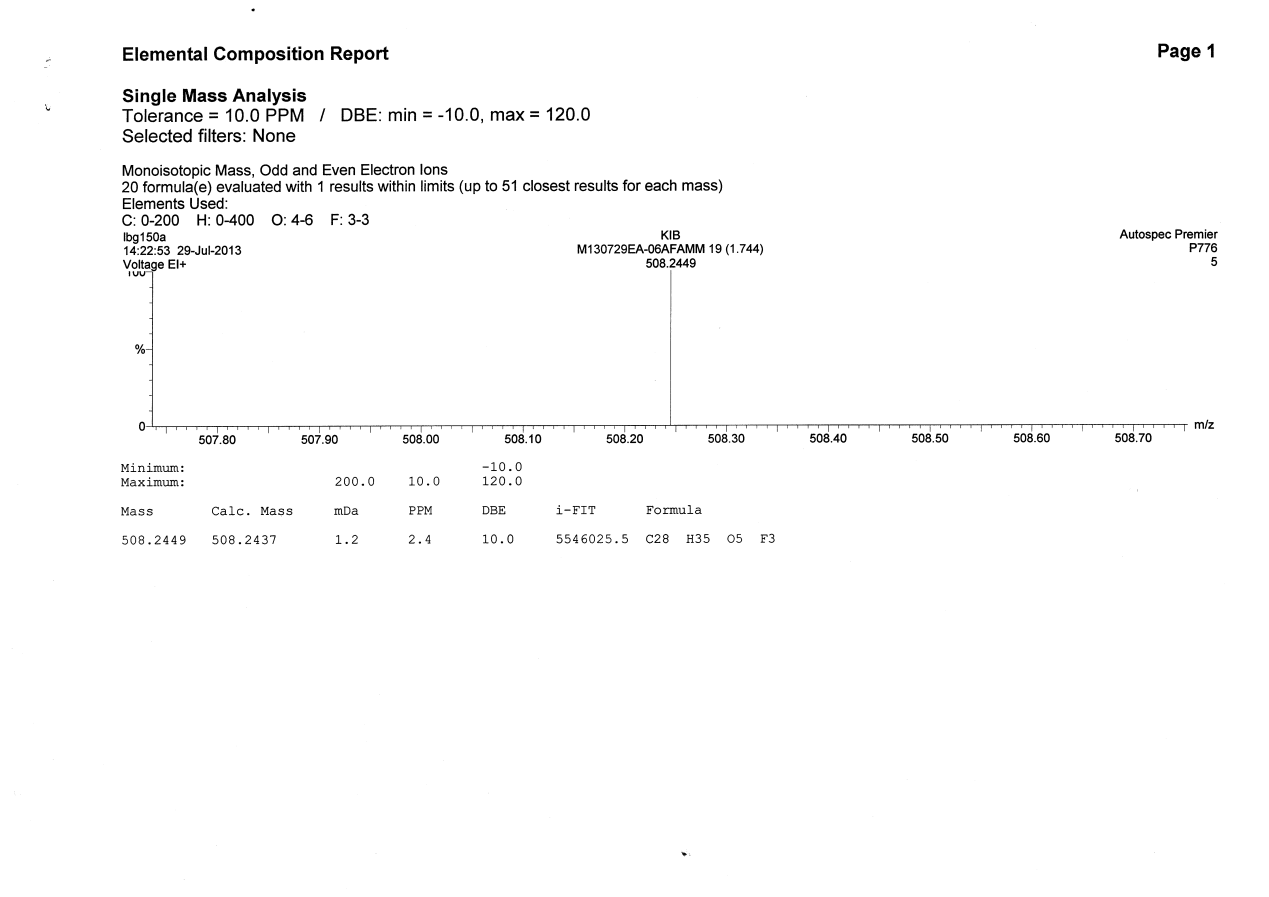


**Compound B2: ^1^H NMR spectrum in CDCl_3_(400MHz).**




**Compound B2: ^13^C NMR spectrum in CDCl_3_(100 MHz).**




**Compound B3: ^1^H NMR spectrum in CDCl_3_(600MHz).**

**

**

**Compound B3: ^13^C NMR and DEPTspectra in CDCl_3_(150 MHz).**

**

**

**Compound B4: ^1^H NMR spectrum in CDCl_3_(400MHz).**

**

**

**Compound B4: ^13^C NMR and DEPTspectra in CDCl_3_(100 MHz).**

**

**

**Compound B5: ^1^H NMR spectrum in CDCl_3_(400MHz).**

**

**

**Compound B5: ^13^C NMR spectrum in CDCl_3_(100 MHz).**

**

**

**Compound B6: ^1^H NMR spectrum in CDCl_3_(400MHz).**

**

**

**Compound B6: ^13^C NMR spectrum in CDCl_3_(100 MHz).**

**

**

**Compound B7: ^1^H NMR spectrum in CDCl_3_(500MHz).**

**

**

**Compound B7: ^13^C NMR and DEPTspectra in CDCl_3_(150 MHz).**

**

**

**Compound B8: ^1^H NMR spectrum in CDCl_3_(400MHz).**

**

**

**Compound B8: ^13^C NMR spectrum in CDCl_3_(100 MHz).**

**

**

**Compound B9: ^1^H NMR spectrum in CDCl_3_(400MHz).**

**

**

**Compound B9: ^13^C NMR and DEPTspectra in CDCl_3_(100 MHz).**

**

**

**Compound B10: ^1^H NMR spectrum in CDCl_3_(500MHz).**

**

**

**Compound B10: ^13^C NMR and DEPTspectra in CDCl_3_(100 MHz).**

**

**

**Compound B11: ^1^H NMR spectrum in CDCl_3_(400MHz).**

**

**

**Compound B11: ^13^C NMR and DEPTspectra in CDCl_3_(150 MHz).**

**

**

**Compound C1: ^1^HNMR spectrum in CDCl_3_(400 MHz).**


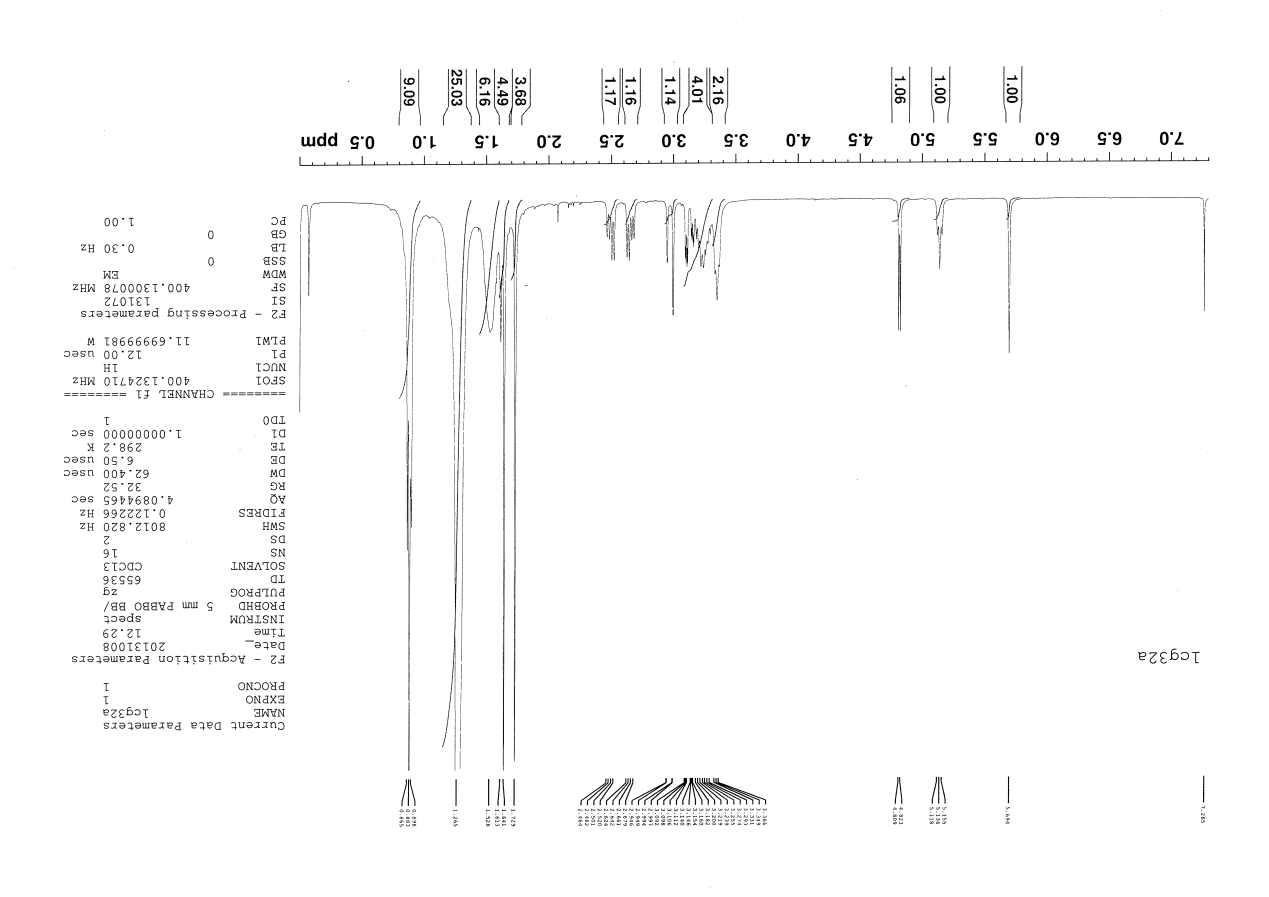

**Compound C1: ^13^C NMR spectrum in CDCl_3_(100 MHz).**


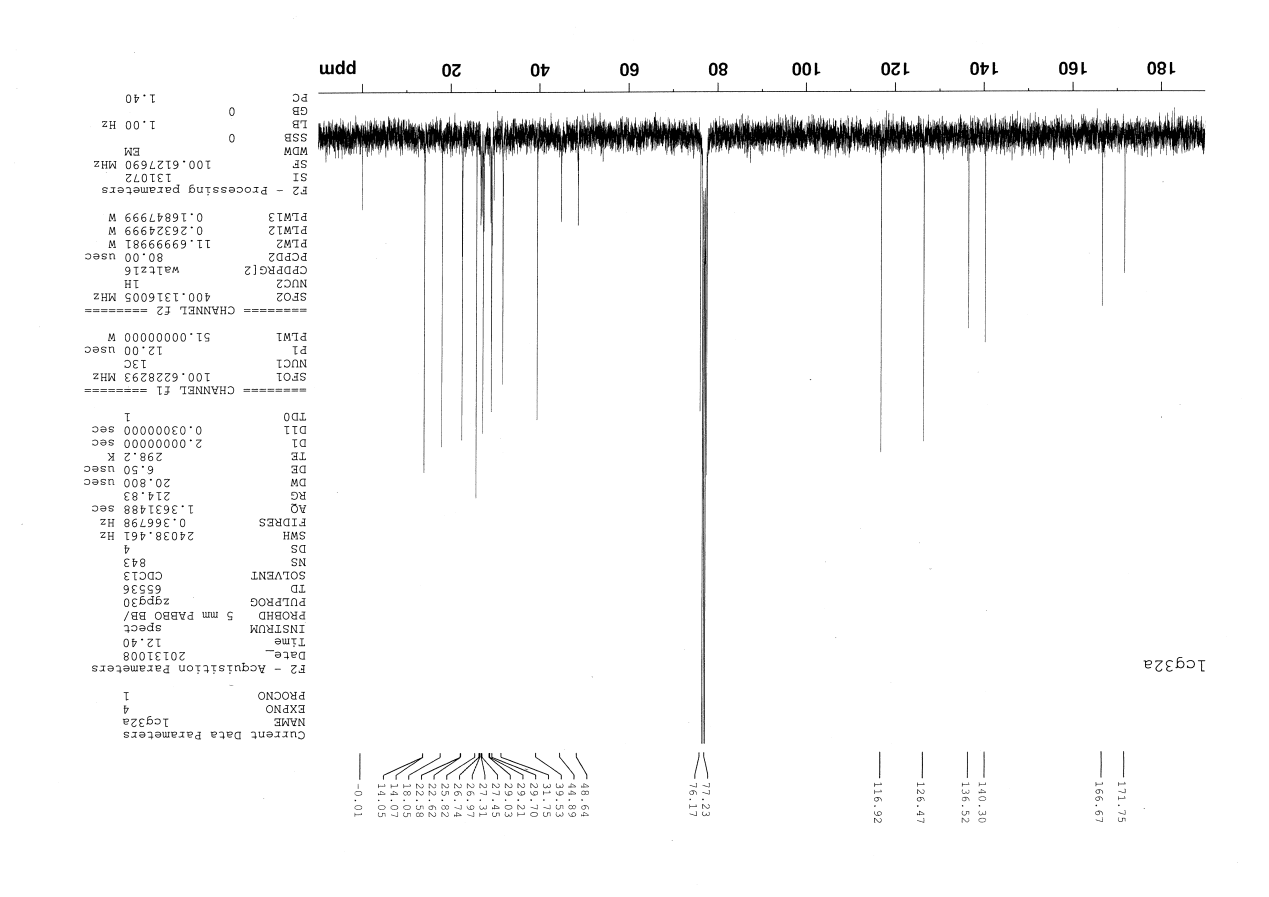

**Compound C1: HRESIMS**


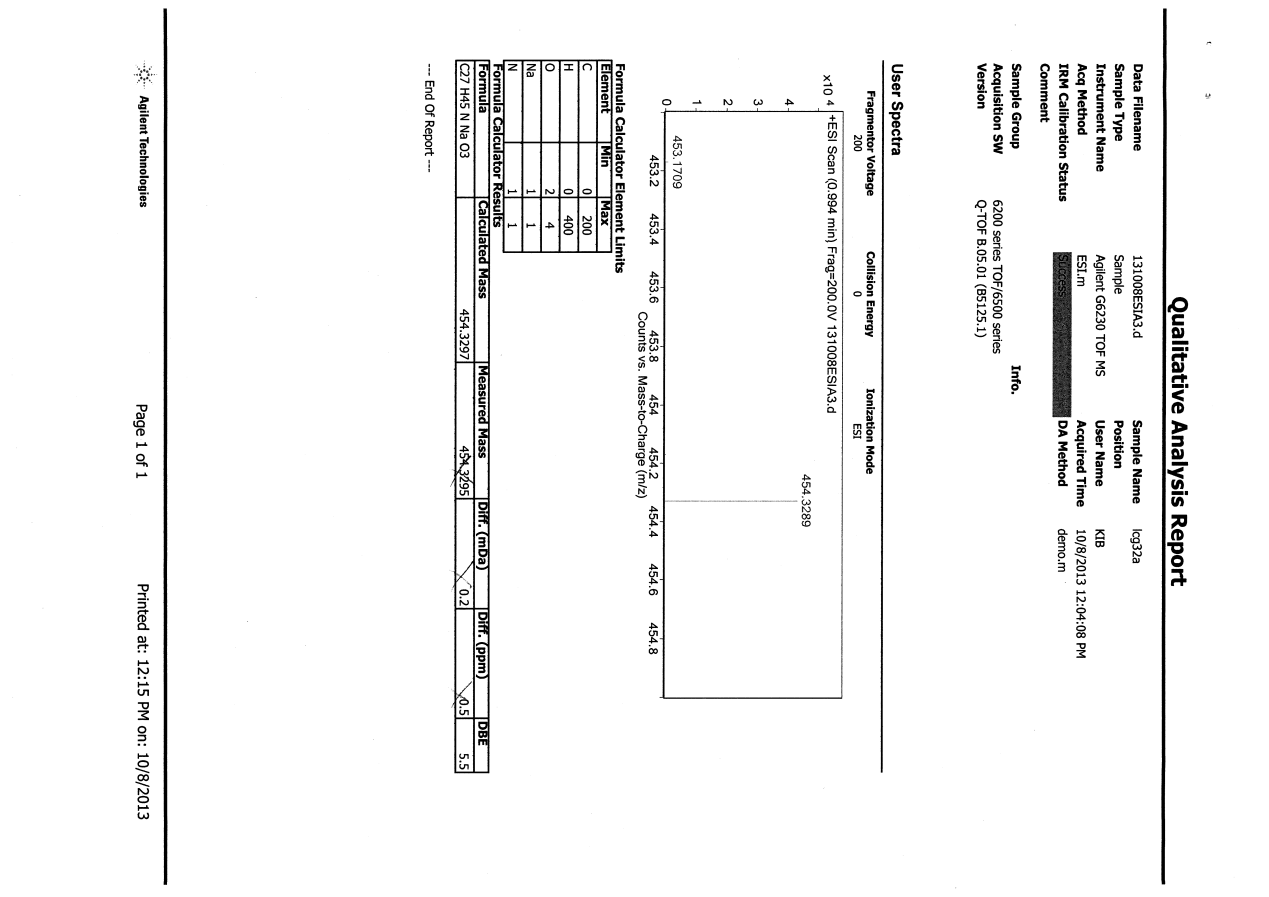


**Compound C2: ^1^H NMR spectrum in CDCl_3_(400MHz).**

**

**

**Compound C2: ^13^C NMR spectrum in CDCl_3_(100 MHz).**

**

**

**Compound C3: ^1^H NMR spectrum in CDCl_3_(400MHz).**

**

**

**Compound C3: ^13^C NMR and DEPTspectra in CDCl_3_(100 MHz).**

**

**

**Compound C4: ^1^H NMR spectrum in CDCl_3_(400MHz).**

**

**

**Compound C4: ^13^C NMR spectrum in CDCl_3_(100 MHz)**

**

**
